# Supplementary material for: 3′UTR shortening alleviates miRNA repression of mRNAs critical for muscle stem cell differentiation
Source: EMBO J. 2025 Dec 22;45(3):722–48. doi: 10.1038/s44318-025-00663-2 (PMC12864877; doi:10.1038/s44318-025-00663-2)
Supplement: Supplementary file 1 — Appendix [file 44318_2025_663_MOESM1_ESM.pdf]

Appendix

3′UTR shortening alleviates miRNA repression of mRNAs critical for muscle stem cell differentiation

Yi Zhu[1,7], Jianshu Wang[1,7], Deng Tong[1,7], Peixuan Jia[2,7], Suli Chen[1,2], Yangyang Li[3], Jiaying Fu[2], Qiming Li[3], Ping Hu[4,5,*], Yu Zhou[3,6,*] and Hong Cheng[1,2,*]

Table of contents

Appendix Figure S1 .... page 2

Appendix Figure S2 .... page 3

Appendix Figure S3 .... page 4

Appendix Table S1 .... page 5

Appendix Table S2 .... page 5

Appendix Table S3 .... page 5

Appendix Table S4 .... page 6

Appendix Table S5 .... page 8

Appendix Figure S1

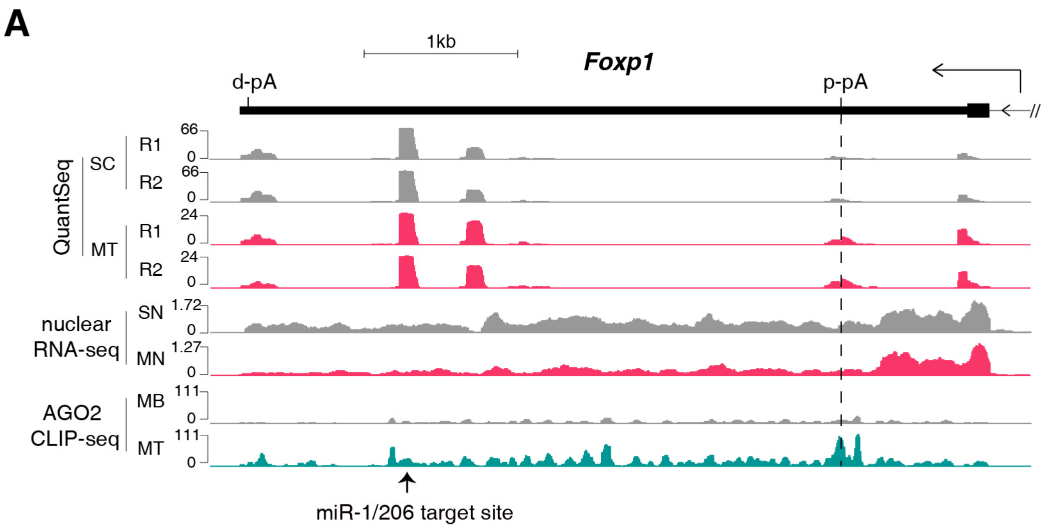

Appendix Figure S1. Predicted miR-1/206 target site in *Foxp1* aUTR.

(A) Screenshot of QuantSeq, nuclear RNA-seq, and AGO2 CLIP-seq signals of *Foxp1* aUTR. Predicted miR-1/206 target site is indicated by an arrow.

Appendix Figure S2

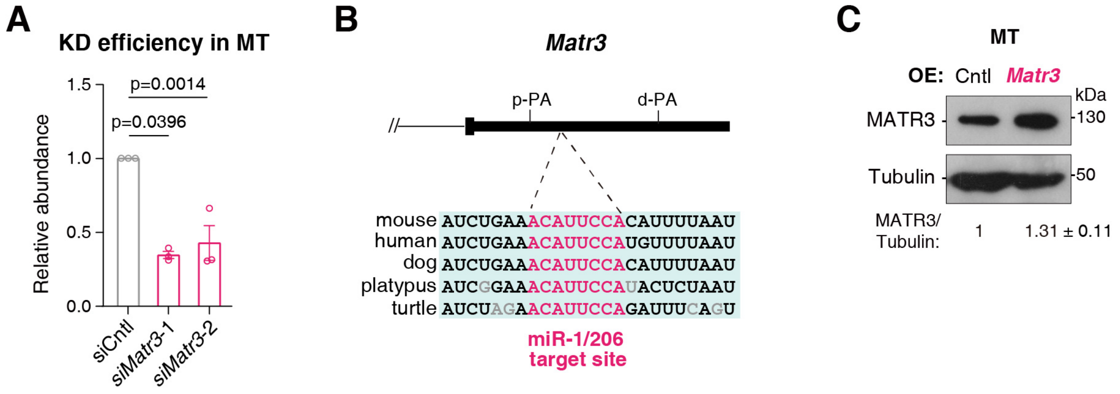

Appendix Figure S2. The APA-miRNA interplay fine-tunes *Matr3* expression for efficient muscle differentiation.

(A) RT-qPCR to detect KD efficiency of *Matr3* in differentiated MTs (n = 3). Bars represent RNA abundance normalized to *Gapdh*. Error bars, mean ± SEM. *P*-values, two-sided unpaired student's t test.

(B) The sequences of the conserved miR-1/206 target site in *Matr3* aUTR are labeled in magenta.

(C) Western blots showing *Matr3* protein levels in MTs differentiated from MBs transfected with Cntl or *Matr3* expression plasmid. Relative protein abundance to Tubulin (mean ± SEM, n = 3) are shown at the bottom.

Appendix Figure S3

A

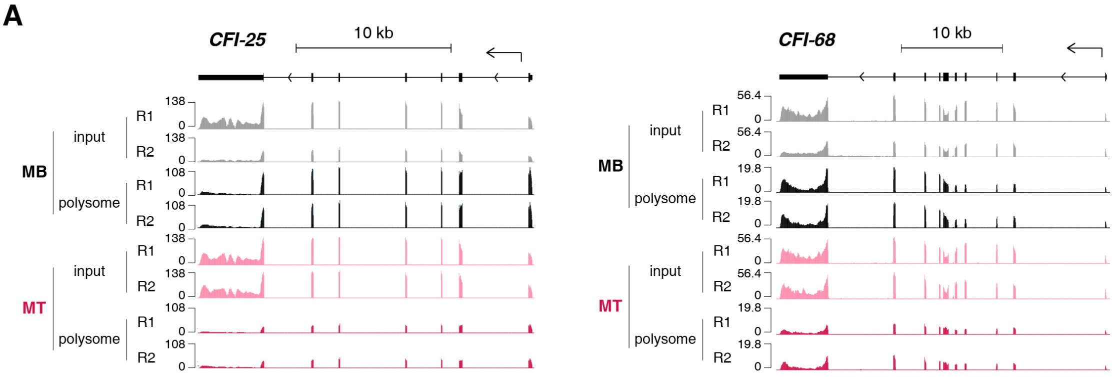

Appendix Figure S3. Reduced polysome association of *CFI-25* and *CFI-68* upon differentiation.

(A) Polysome profiling showing the distribution of *CFI-25* and *CFI-68* in MBs and MTs.

Appendix Table S1. siRNAs.

Target gene	Targeting sequence (5′ to 3′)
Cntl	CGTACGCGGAATACTTCGA
CFI-68	CCTGTTGTAACTCCATGCAAT
Matr3 (1#)	GCTCCTCCAAGTAGCAATA
Matr3 (2#)	GGACAAGATTGAGGAACTT
Pabpc1	CGTGCTTTGGACACCATGAAT
Pabpn1	TAGAGCGACATCATGGTATTC

Appendix Table S2. Antisense morpholino oligonucleotides (AMOs).

Target gene	AMO sequences (5′ to 3′)
Control	CCTCTTACCTCAGTTACAATTTATA
Matr3-p	TTGGTTTTGTTCTTTATTGAATGGA
Matr3-tss	TCAGATTAAAATGTGGAATGTTTCA

Appendix Table S3. Antisense oligonucleotides (ASOs).

Target gene	ASO sequences (5′ to 3′)
Control	CCUUCCCUGAAGGUUCCUCC
Cpd	CGACCCUUUUAUUUUUCACU
Foxp1	UUAAAAAAUUUGGGAUAAUUAUUUUUAA
Il6st	UAGCUUUAUUAGACGGUUUU
Neo1	GGCCUUUAAUAAUUAUAAUA
RalA	UAAAUUAUAUUUAUUAGUAU
Sec63	CAAAGGAUUUAUUAUCAUUU

ASOs were engineered with full substitution of 2′-O-methoxyethyl oligonucleotides and a phosphorothioate backbone.

Appendix Table S4. Primers.

Primer Name	Primer Sequences
Isoform-specific (RT)	CAAGCAGAAGACGGCATACGAGATTTT TTTTTTTTTTTTTTTTTTTTTVN
CFI-25 Forward	CATTTCTAGTCTTCCTCAGCTGCTG
CFI-25 Reverse	TGTAAGCTGTGCTCATAGAGACGG
CFI-68 Forward	CTGACGCTAGTGCTGGTGAT
CFI-68 Reverse	TTGATCGTTCACGTCTTCTGGAT
Cpd-S Forward	TCAGTGTGCTTGGCTGGTT
Cpd-L Forward	ACATGTCATGGTGTCCTGC
Cpd-L Reverse	GTTATCAACAAGTTGCTAGACAG
Foxp1-S Forward	GCACAGCGCTGCATGTTGATA
Foxp1-L Forward	AGCACCCACCATTGTGAGTA
Foxp1-L Reverse	TGGTCCTGGTCACCTGATTAT
Gapdh Forward	ACCCAGAAGACTGTGGATGG
Gapdh Reverse	ACACATTGGGGGTAGGAACA
Il6st-S Forward	GGAGCACCTCCCTTTGCTCT
Il6st-L Forward	TGTGGTCATCAGCTCGGC
Il6st-L Reverse	CAGTCTCACCAGACAGTGGT
Luc-S Forward	GAGCTCGCTAGCCTCGAG
Luc-L Forward	GAGCTCGCTAGCCTCGAG
Luc-L Reverse	TCAAATGAAAAGTCTGCAAGGC
Matr3-S Forward	ATACGGTTAAGTTAATGAAAATGTT
Matr3-L Forward	CAGCAGCCTTCCTCATTATCA
Matr3-L Reverse	AAATGTGGAATGTTTCAGATG
Matr3 Forward	GACTCTTACCGAAGGGTTATC

Matr3 Reverse	CCATTCTGGGTAGATTTCAA
Myh1 Forward	CTCTTCCCGCTTTGGTAAGTT
Myh1 Reverse	CAGGAGCATTTCGATTAGATCCG
Myh3 Forward	ATGCCACCTTCGCTACAACA
Myh3 Reverse	GTTCAGCACTCGGTATCTCTGT
Mck Forward	CACCTCCACAGCACAGACAG
Mck Reverse	ACCTTGGCCATGTGATTGTT
Neo1-S Forward	CTGAGACATTGCATCCTCTGC
Neo1-L Forward	ACCCATGACGCTTCCCCA
Neo1-L Reverse	GTCTTCTGCGTGTGGCTGAA
RalA-S Forward	GAGGGAAATACGAGCCAGAAAGA
RalA-L Forward	AGATTATGAGCGTGAGTACCAA
RalA-L Reverse	CAGACCACATTAGCTCTCCTC
Sec63-S Forward	GGTGGTGCGTTTATCTCATCTG
Sec63-L Forward	GCTCTCAGTAAAAGTCAAGGC
Sec63-L Reverse	GTGAGAATGCTAAGTTATGGCC
Pabpc1 Forward	GCCACTGGTGTTCCAACTGTCTA
Pabpc1 Reverse	TTAAGATATTTTTCTTCGGTGAAGCA
Pabpn1 Forward	CTACAGTGGTTTTAACAGCAGGCC
Pabpn1 Reverse	TTCCATCAAGGTCATCTTCTGTTTT
RS	CAAGCAGAAGACGGCATACGAGA
Matr3$^{mpA/mpA}$ Forward (genotyping)	TGTGTCCTGAGCCAGTTTAGTTTTGT
Matr3$^{mpA/mpA}$ Reverse (genotyping)	AGCTACAGGATTCAAGTGACAGGCA

Appendix Table S5. Candidate myomiR target sequences.

myomiRs	targeting sequence (5′ to 3′)
mmu-miR-1	ACATTCC
mmu-miR-133	GGACCAA
mmu-miR-206	ACATTCC
mmu-miR-208	AAAAGCT
mmu-miR-486	GTACAGG
mmu-miR-499	AGTCTTA
