# Supplementary figures and images for: 3′UTR shortening alleviates miRNA repression of mRNAs critical for muscle stem cell differentiation
Source: EMBO J. 2025 Dec 22;45(3):722–48. doi: 10.1038/s44318-025-00663-2 (PMC12864877; doi:10.1038/s44318-025-00663-2)

Source data for **Fig 2B**

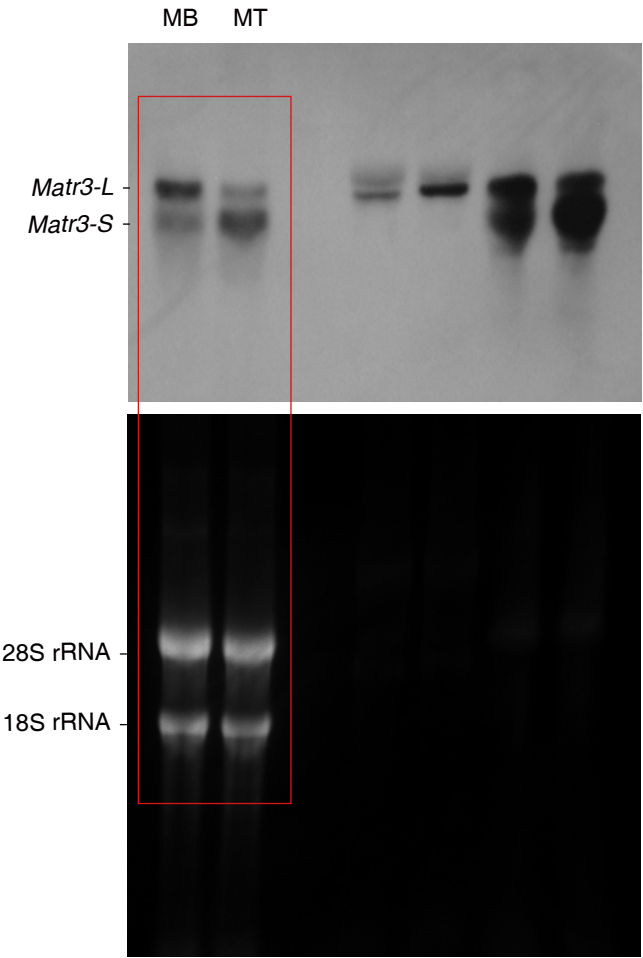

Supplement: Supplementary file 7 — Source data Fig. 2 [file 44318_2025_663_MOESM7_ESM.zip › EMBOJ-2025-121889_SourceDataForFigure2/2B/Northern blot for 2B.pdf]

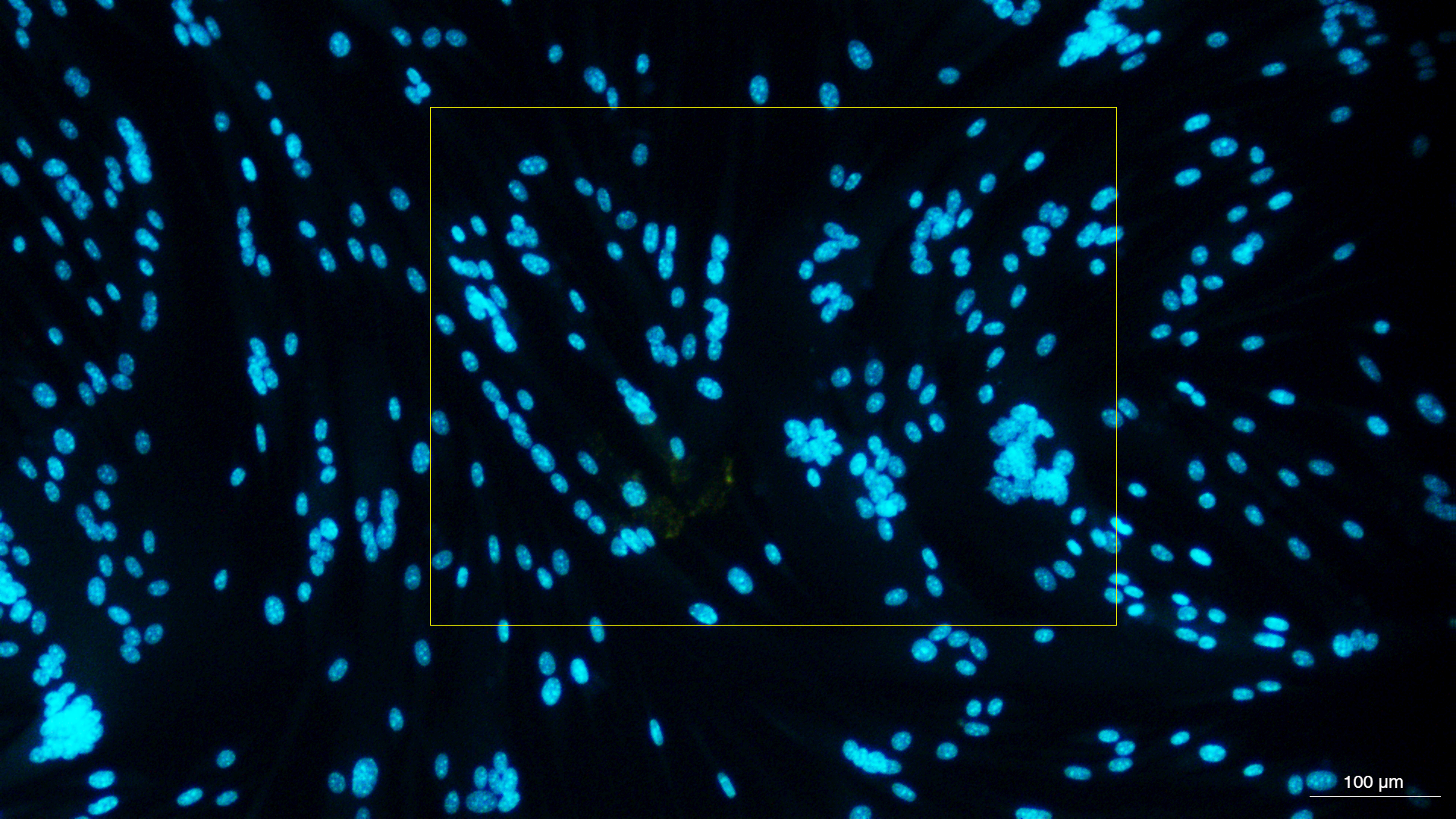

Supplement: Supplementary file 7 — Source data Fig. 2 [file 44318_2025_663_MOESM7_ESM.zip › EMBOJ-2025-121889_SourceDataForFigure2/2E/Cntl AMO/DAPI.tif]

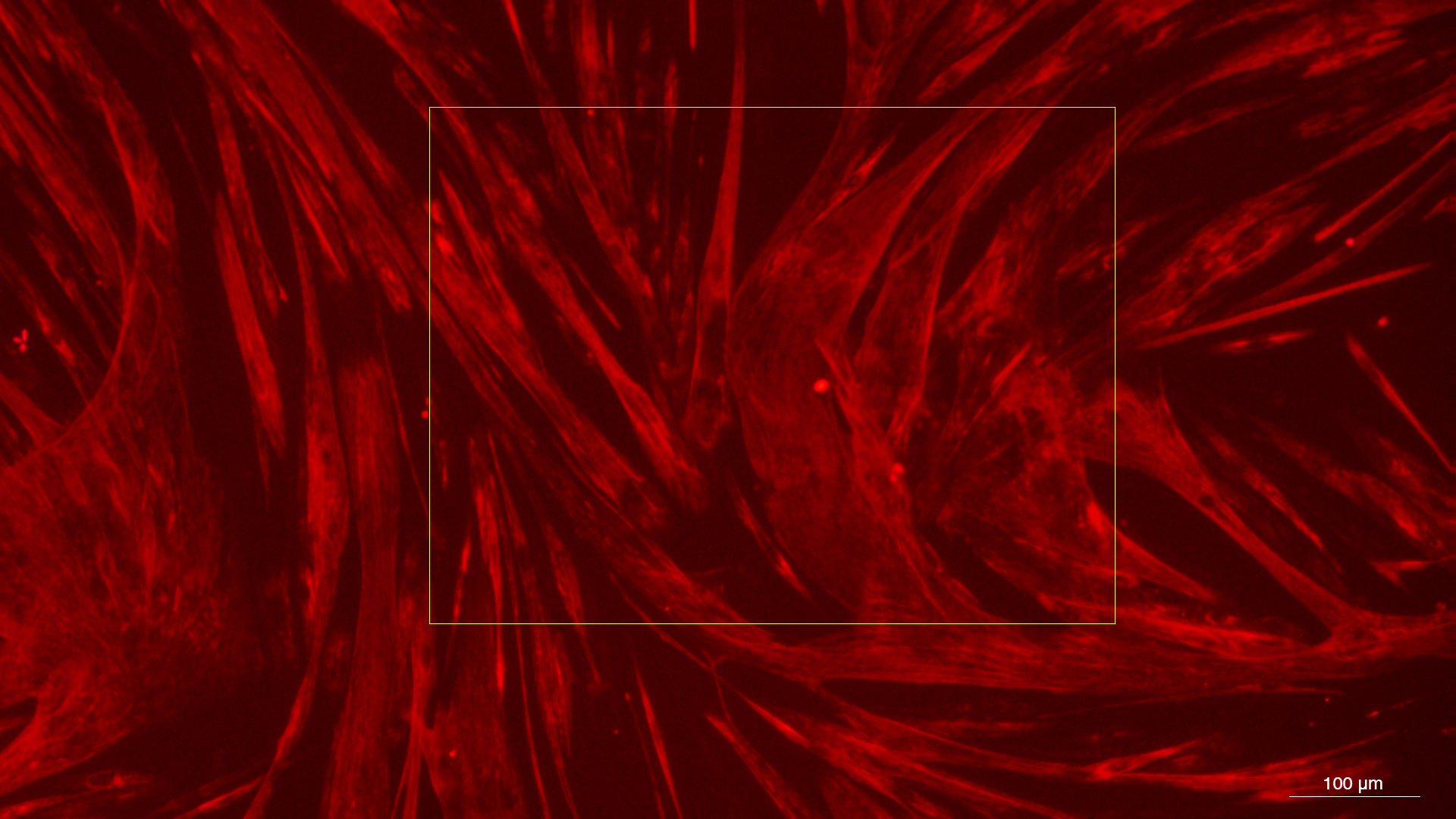

Supplement: Supplementary file 7 — Source data Fig. 2 [file 44318_2025_663_MOESM7_ESM.zip › EMBOJ-2025-121889_SourceDataForFigure2/2E/Cntl AMO/MyHC.tif]

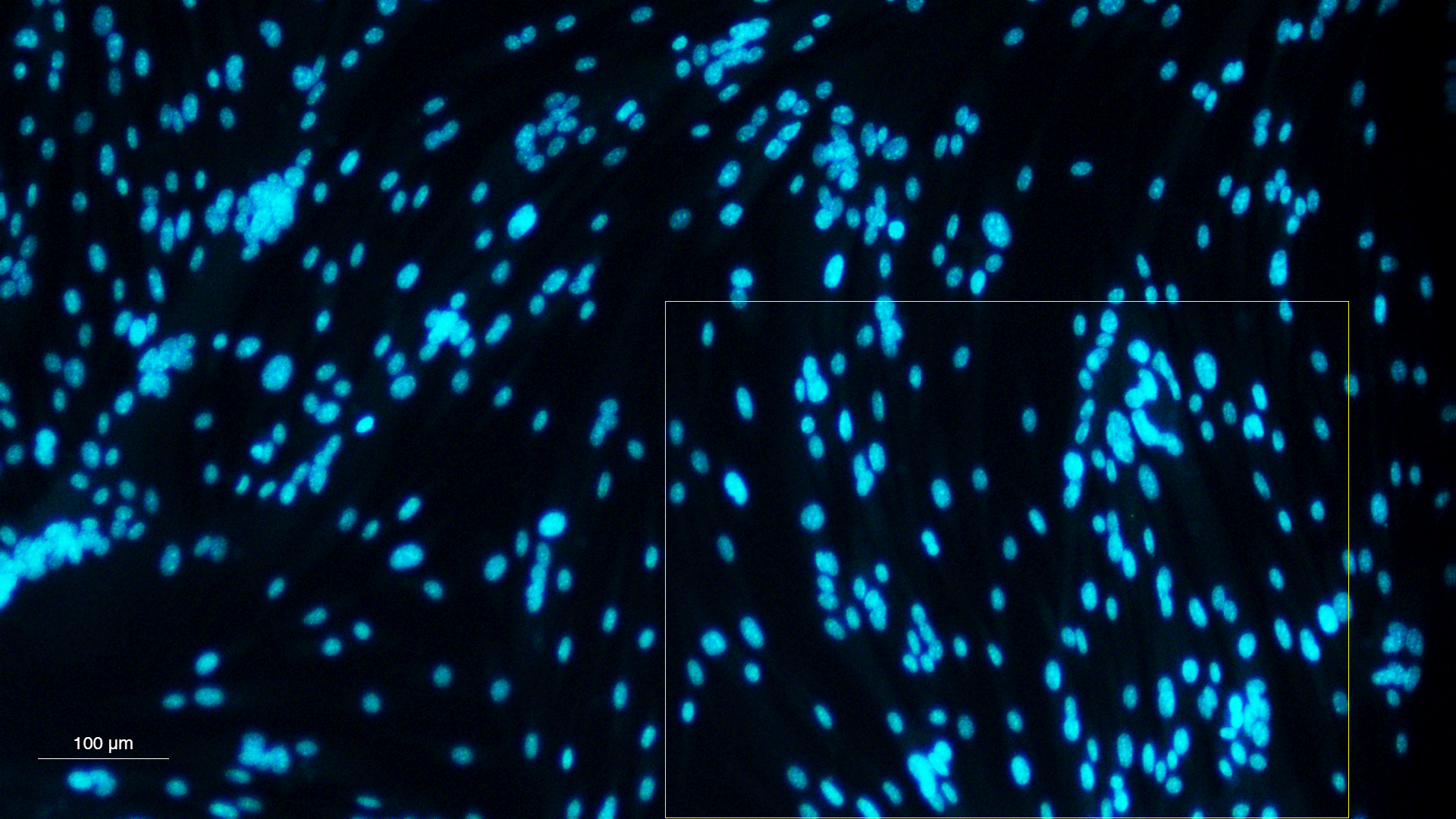

Supplement: Supplementary file 7 — Source data Fig. 2 [file 44318_2025_663_MOESM7_ESM.zip › EMBOJ-2025-121889_SourceDataForFigure2/2E/Matr3-pAMO/DAPI.tif]

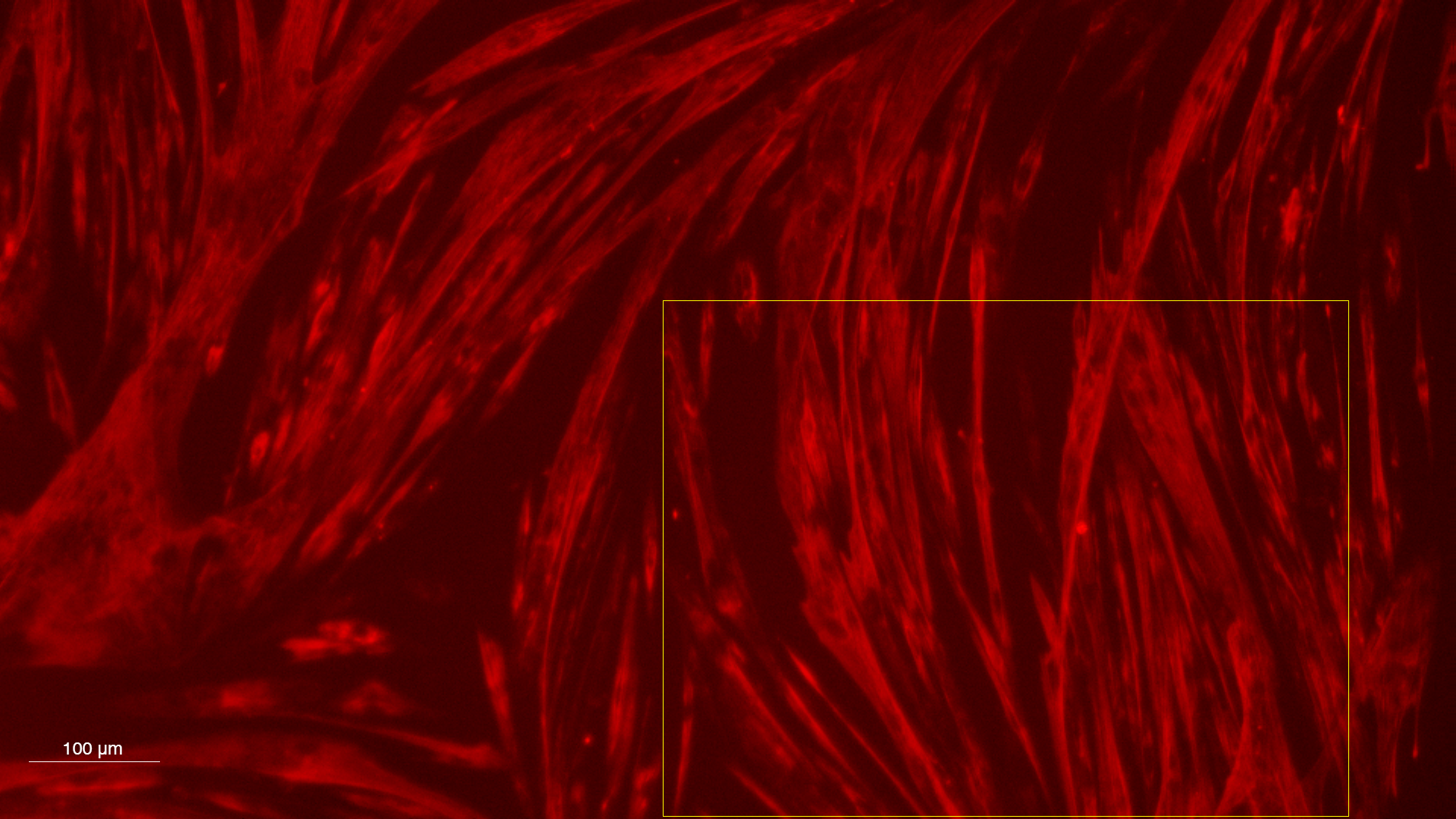

Supplement: Supplementary file 7 — Source data Fig. 2 [file 44318_2025_663_MOESM7_ESM.zip › EMBOJ-2025-121889_SourceDataForFigure2/2E/Matr3-pAMO/MyHC.tif]

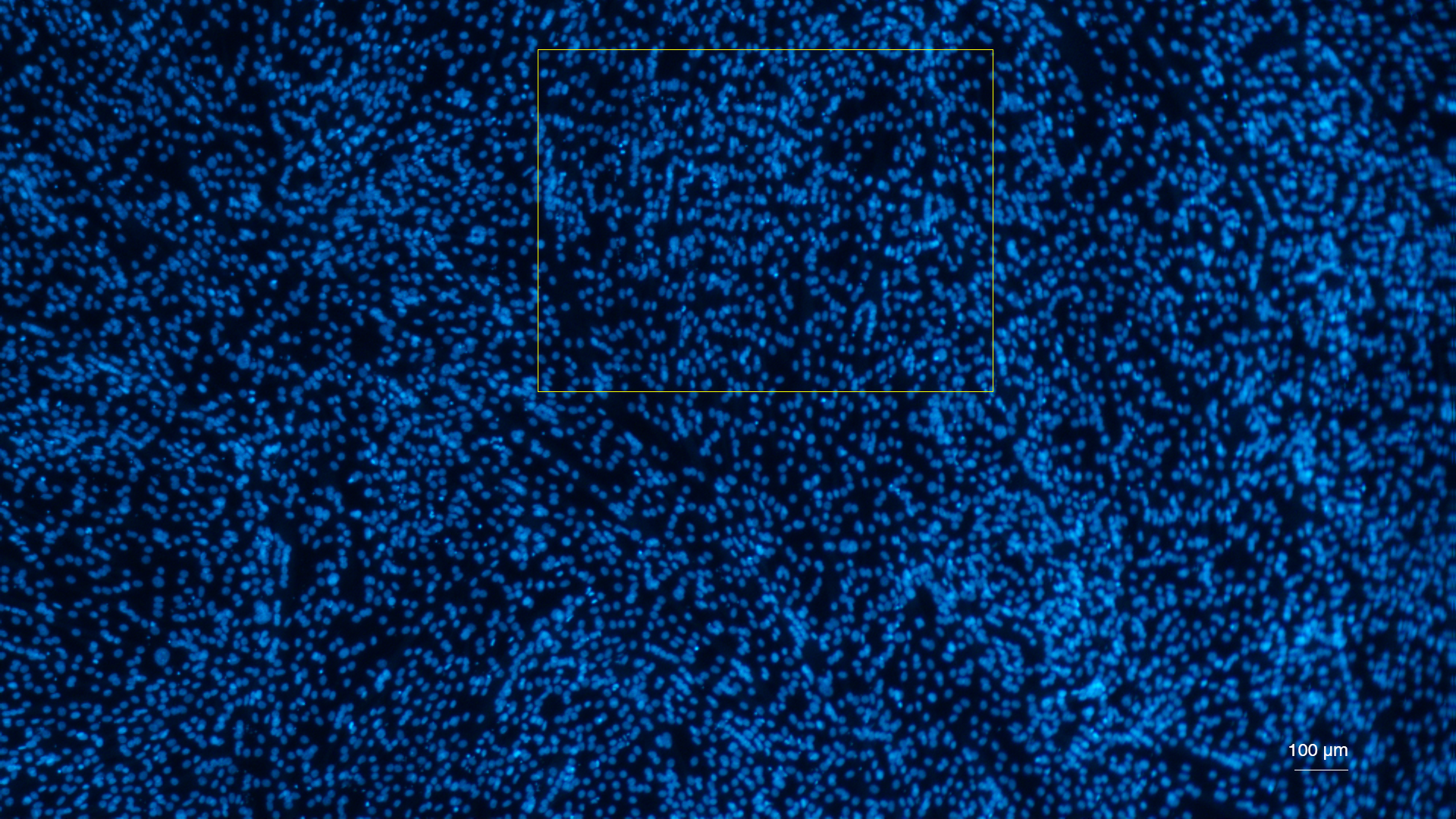

Supplement: Supplementary file 7 — Source data Fig. 2 [file 44318_2025_663_MOESM7_ESM.zip › EMBOJ-2025-121889_SourceDataForFigure2/2H/Cntl ASO/DAPI.tif]

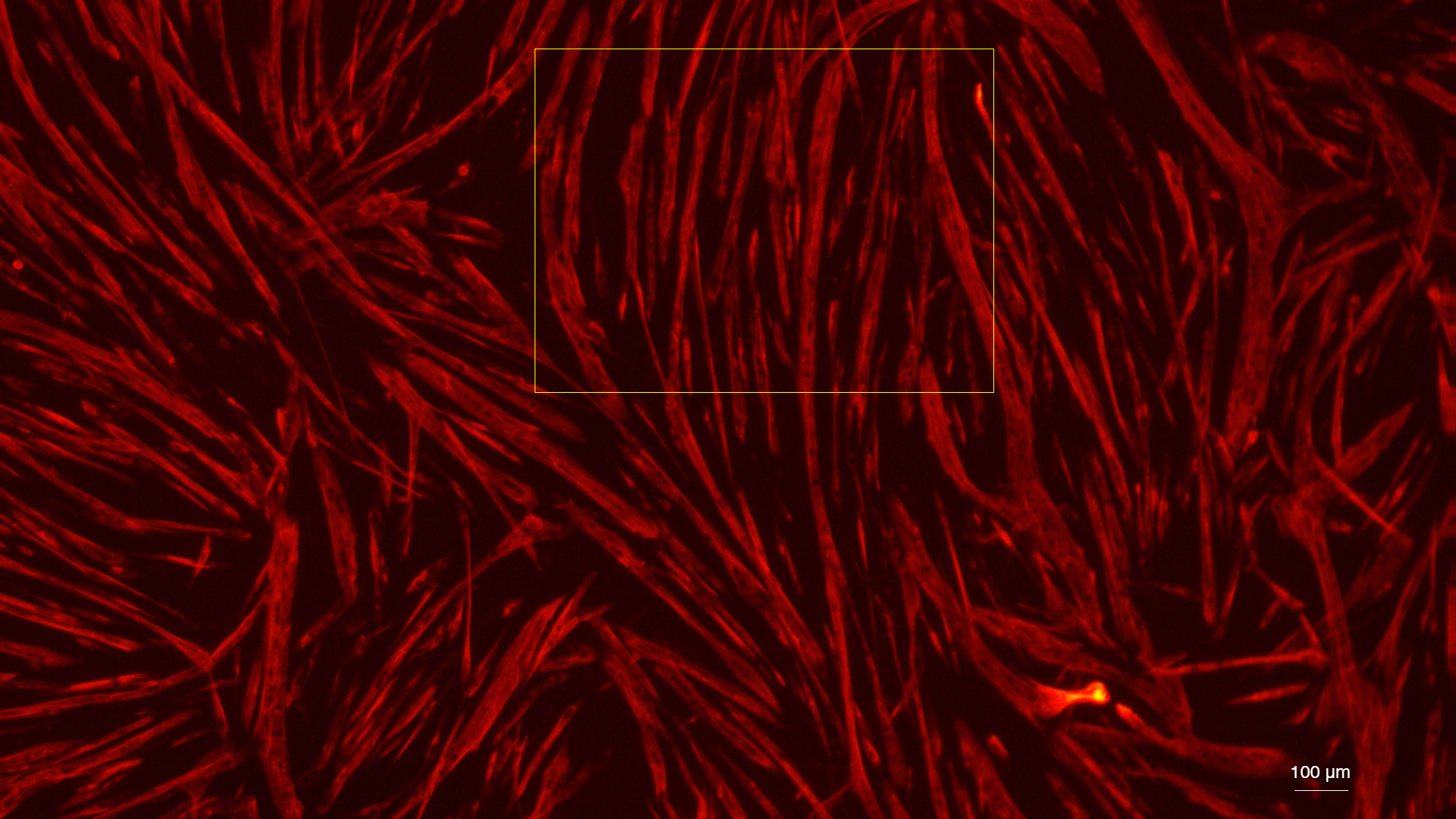

Supplement: Supplementary file 7 — Source data Fig. 2 [file 44318_2025_663_MOESM7_ESM.zip › EMBOJ-2025-121889_SourceDataForFigure2/2H/Cntl ASO/MyHC.tif]

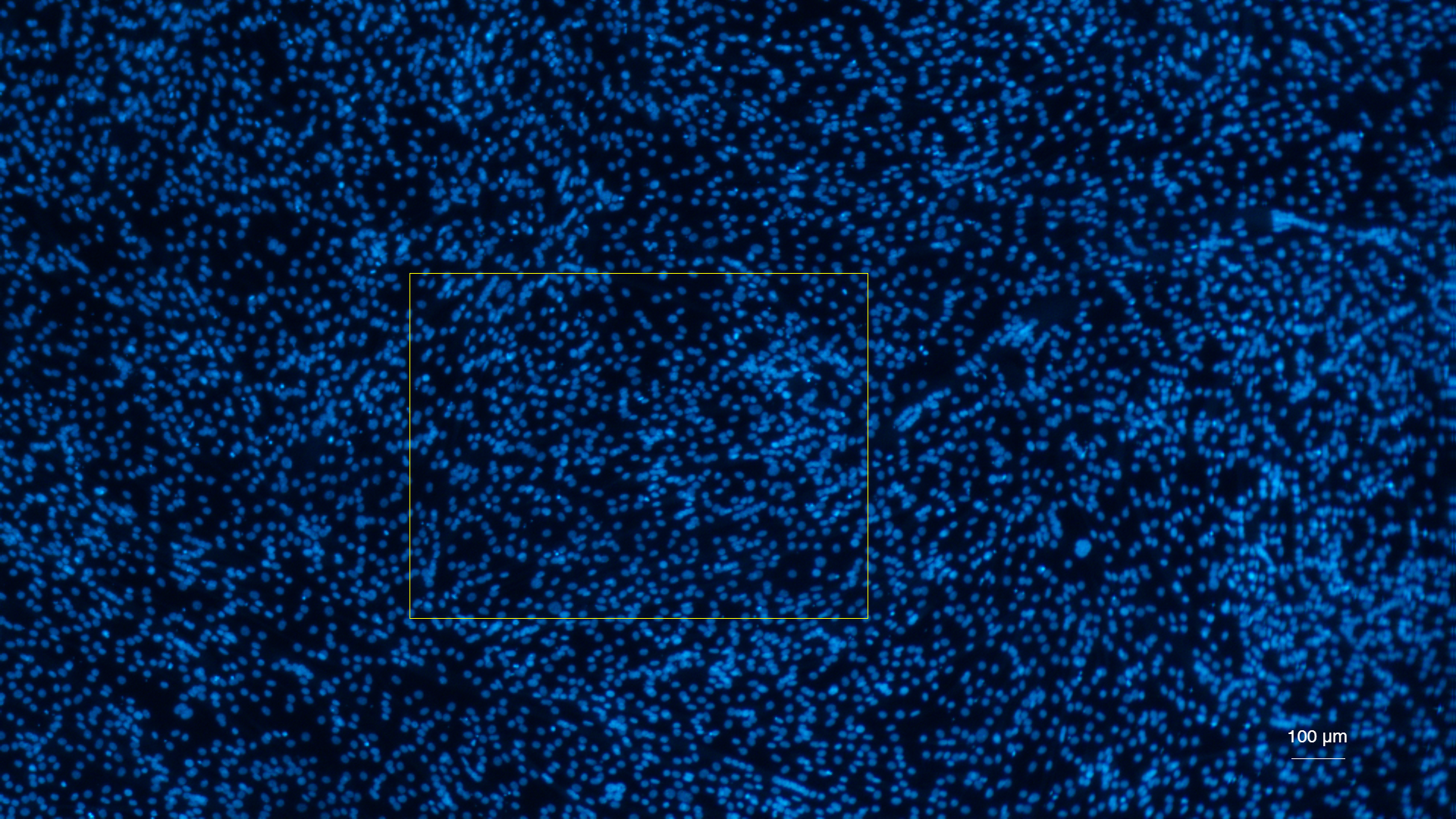

Supplement: Supplementary file 7 — Source data Fig. 2 [file 44318_2025_663_MOESM7_ESM.zip › EMBOJ-2025-121889_SourceDataForFigure2/2H/Foxp1-pASO/DAPI.tif]

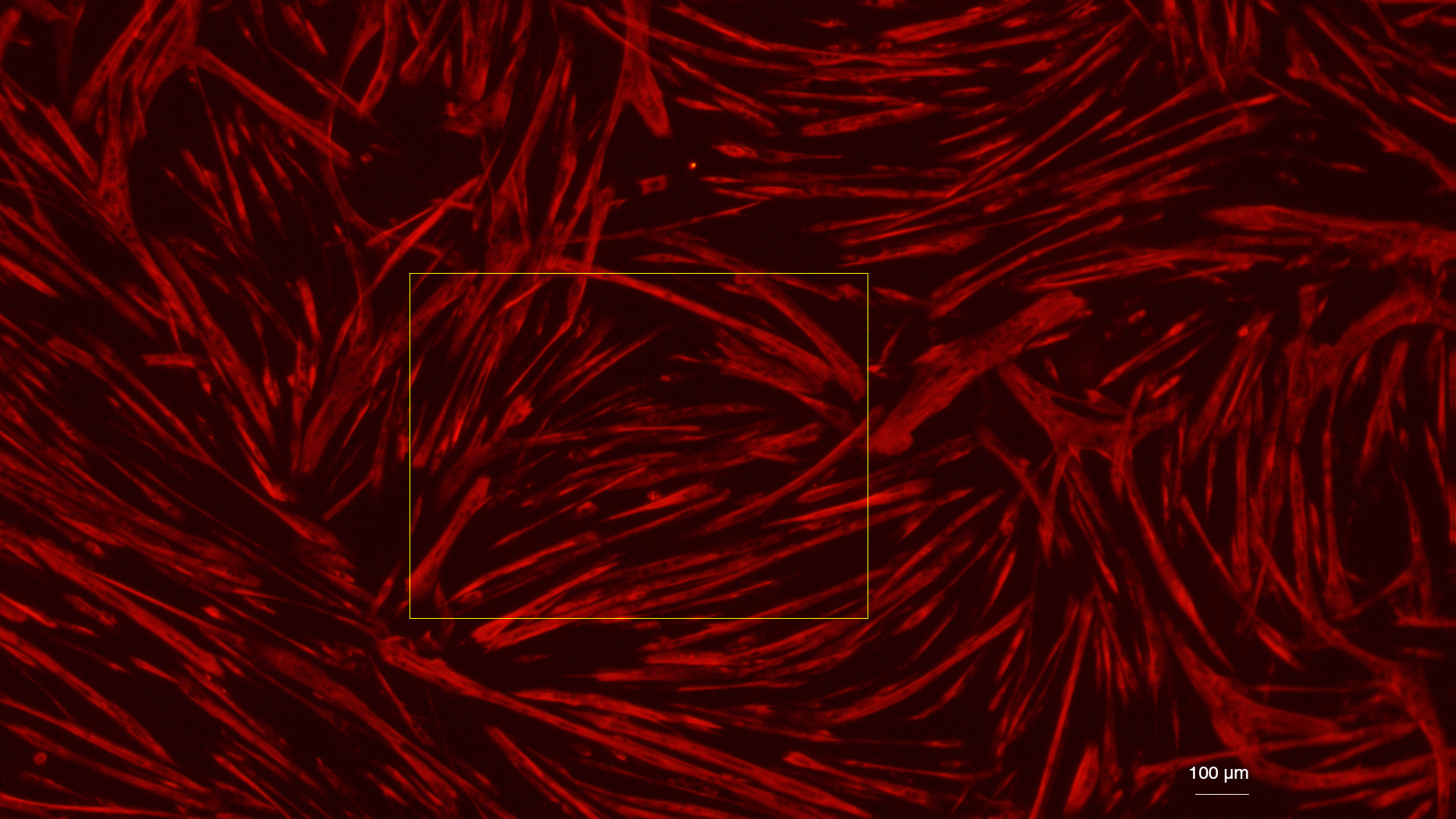

Supplement: Supplementary file 7 — Source data Fig. 2 [file 44318_2025_663_MOESM7_ESM.zip › EMBOJ-2025-121889_SourceDataForFigure2/2H/Foxp1-pASO/MyHC.tif]

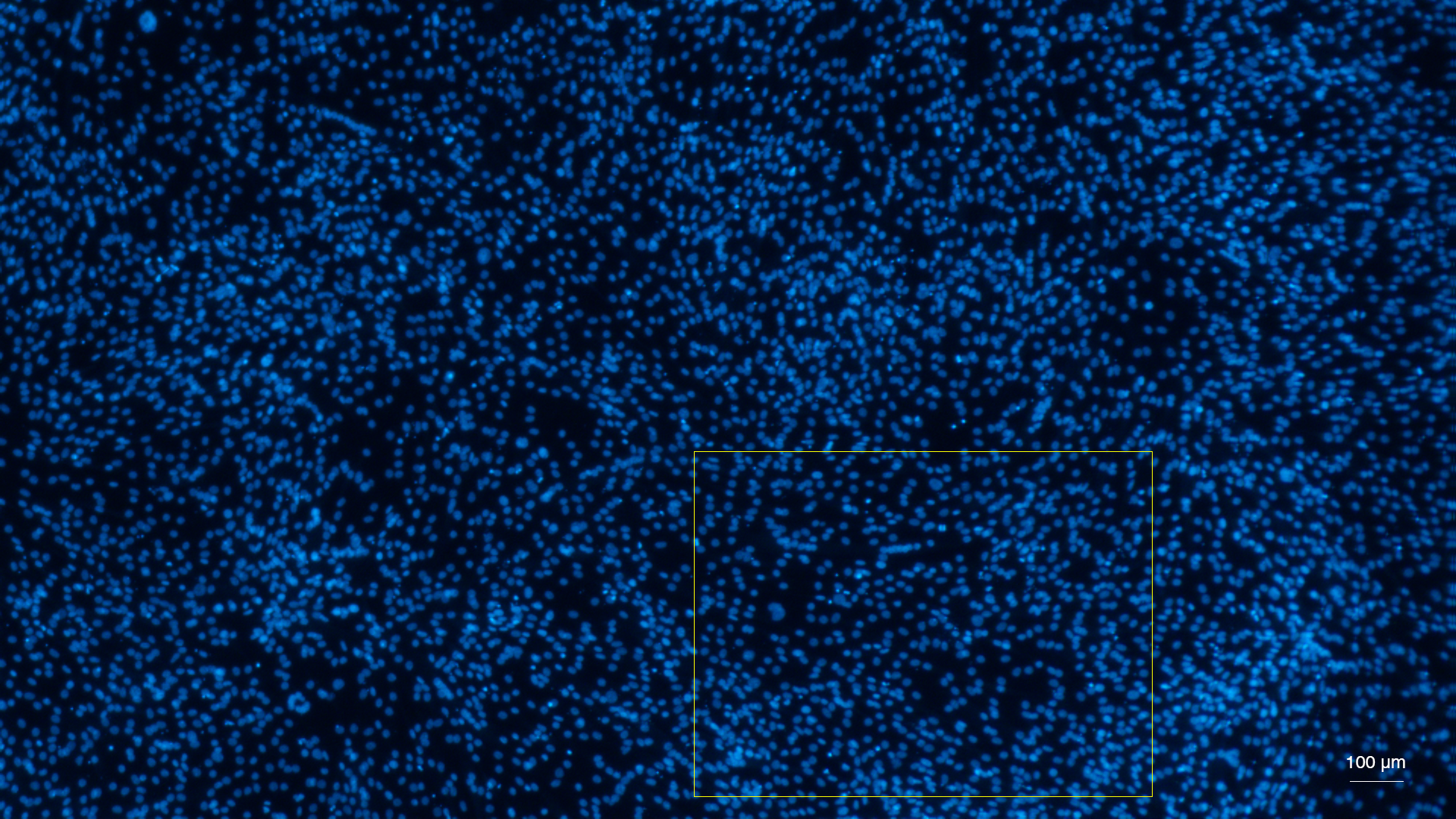

Supplement: Supplementary file 7 — Source data Fig. 2 [file 44318_2025_663_MOESM7_ESM.zip › EMBOJ-2025-121889_SourceDataForFigure2/2H/Neo1-pASO/DAPI.tif]

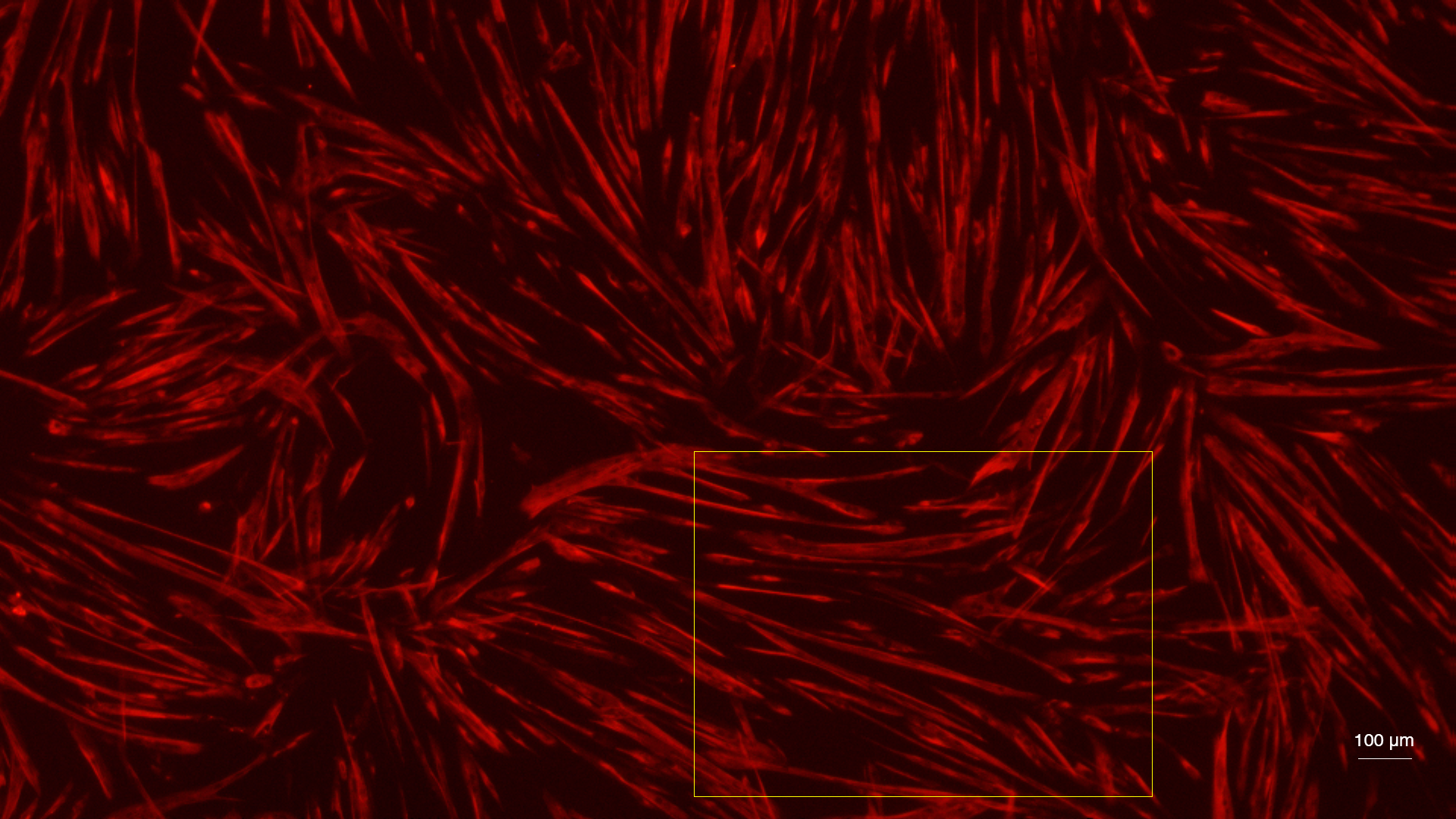

Supplement: Supplementary file 7 — Source data Fig. 2 [file 44318_2025_663_MOESM7_ESM.zip › EMBOJ-2025-121889_SourceDataForFigure2/2H/Neo1-pASO/MyHC.tif]

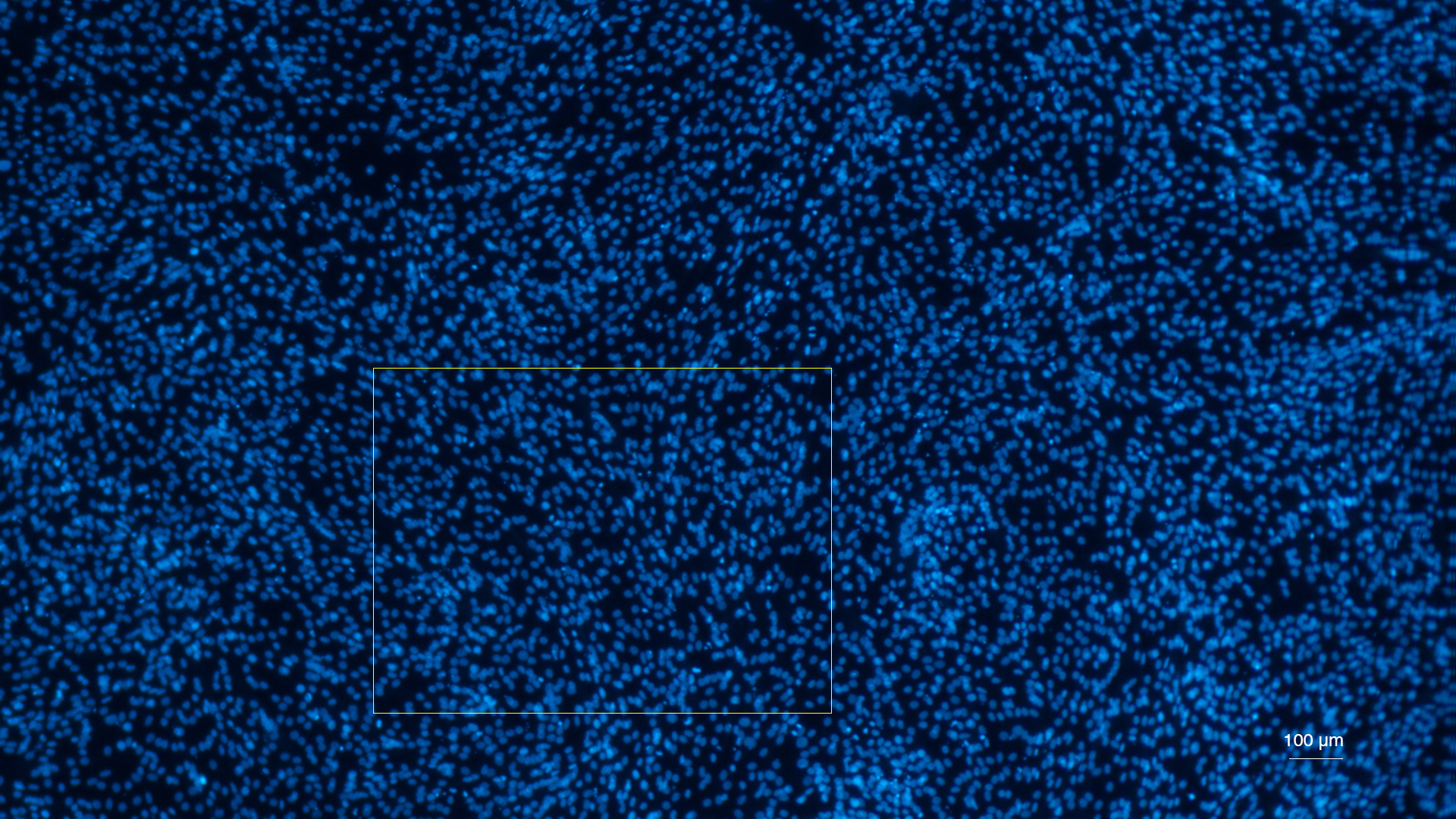

Supplement: Supplementary file 7 — Source data Fig. 2 [file 44318_2025_663_MOESM7_ESM.zip › EMBOJ-2025-121889_SourceDataForFigure2/2H/RalA-pASO/DAPI.tif]

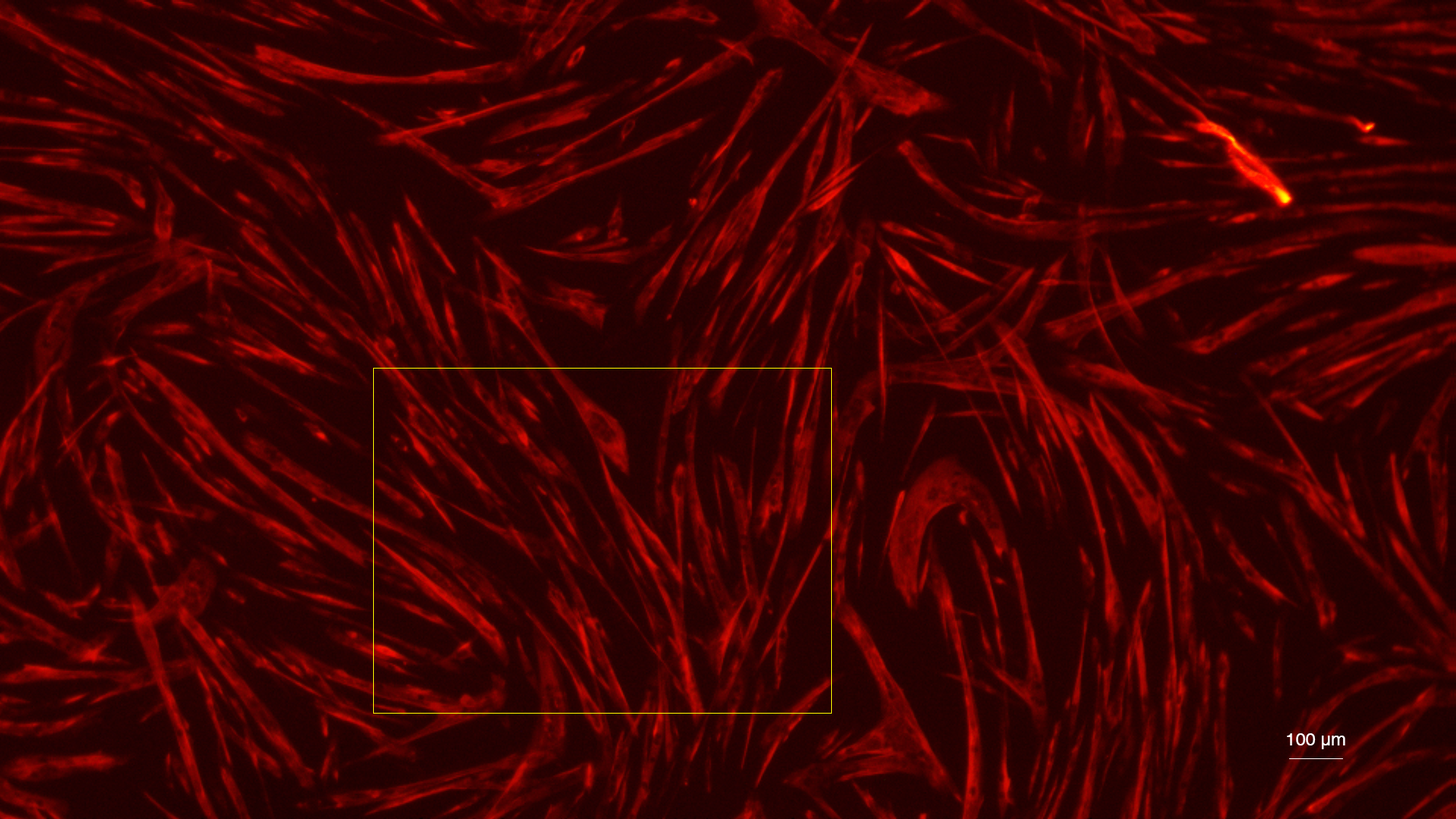

Supplement: Supplementary file 7 — Source data Fig. 2 [file 44318_2025_663_MOESM7_ESM.zip › EMBOJ-2025-121889_SourceDataForFigure2/2H/RalA-pASO/MyHC.tif]

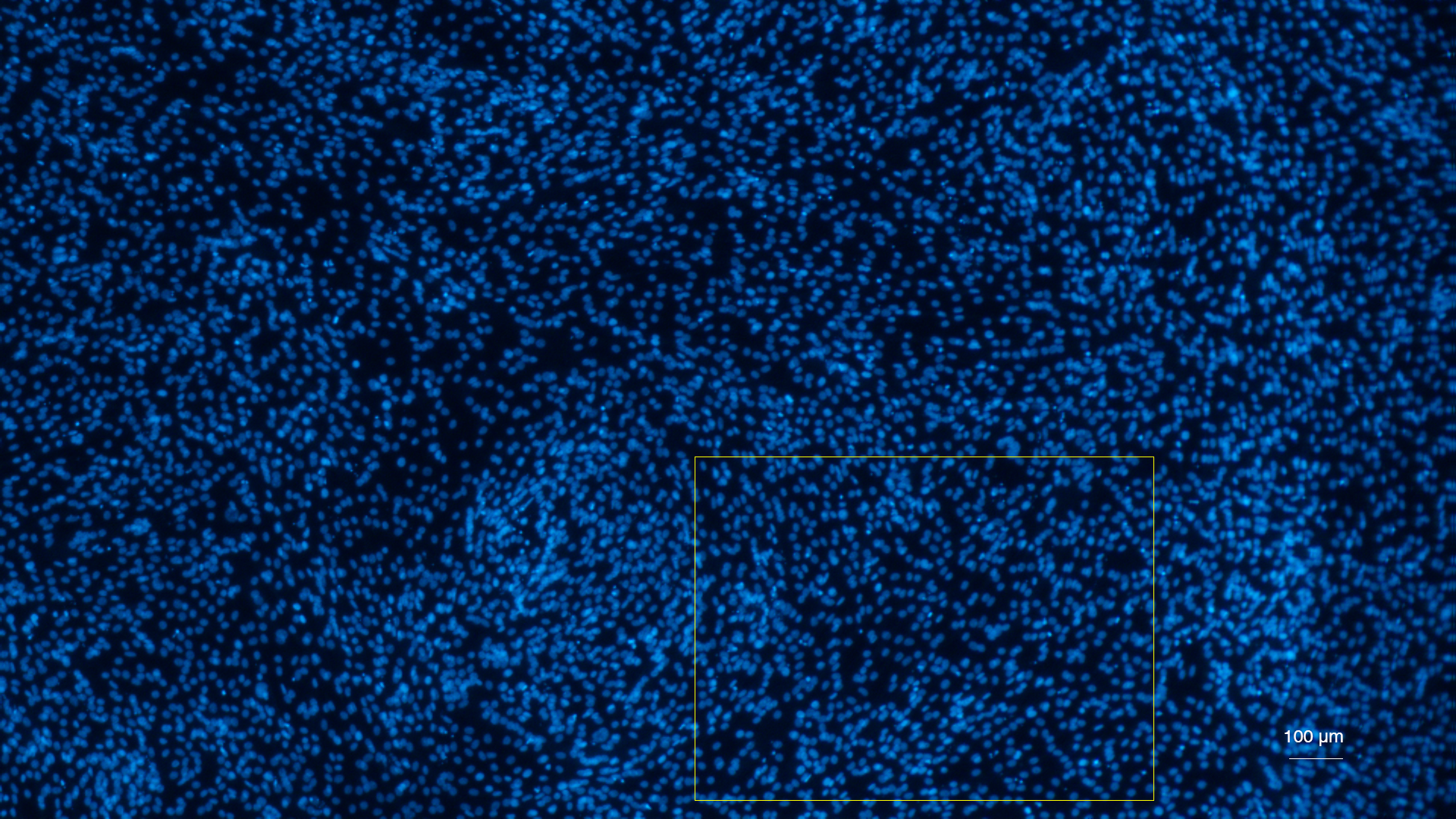

Supplement: Supplementary file 7 — Source data Fig. 2 [file 44318_2025_663_MOESM7_ESM.zip › EMBOJ-2025-121889_SourceDataForFigure2/2H/Sec63-pASO/DAPI.tif]

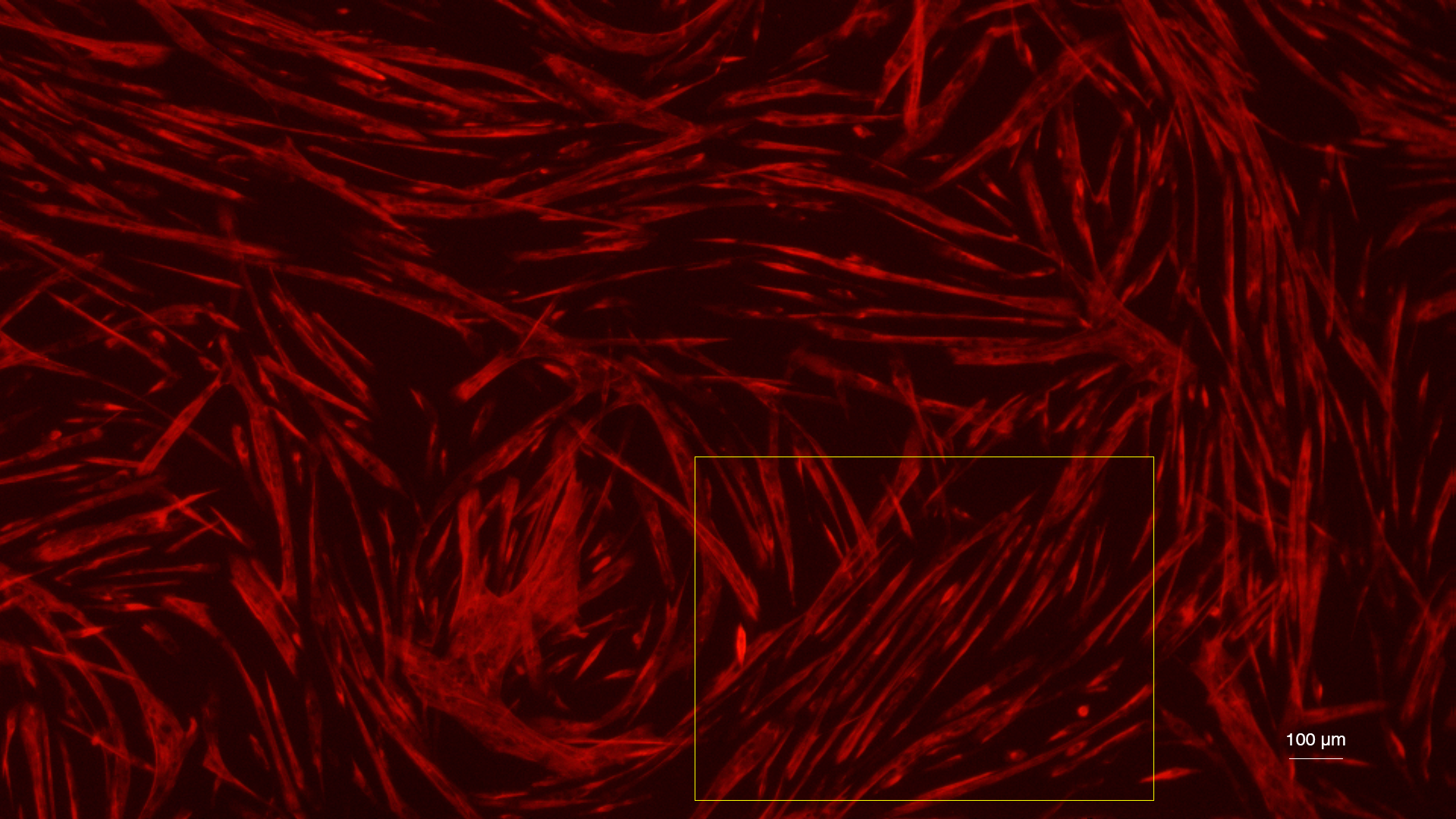

Supplement: Supplementary file 7 — Source data Fig. 2 [file 44318_2025_663_MOESM7_ESM.zip › EMBOJ-2025-121889_SourceDataForFigure2/2H/Sec63-pASO/MyHC.tif]

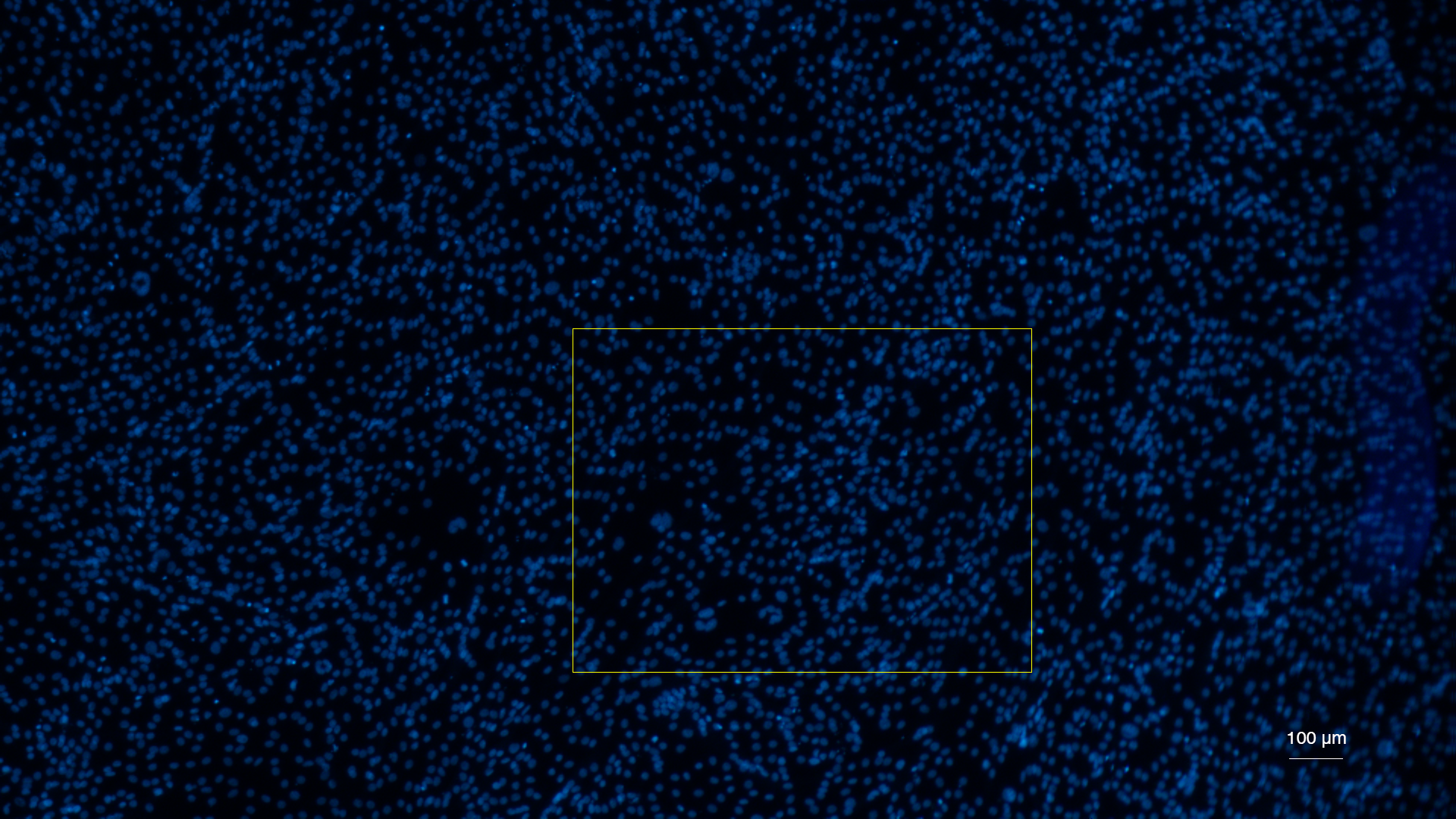

Supplement: Supplementary file 7 — Source data Fig. 2 [file 44318_2025_663_MOESM7_ESM.zip › EMBOJ-2025-121889_SourceDataForFigure2/2I/Cntl ASO/DAPI.tif]

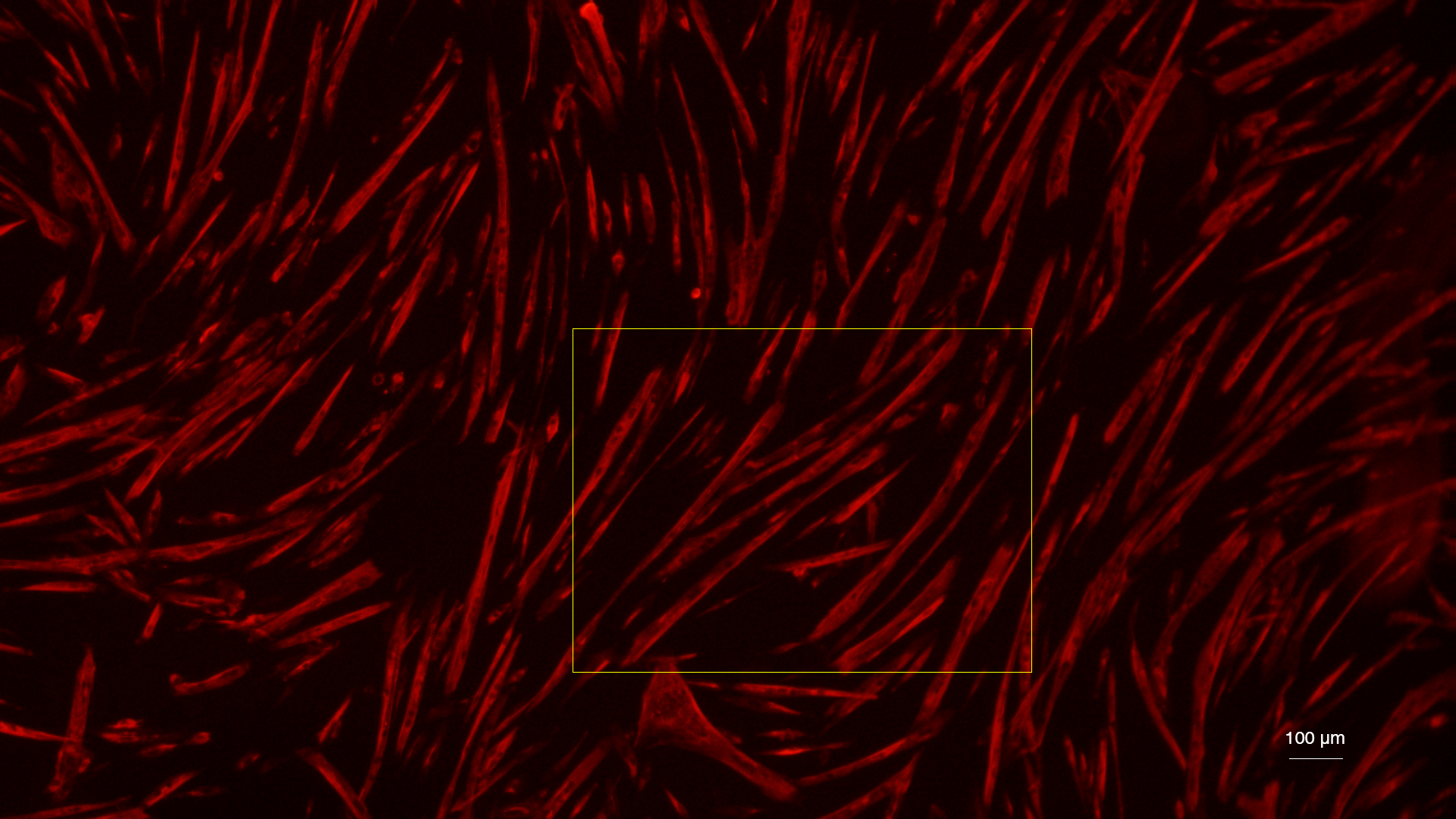

Supplement: Supplementary file 7 — Source data Fig. 2 [file 44318_2025_663_MOESM7_ESM.zip › EMBOJ-2025-121889_SourceDataForFigure2/2I/Cntl ASO/MyHC.tif]

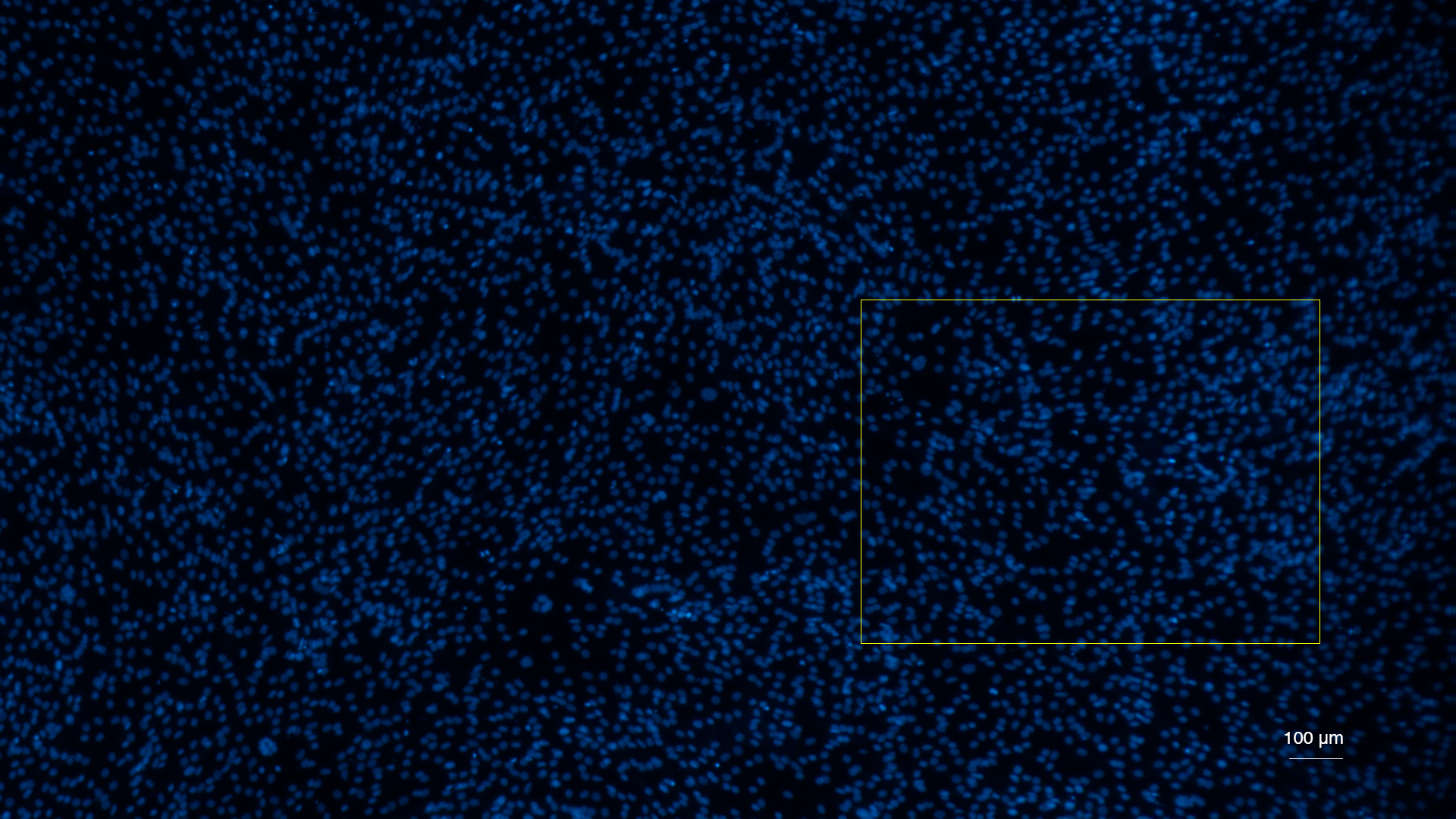

Supplement: Supplementary file 7 — Source data Fig. 2 [file 44318_2025_663_MOESM7_ESM.zip › EMBOJ-2025-121889_SourceDataForFigure2/2I/Cpd-pASO/DAPI.tif]

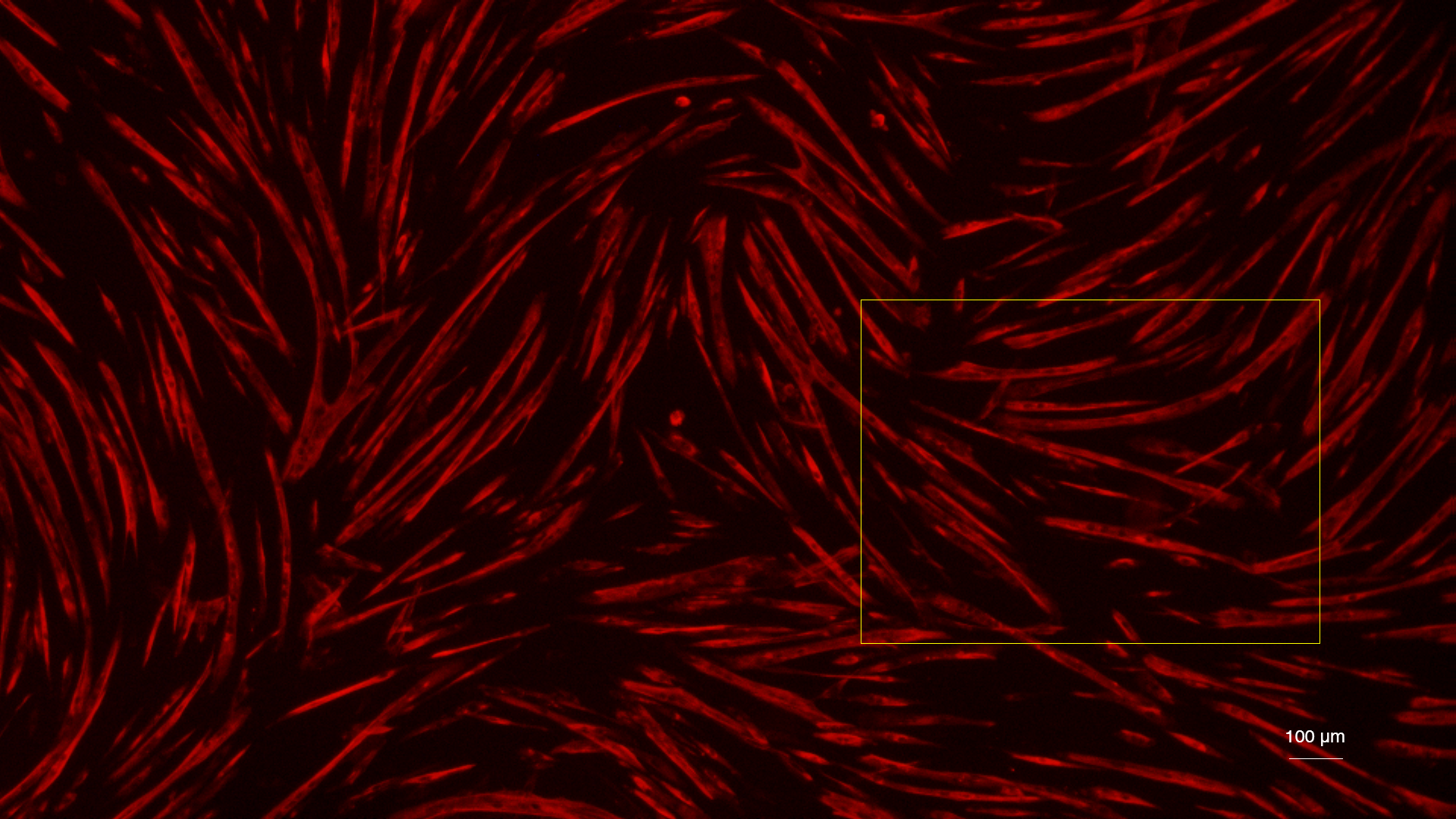

Supplement: Supplementary file 7 — Source data Fig. 2 [file 44318_2025_663_MOESM7_ESM.zip › EMBOJ-2025-121889_SourceDataForFigure2/2I/Cpd-pASO/MyHC.tif]

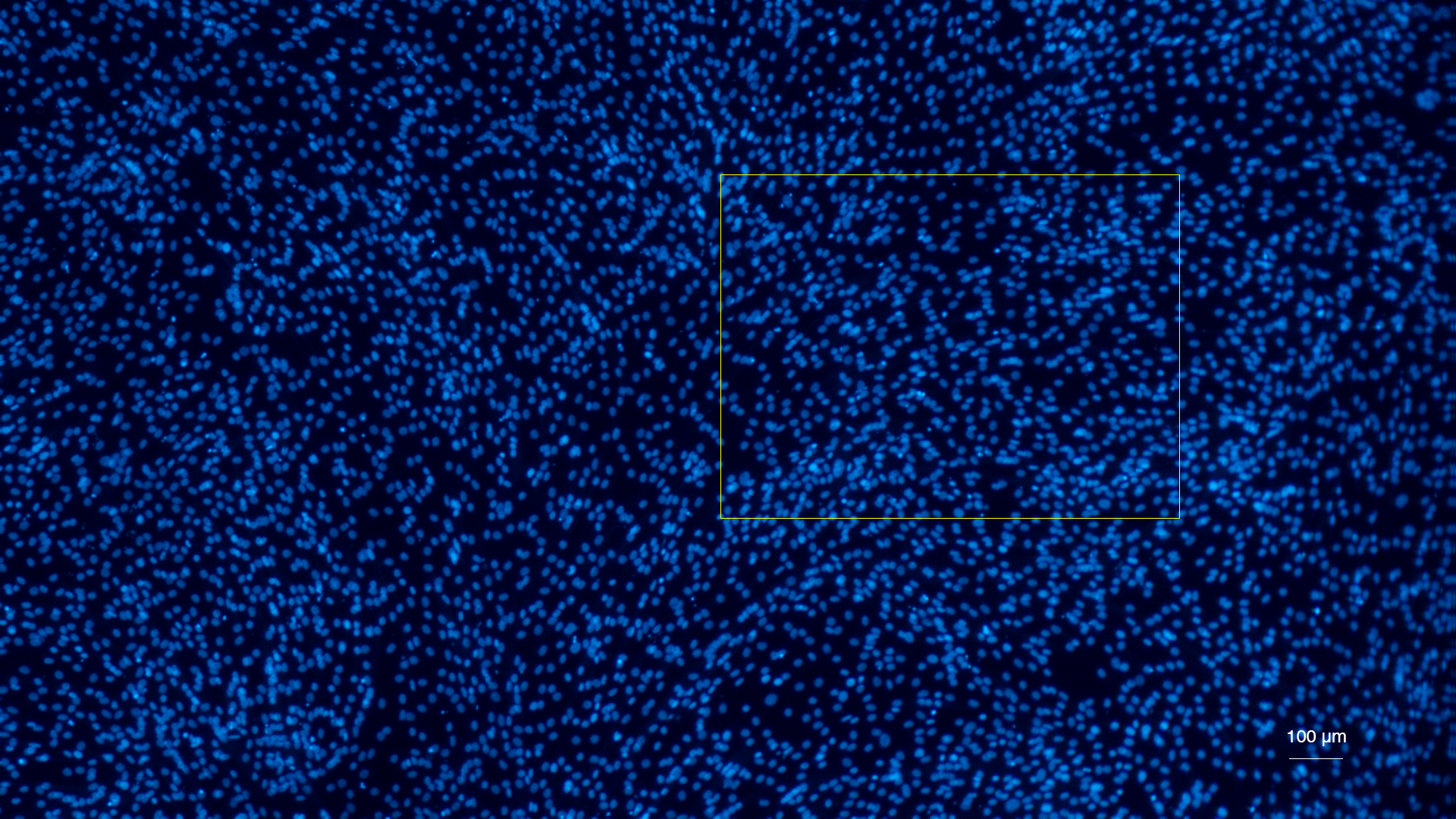

Supplement: Supplementary file 7 — Source data Fig. 2 [file 44318_2025_663_MOESM7_ESM.zip › EMBOJ-2025-121889_SourceDataForFigure2/2J/Cntl ASO/DAPI.tif]

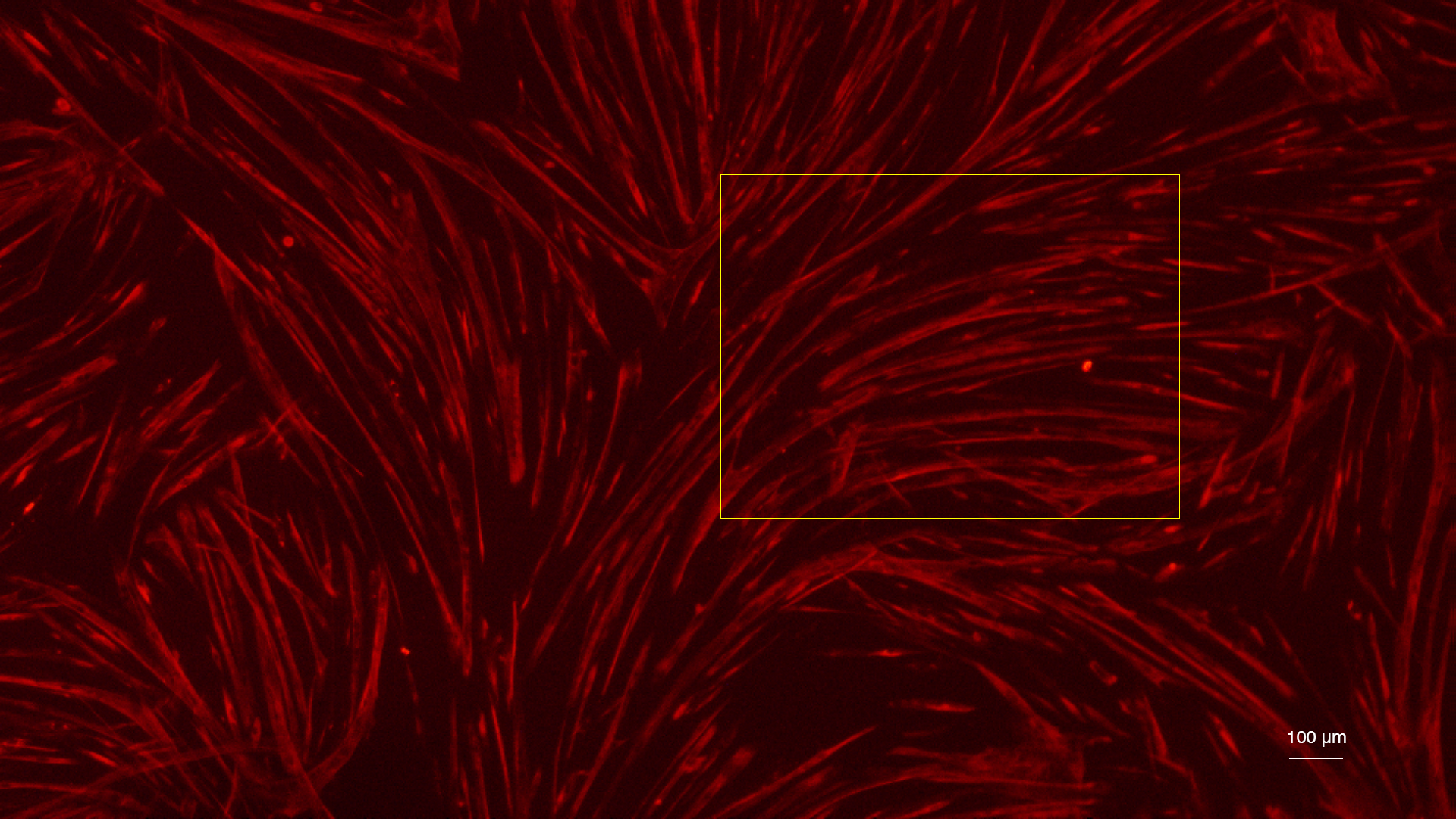

Supplement: Supplementary file 7 — Source data Fig. 2 [file 44318_2025_663_MOESM7_ESM.zip › EMBOJ-2025-121889_SourceDataForFigure2/2J/Cntl ASO/MyHC.tif]

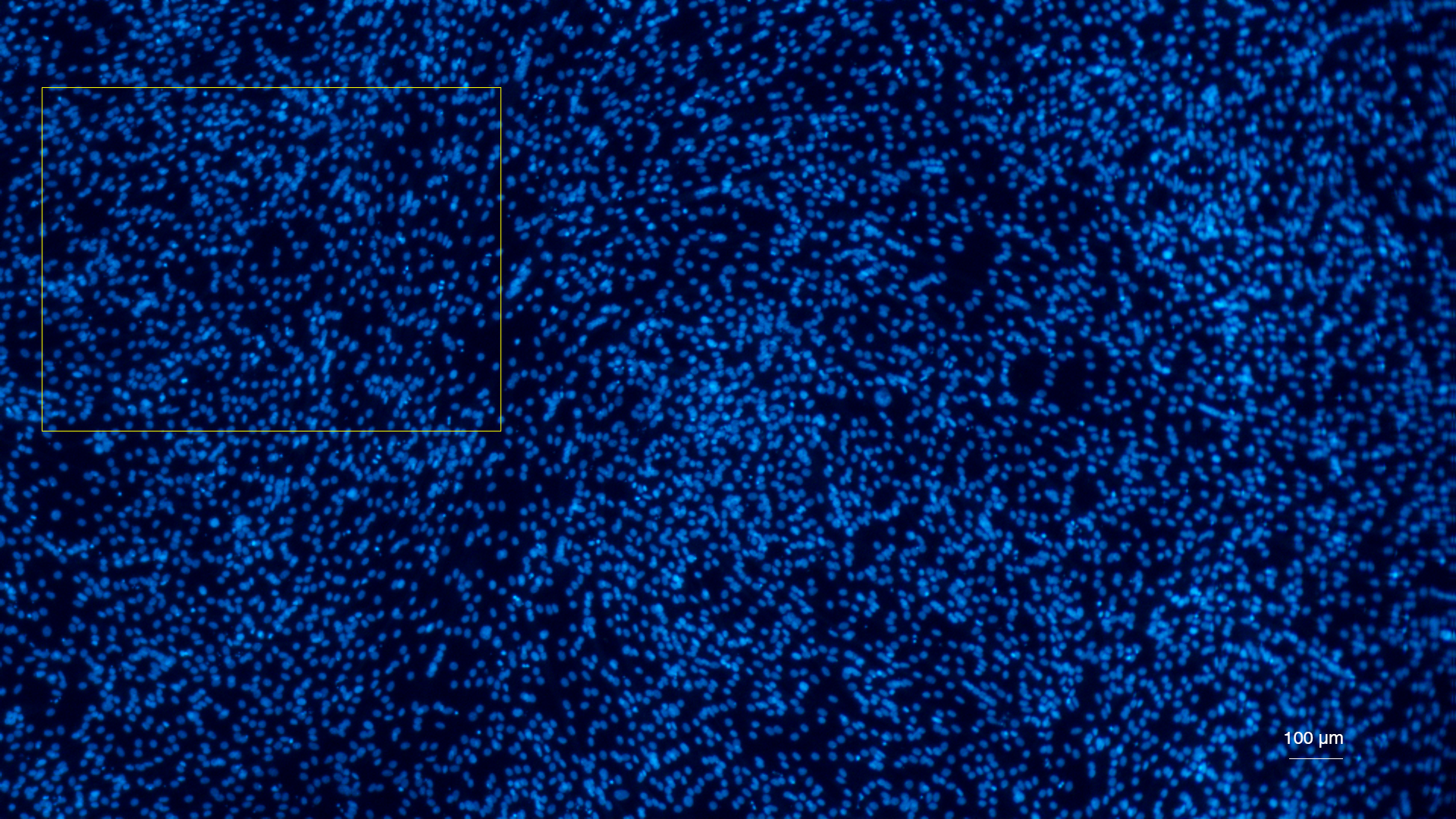

Supplement: Supplementary file 7 — Source data Fig. 2 [file 44318_2025_663_MOESM7_ESM.zip › EMBOJ-2025-121889_SourceDataForFigure2/2J/Il6st-pASO/DAPI.tif]

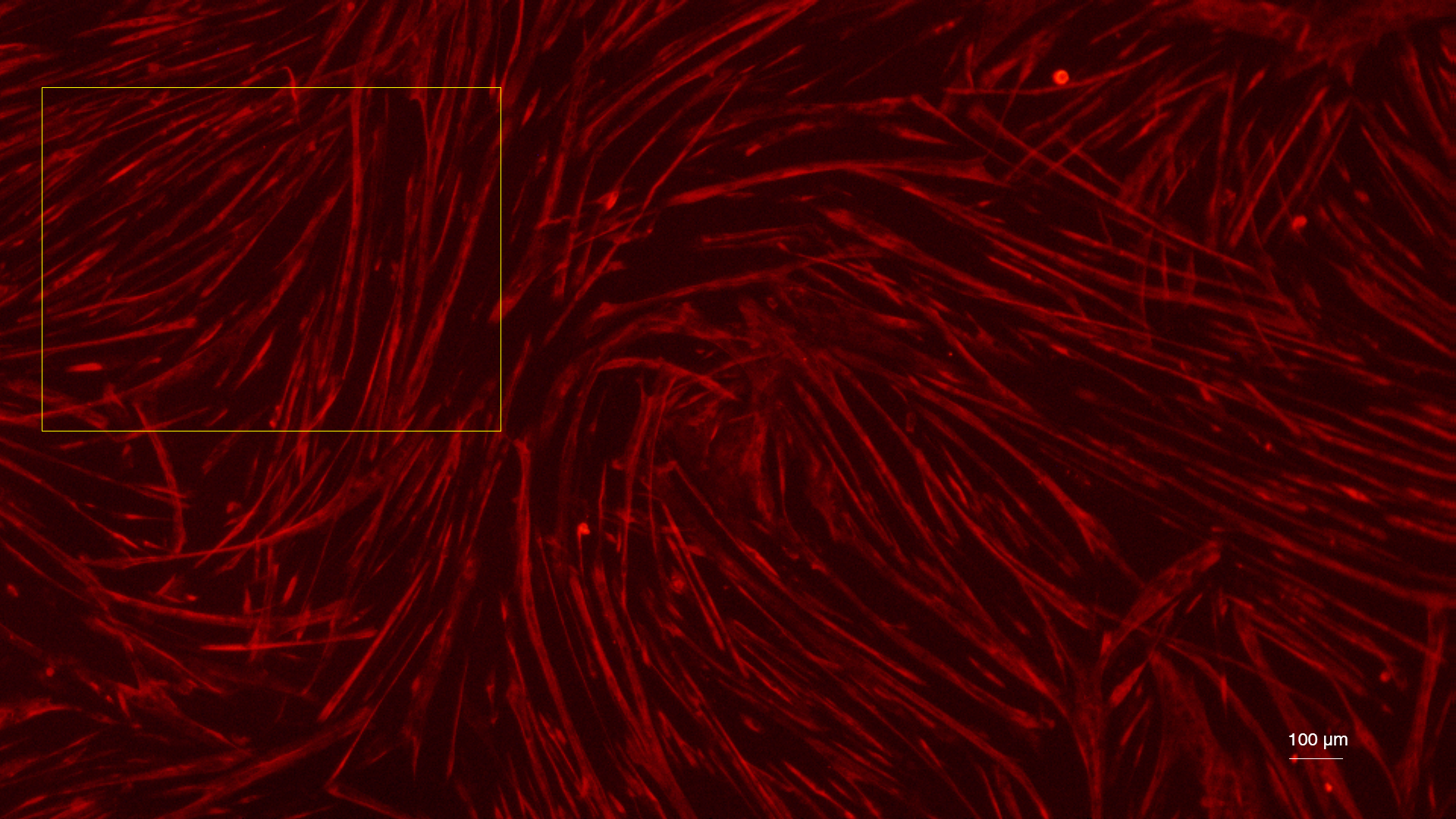

Supplement: Supplementary file 7 — Source data Fig. 2 [file 44318_2025_663_MOESM7_ESM.zip › EMBOJ-2025-121889_SourceDataForFigure2/2J/Il6st-pASO/MyHC.tif]

Source data for **Fig 3F**

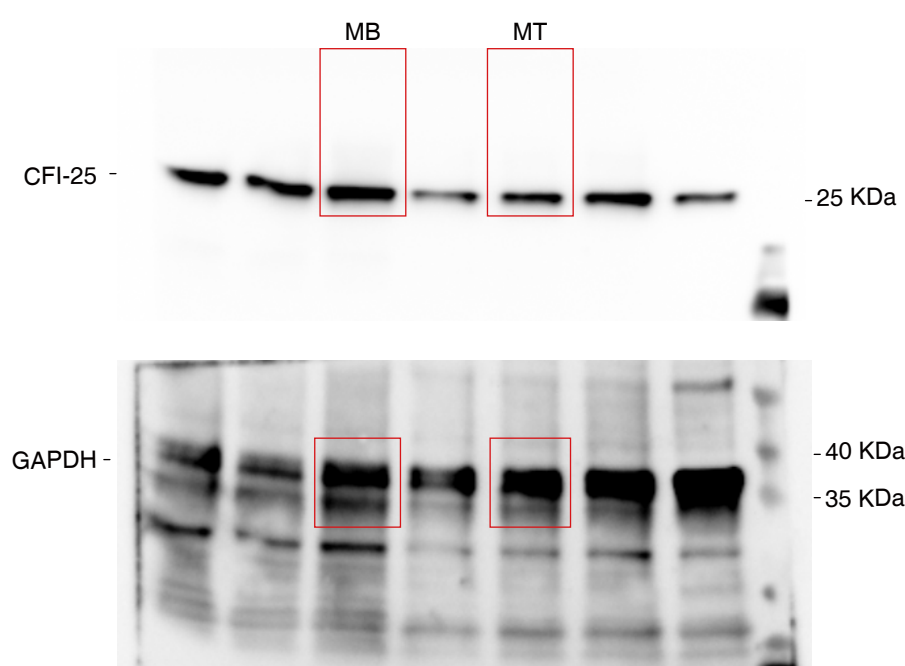

Supplement: Supplementary file 8 — Source data Fig. 3 [file 44318_2025_663_MOESM8_ESM.zip › EMBOJ-2025-121889_SourceDataForFigure3/3F/Western blot for 3F-CFI-25.pdf]

Source data for **Fig 3F**

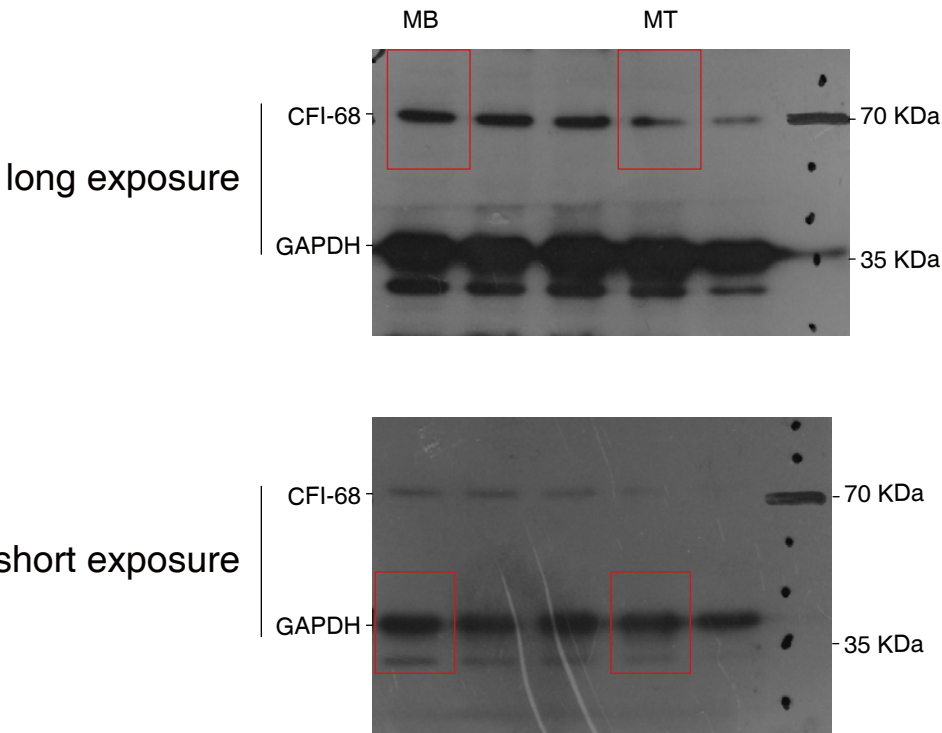

Supplement: Supplementary file 8 — Source data Fig. 3 [file 44318_2025_663_MOESM8_ESM.zip › EMBOJ-2025-121889_SourceDataForFigure3/3F/Western blot for 3F-CFI-68.pdf]

Source data for **Fig 5A**

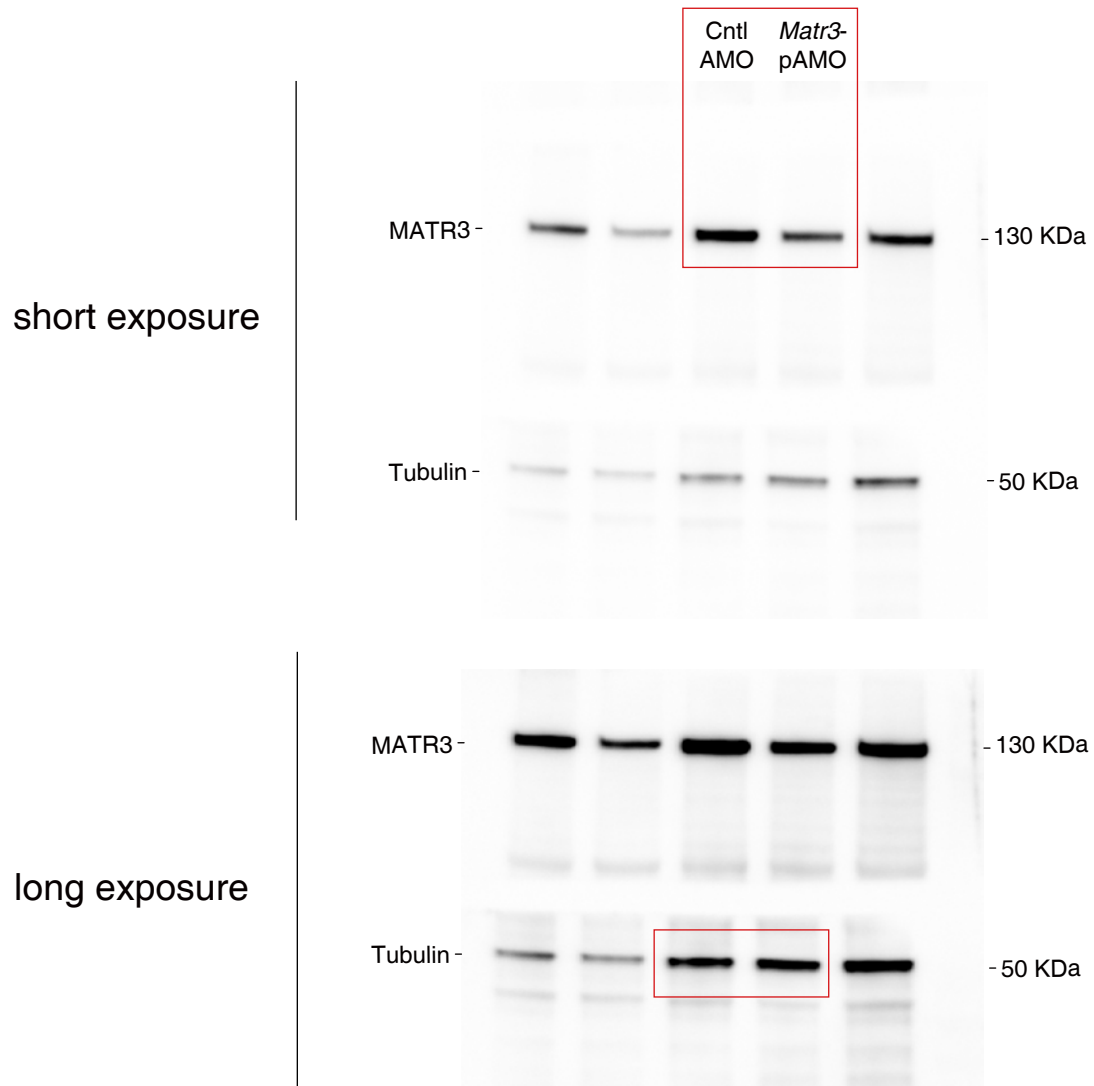

Supplement: Supplementary file 9 — Source data Fig. 5 [file 44318_2025_663_MOESM9_ESM.zip › EMBOJ-2025-121889_SourceDataForFigure5/5A/Western blot for 5A.pdf]

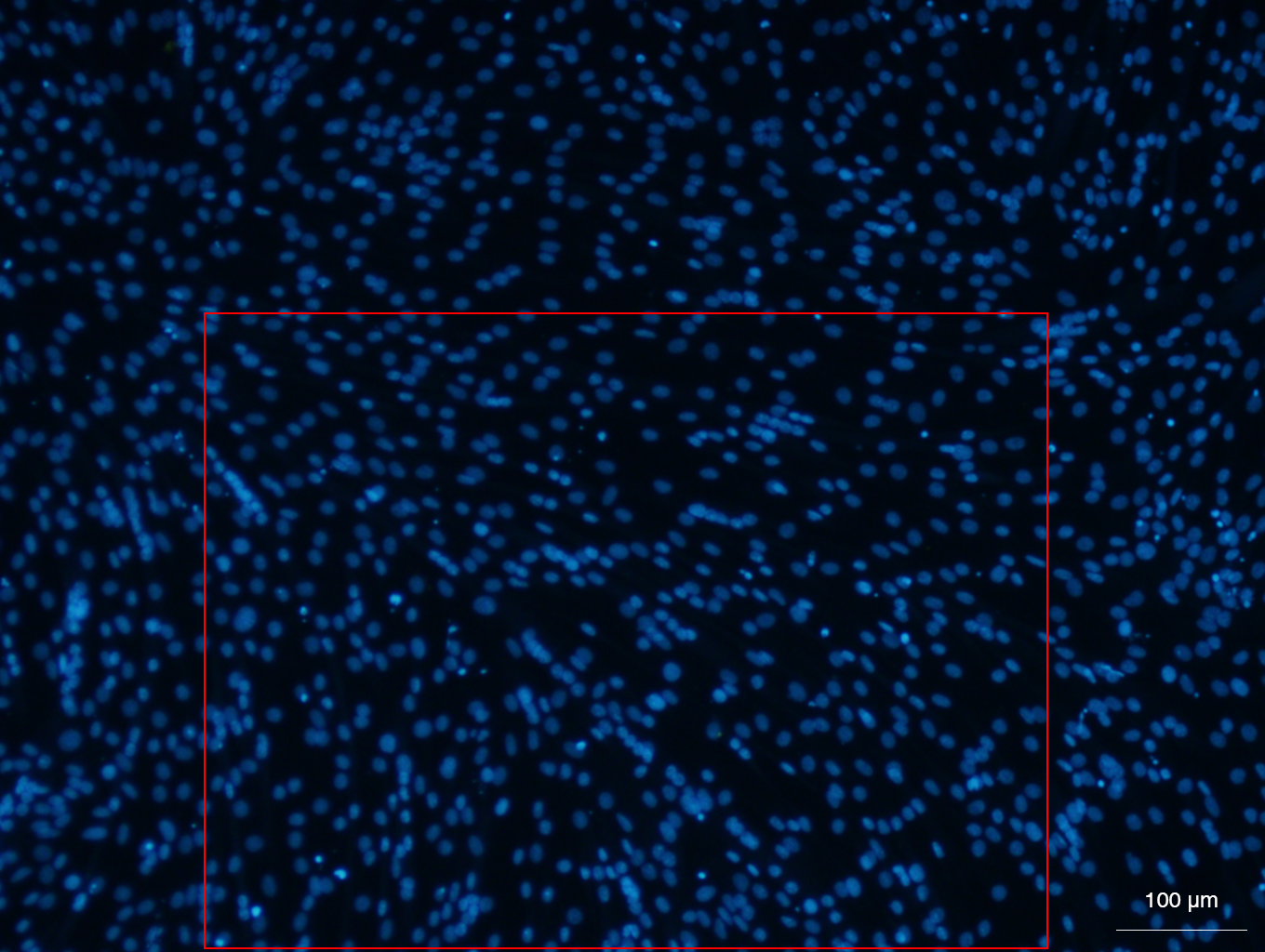

Supplement: Supplementary file 9 — Source data Fig. 5 [file 44318_2025_663_MOESM9_ESM.zip › EMBOJ-2025-121889_SourceDataForFigure5/5B/Cntl KD/DAPI.tif]

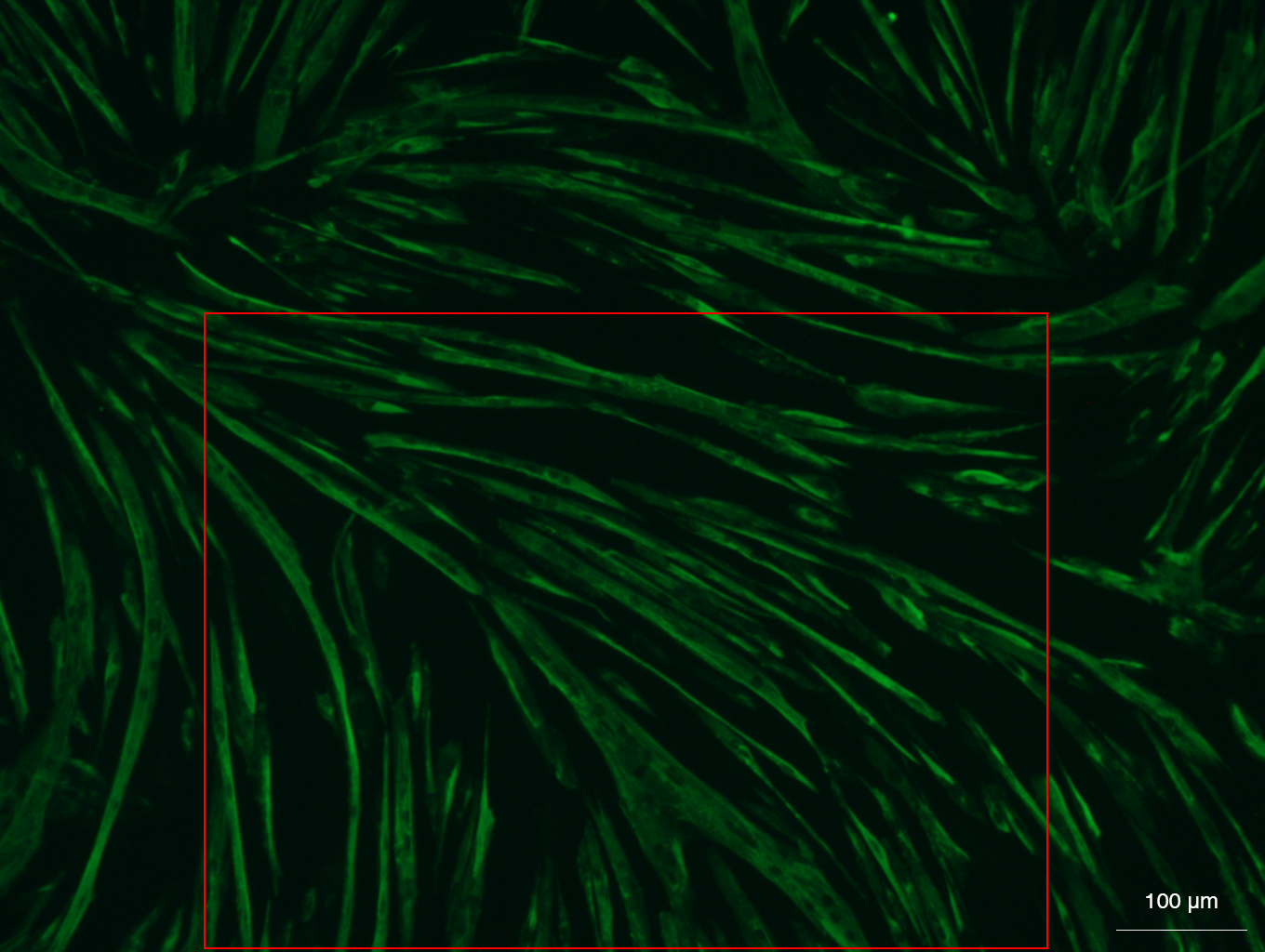

Supplement: Supplementary file 9 — Source data Fig. 5 [file 44318_2025_663_MOESM9_ESM.zip › EMBOJ-2025-121889_SourceDataForFigure5/5B/Cntl KD/MyHC.tif]

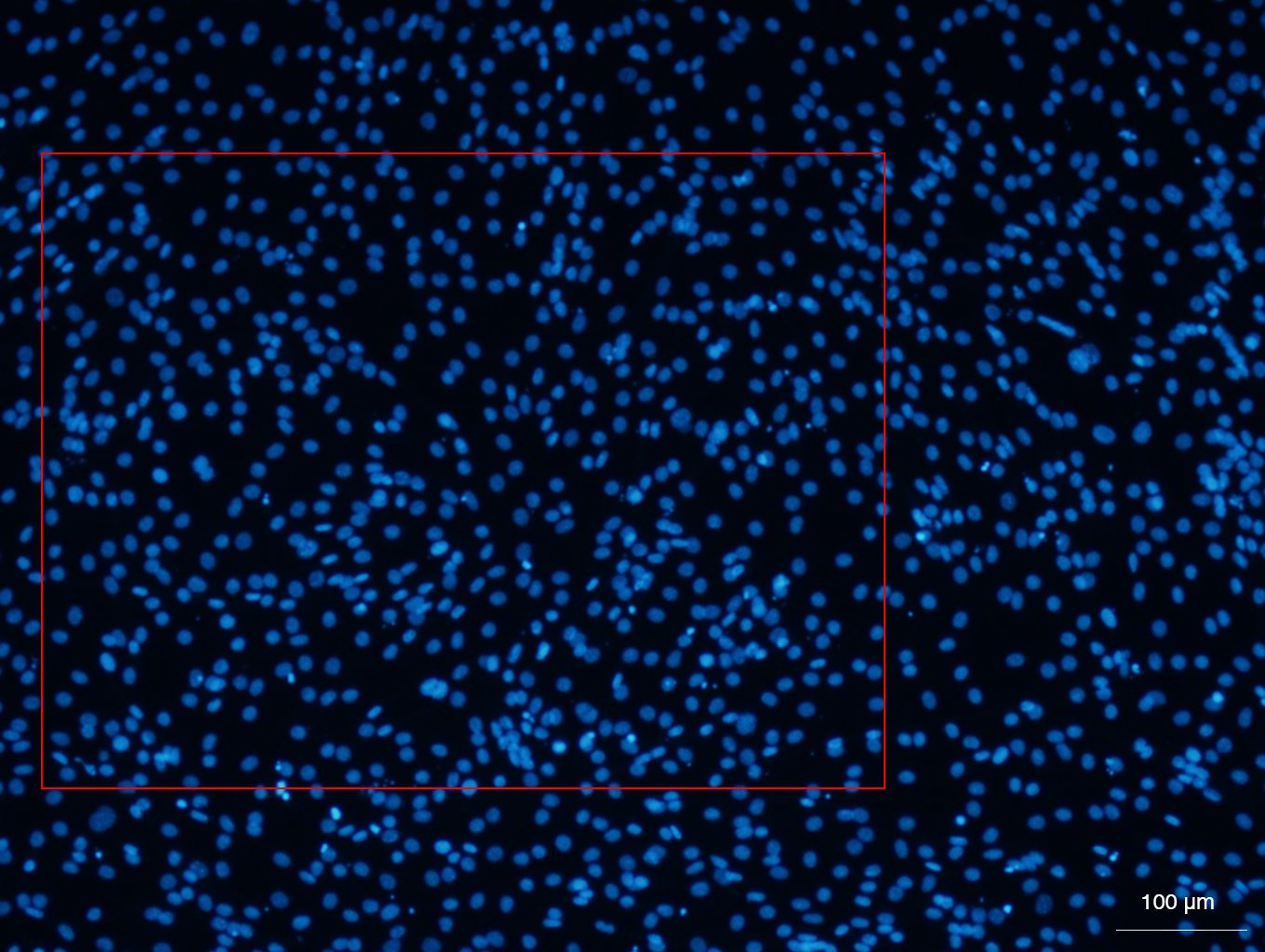

Supplement: Supplementary file 9 — Source data Fig. 5 [file 44318_2025_663_MOESM9_ESM.zip › EMBOJ-2025-121889_SourceDataForFigure5/5B/Matr3 KD #1/DAPI.tif]

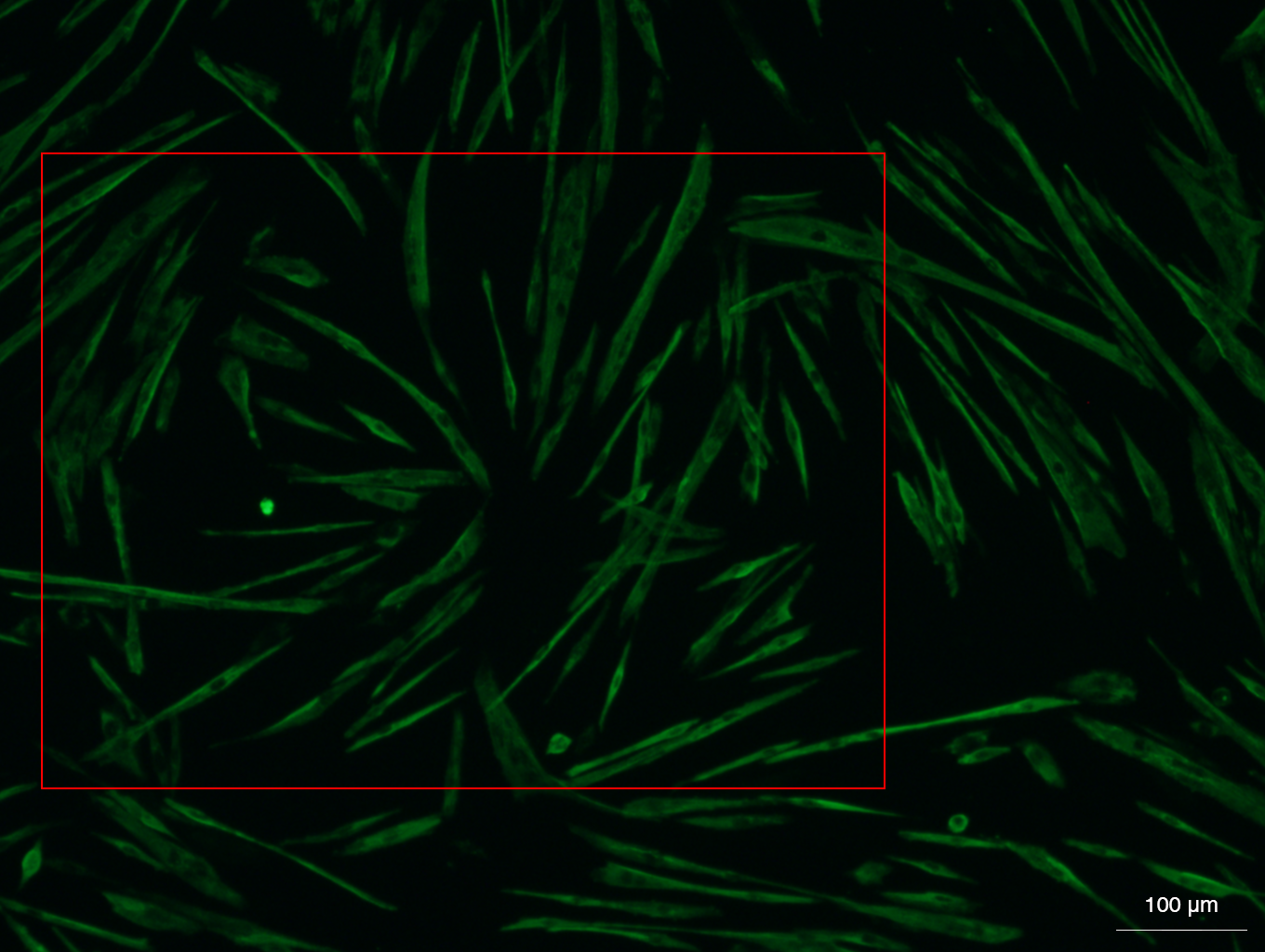

Supplement: Supplementary file 9 — Source data Fig. 5 [file 44318_2025_663_MOESM9_ESM.zip › EMBOJ-2025-121889_SourceDataForFigure5/5B/Matr3 KD #1/MyHC.tif]

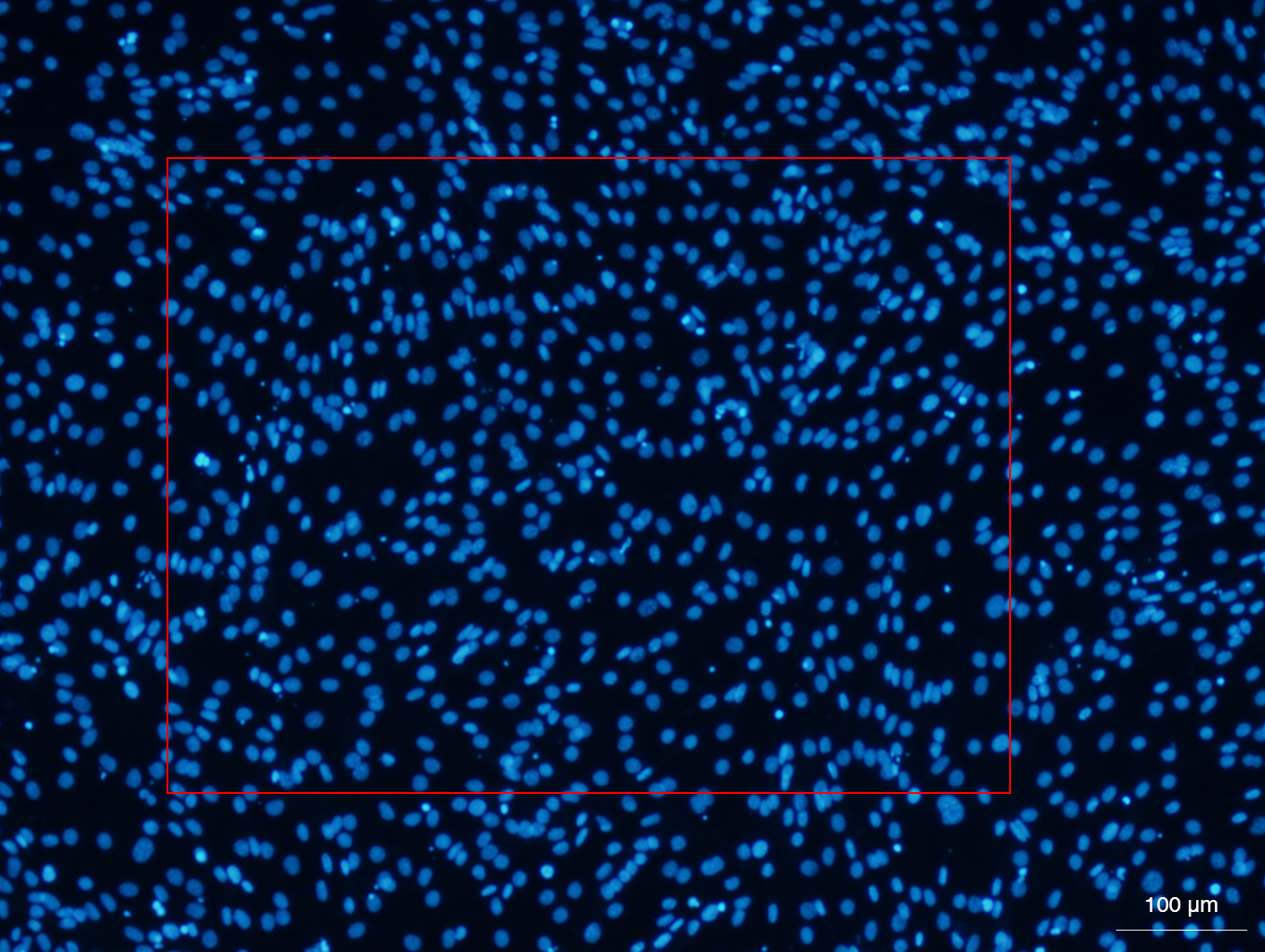

Supplement: Supplementary file 9 — Source data Fig. 5 [file 44318_2025_663_MOESM9_ESM.zip › EMBOJ-2025-121889_SourceDataForFigure5/5B/Matr3 KD #2/DAPI.tif]

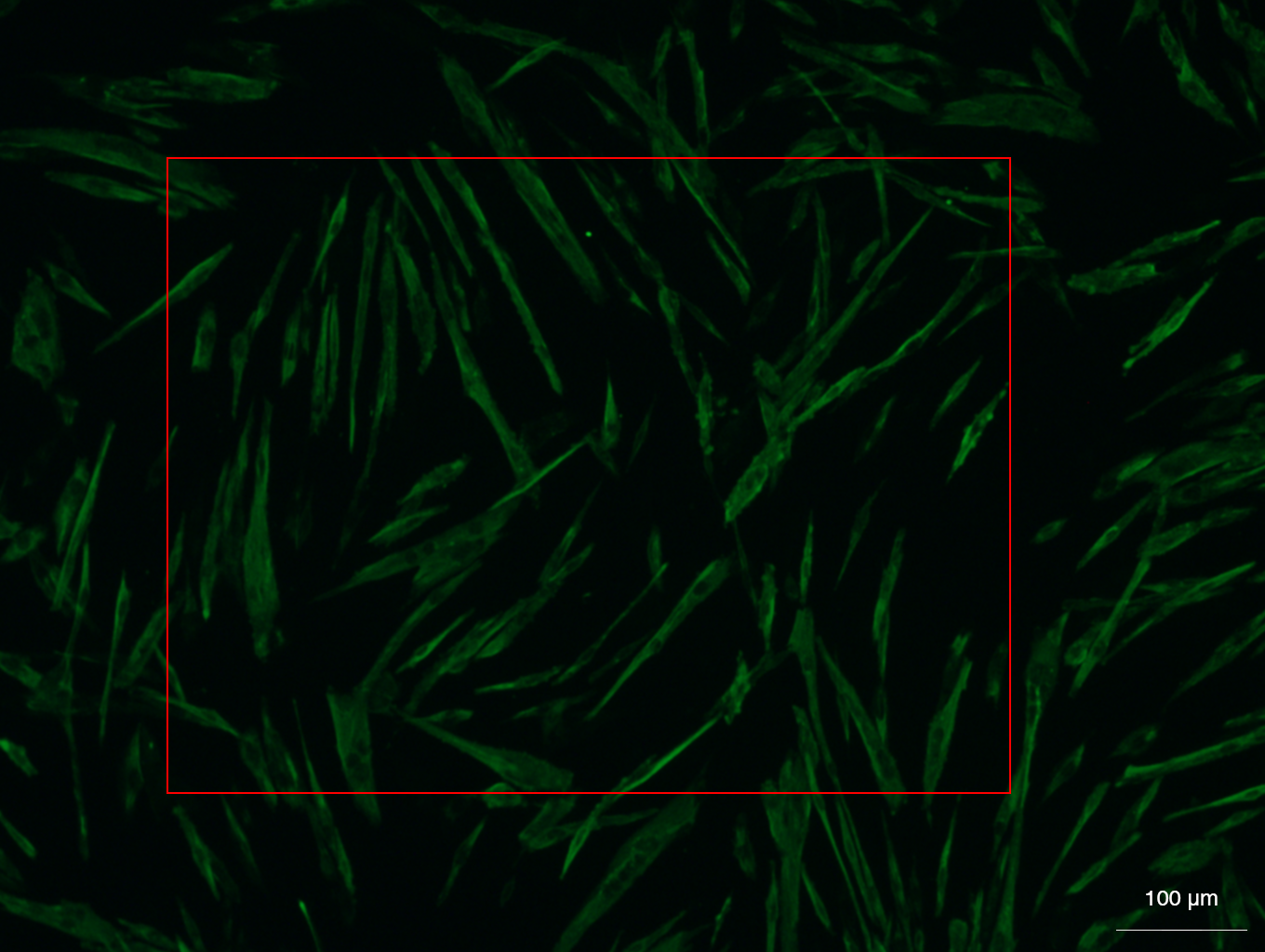

Supplement: Supplementary file 9 — Source data Fig. 5 [file 44318_2025_663_MOESM9_ESM.zip › EMBOJ-2025-121889_SourceDataForFigure5/5B/Matr3 KD #2/MyHC.tif]

Source data for **Fig 5H**

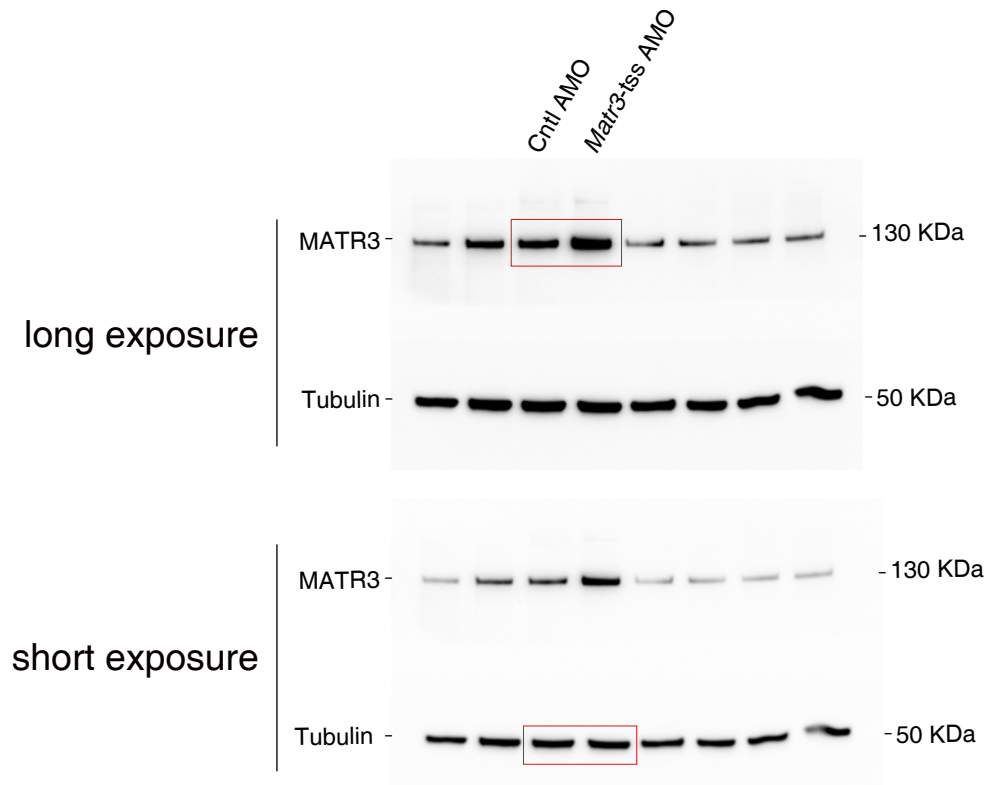

Supplement: Supplementary file 9 — Source data Fig. 5 [file 44318_2025_663_MOESM9_ESM.zip › EMBOJ-2025-121889_SourceDataForFigure5/5H/Western blot for 5H.pdf]

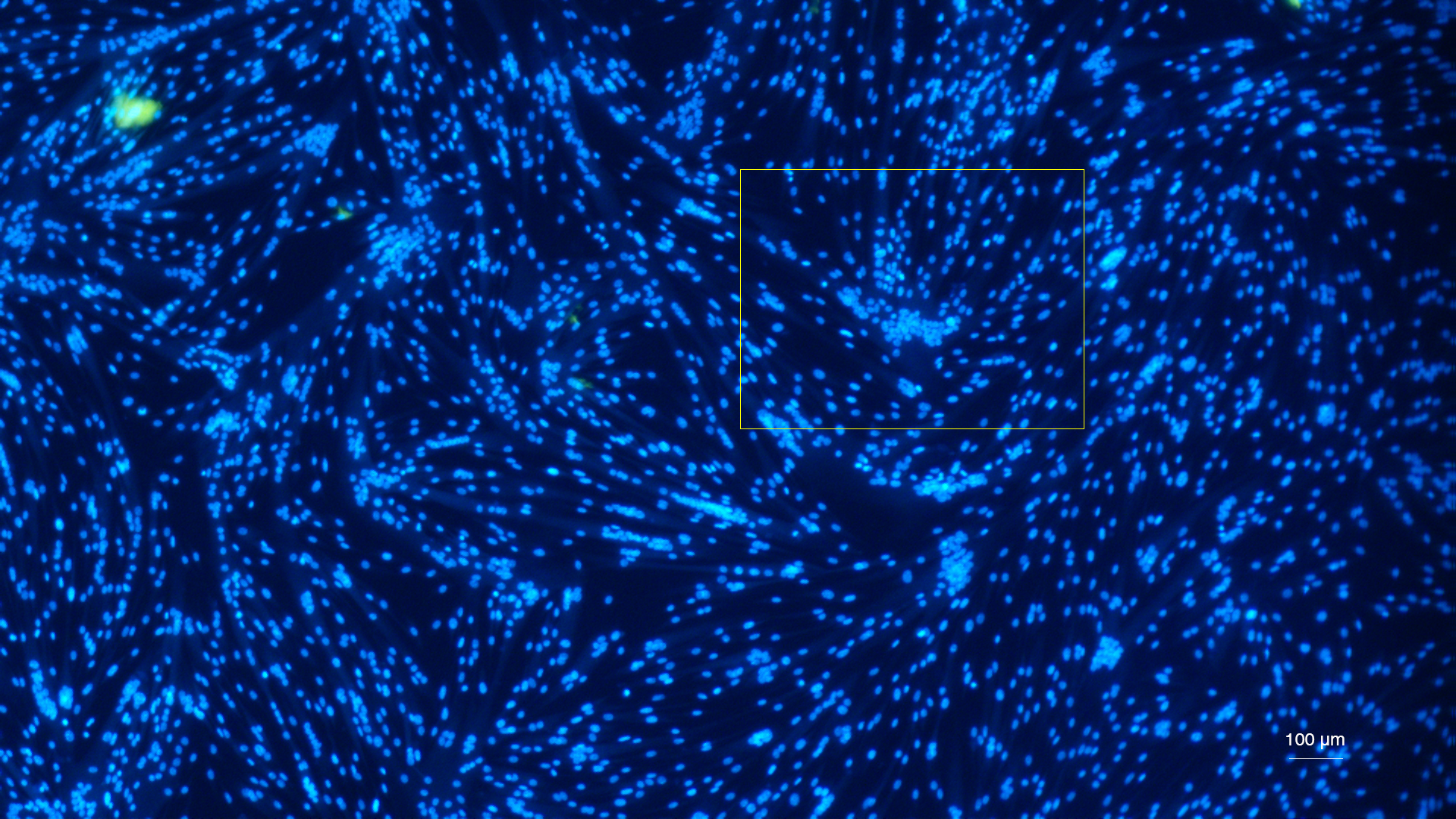

Supplement: Supplementary file 9 — Source data Fig. 5 [file 44318_2025_663_MOESM9_ESM.zip › EMBOJ-2025-121889_SourceDataForFigure5/5I/Cntl AMO/DAPI.tif]

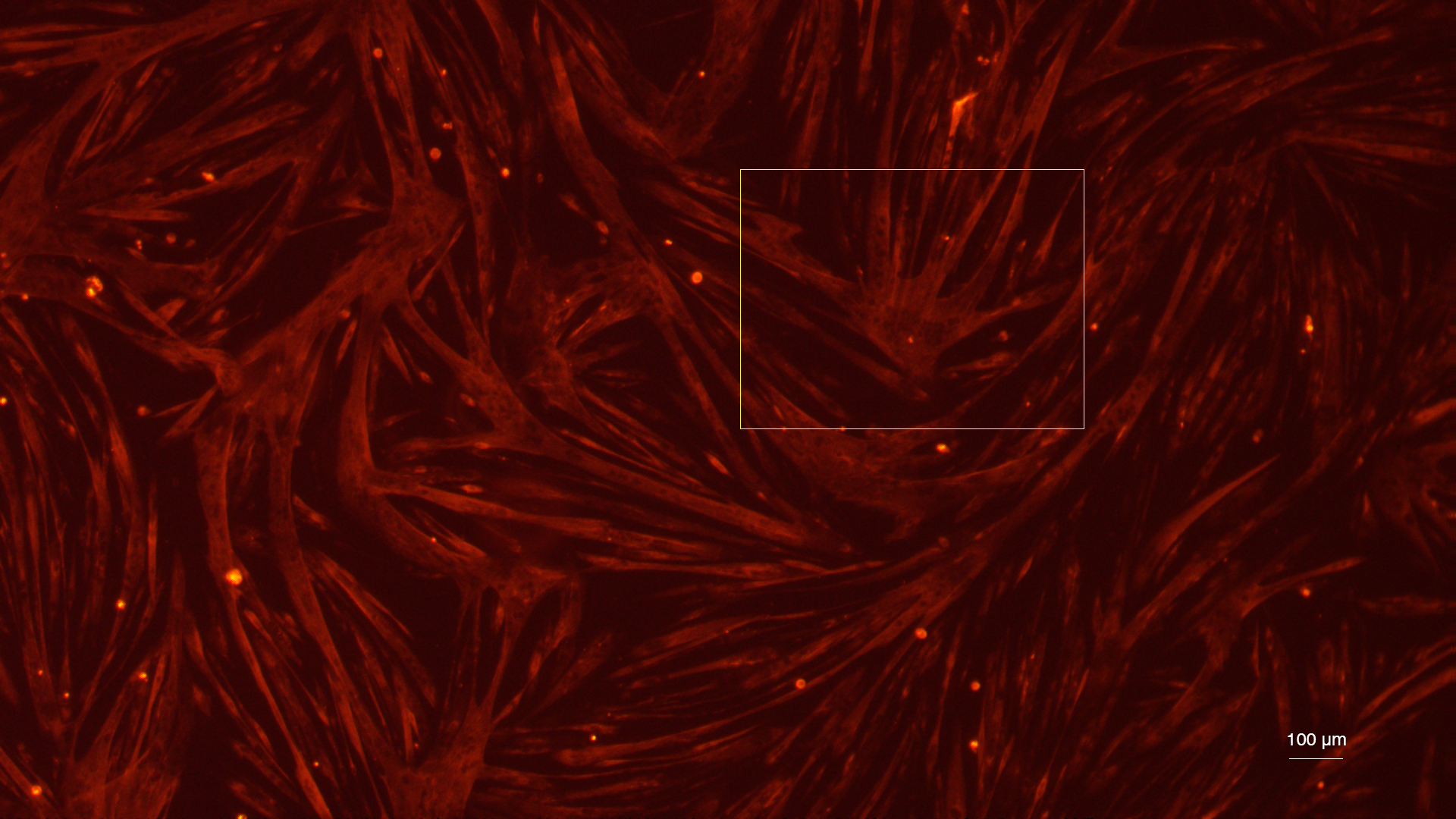

Supplement: Supplementary file 9 — Source data Fig. 5 [file 44318_2025_663_MOESM9_ESM.zip › EMBOJ-2025-121889_SourceDataForFigure5/5I/Cntl AMO/MyHC.tif]

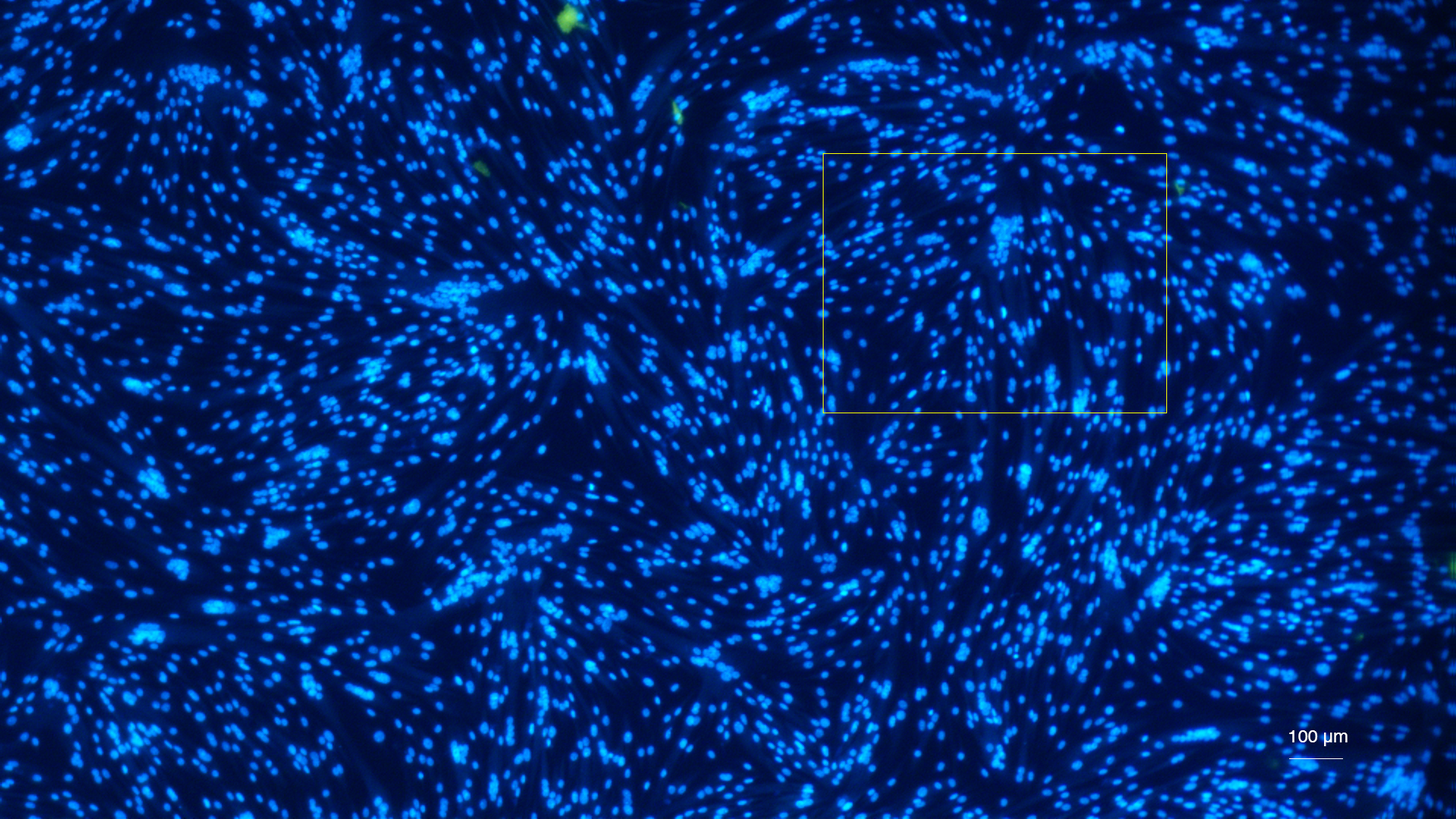

Supplement: Supplementary file 9 — Source data Fig. 5 [file 44318_2025_663_MOESM9_ESM.zip › EMBOJ-2025-121889_SourceDataForFigure5/5I/Matr3-tss AMO/DAPI.tif]

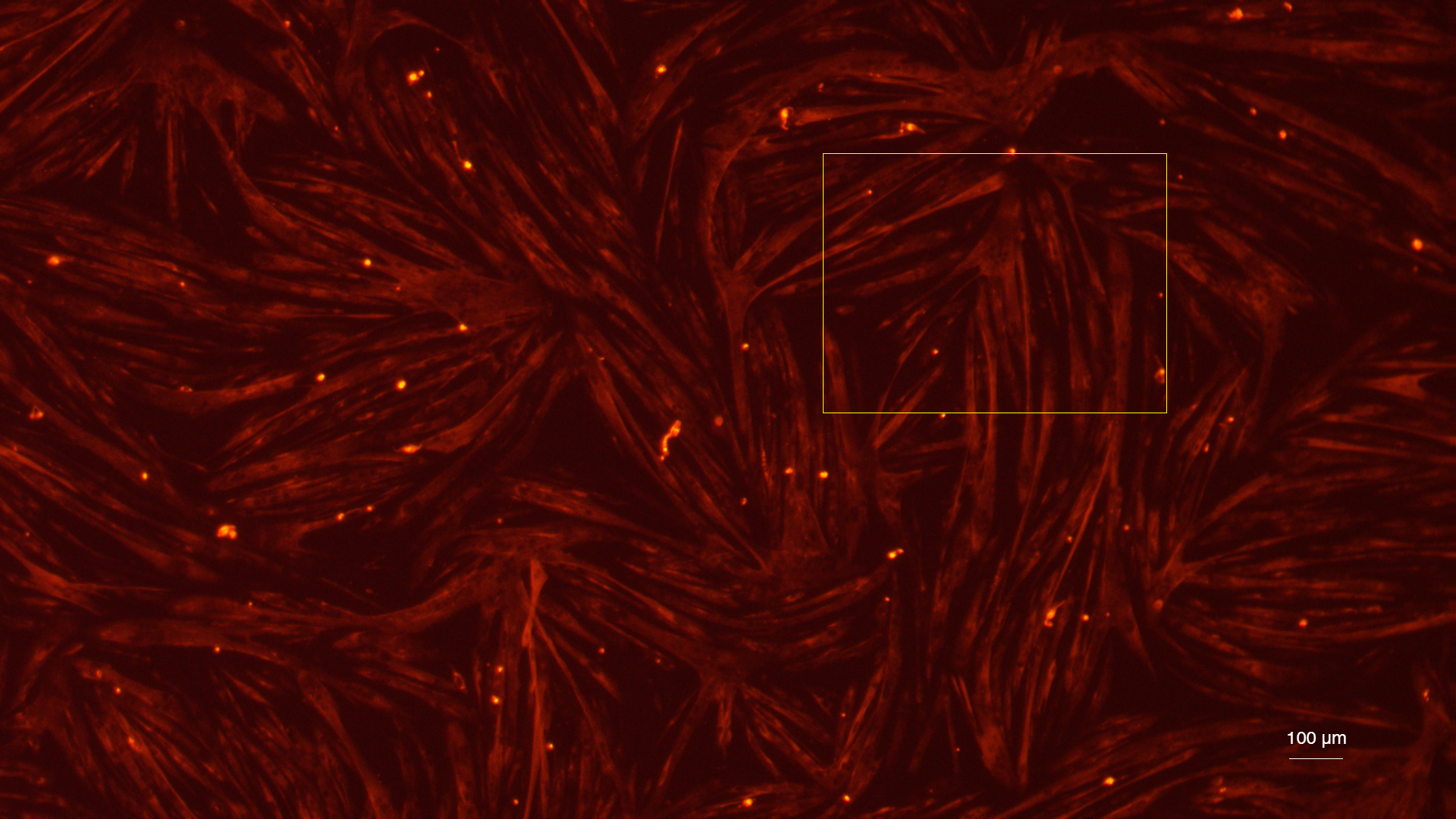

Supplement: Supplementary file 9 — Source data Fig. 5 [file 44318_2025_663_MOESM9_ESM.zip › EMBOJ-2025-121889_SourceDataForFigure5/5I/Matr3-tss AMO/MyHC.tif]

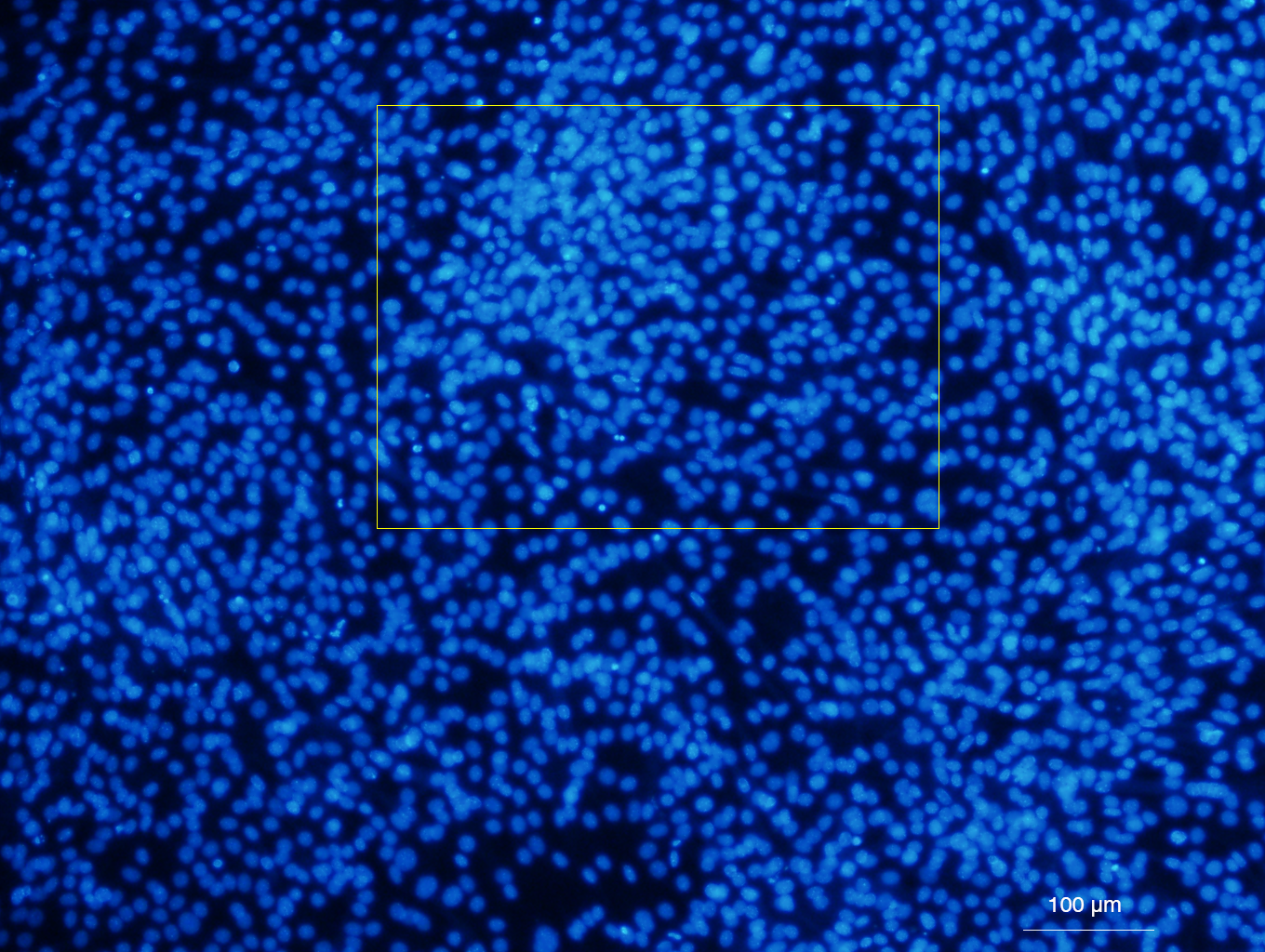

Supplement: Supplementary file 9 — Source data Fig. 5 [file 44318_2025_663_MOESM9_ESM.zip › EMBOJ-2025-121889_SourceDataForFigure5/5L/Cntl OE/DAPI.tif]

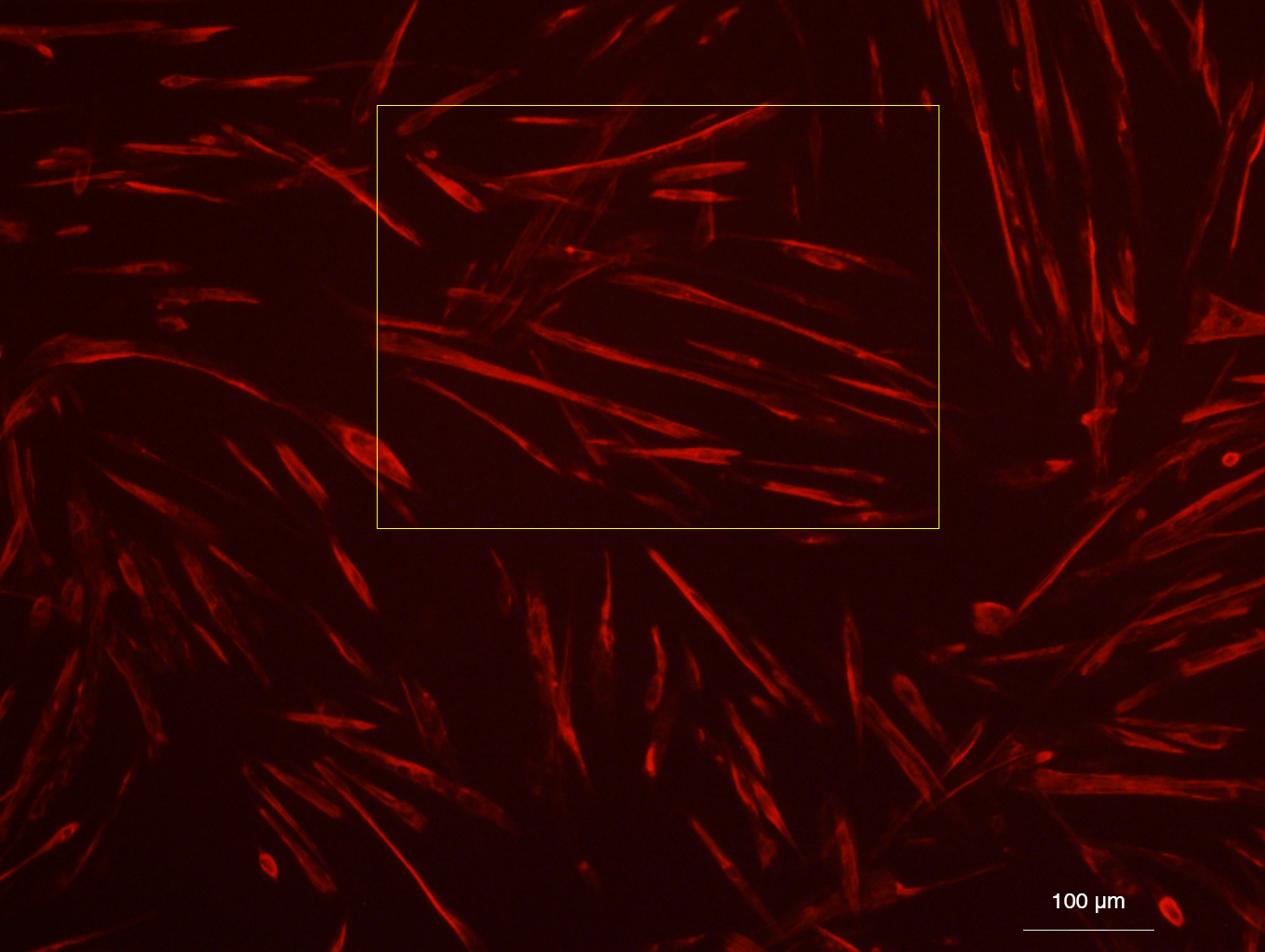

Supplement: Supplementary file 9 — Source data Fig. 5 [file 44318_2025_663_MOESM9_ESM.zip › EMBOJ-2025-121889_SourceDataForFigure5/5L/Cntl OE/MyHC.tif]

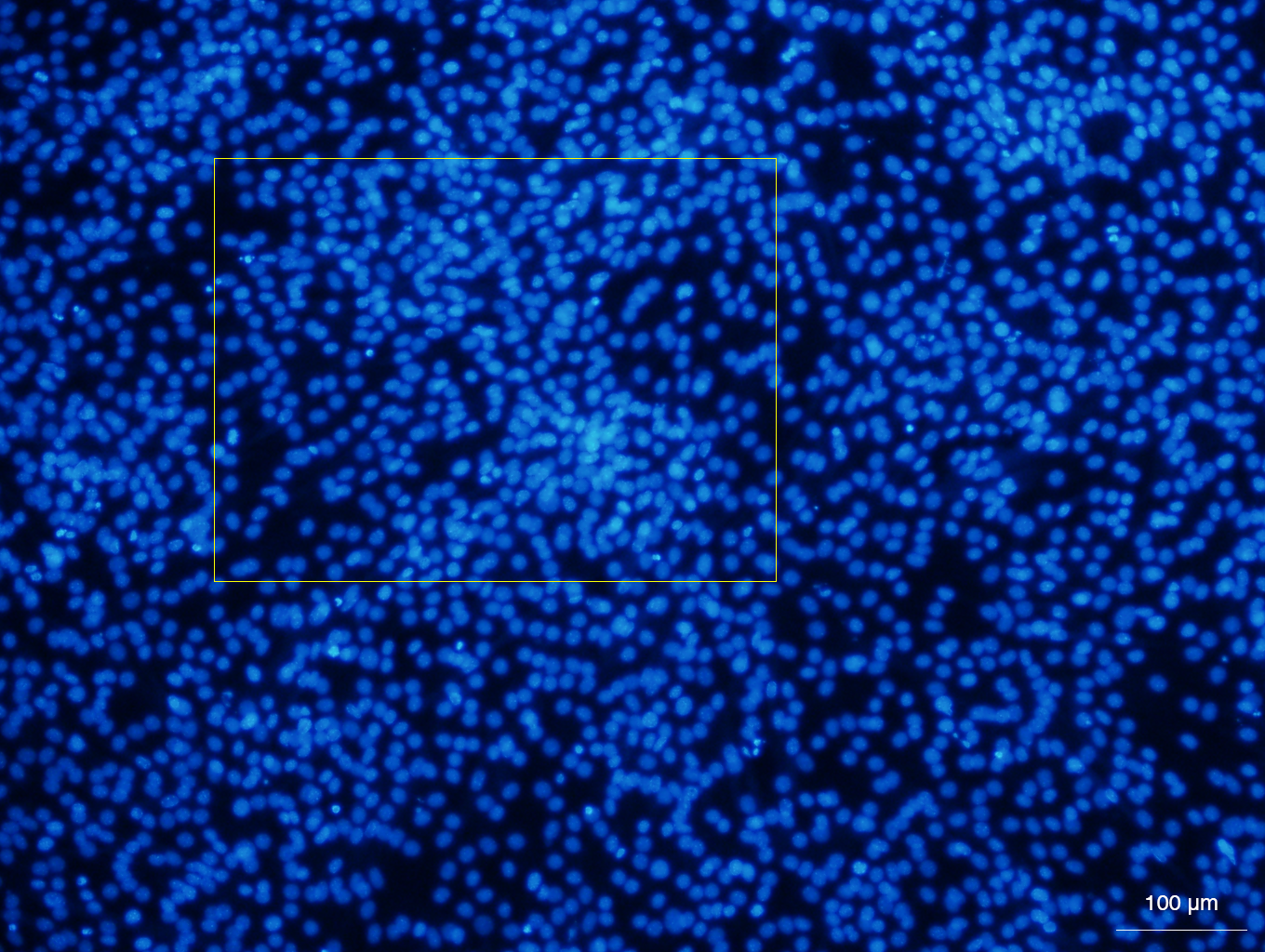

Supplement: Supplementary file 9 — Source data Fig. 5 [file 44318_2025_663_MOESM9_ESM.zip › EMBOJ-2025-121889_SourceDataForFigure5/5L/Matr3 OE/DAPI.tif]

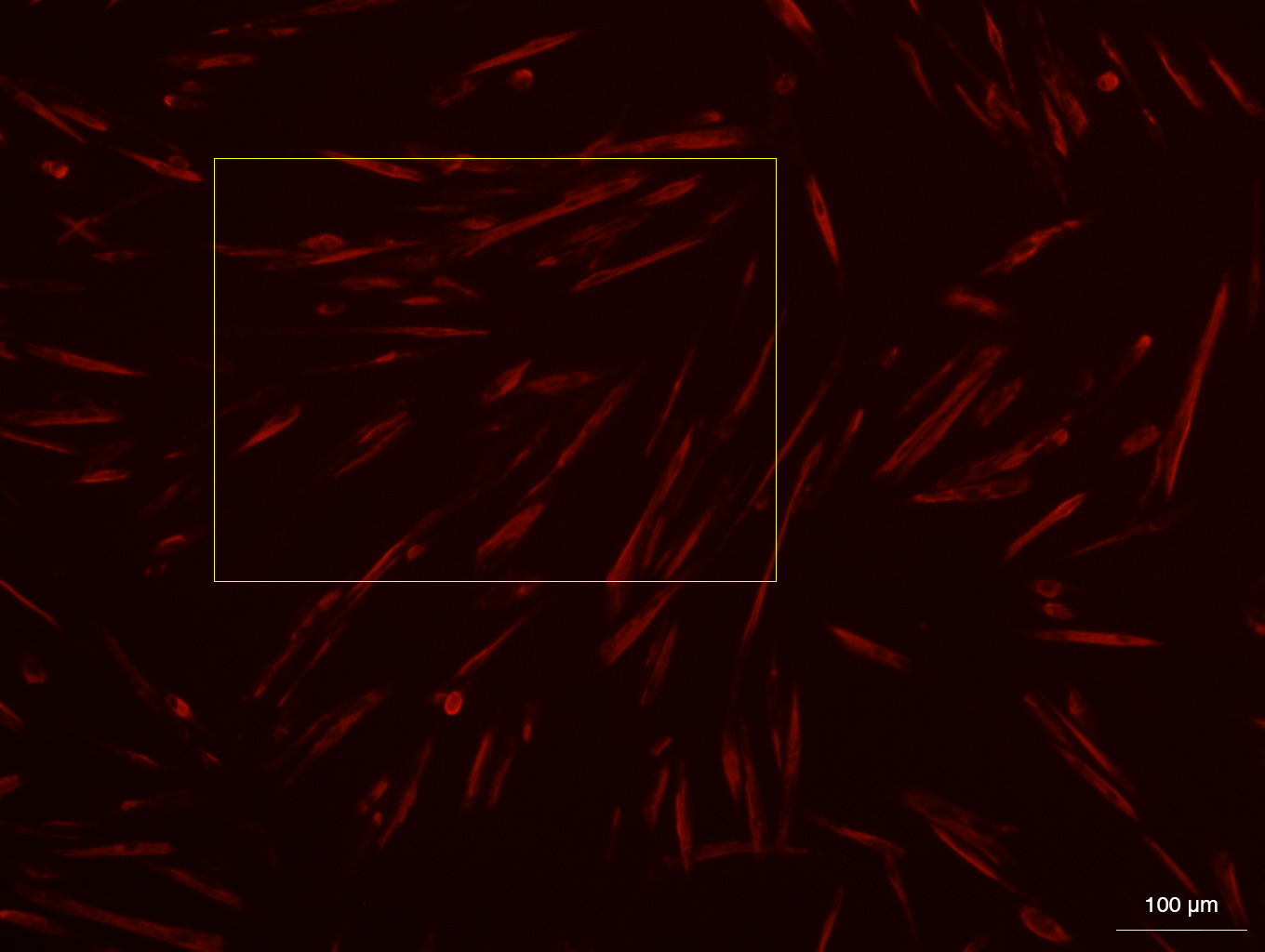

Supplement: Supplementary file 9 — Source data Fig. 5 [file 44318_2025_663_MOESM9_ESM.zip › EMBOJ-2025-121889_SourceDataForFigure5/5L/Matr3 OE/MyHC.tif]

Source data for **Fig 6B**

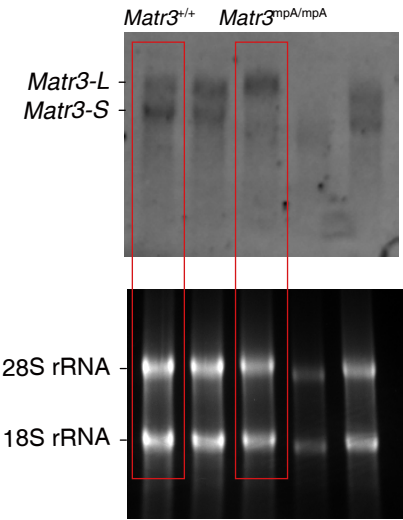

Supplement: Supplementary file 10 — Source data Fig. 6 [file 44318_2025_663_MOESM10_ESM.zip › EMBOJ-2025-121889_SourceDataForFigure6/6B/Northern blot for 6B.pdf]

Source data for **Fig 6C**

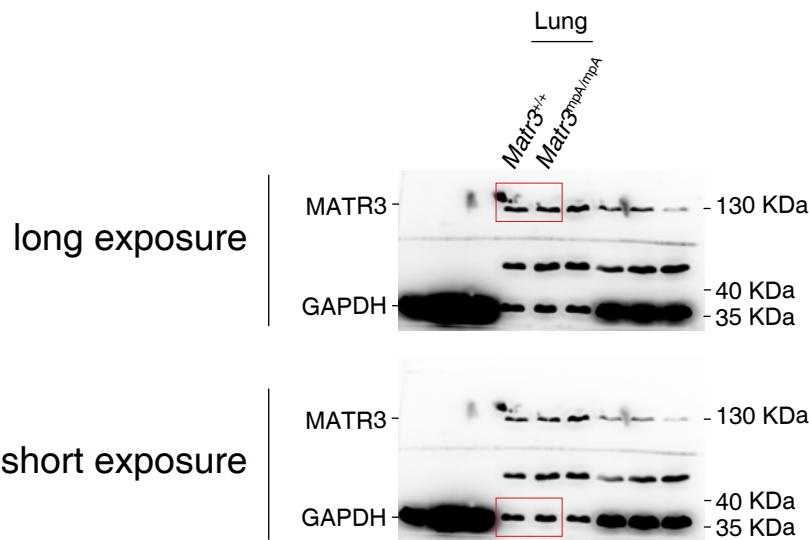

Supplement: Supplementary file 10 — Source data Fig. 6 [file 44318_2025_663_MOESM10_ESM.zip › EMBOJ-2025-121889_SourceDataForFigure6/6C/Western blot for 6C-Lung.pdf]

Source data for **Fig 6C**

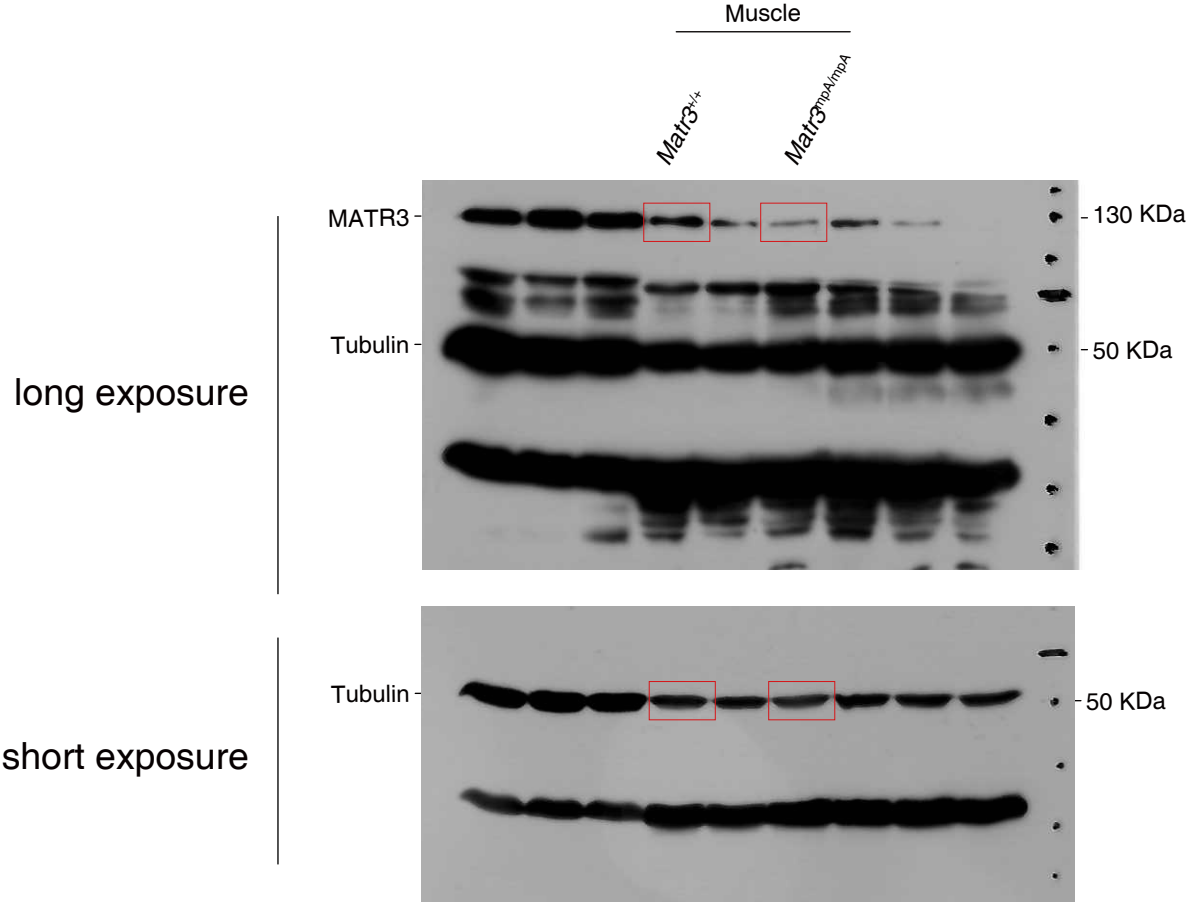

Supplement: Supplementary file 10 — Source data Fig. 6 [file 44318_2025_663_MOESM10_ESM.zip › EMBOJ-2025-121889_SourceDataForFigure6/6C/Western blot for 6C-Muscle.pdf]

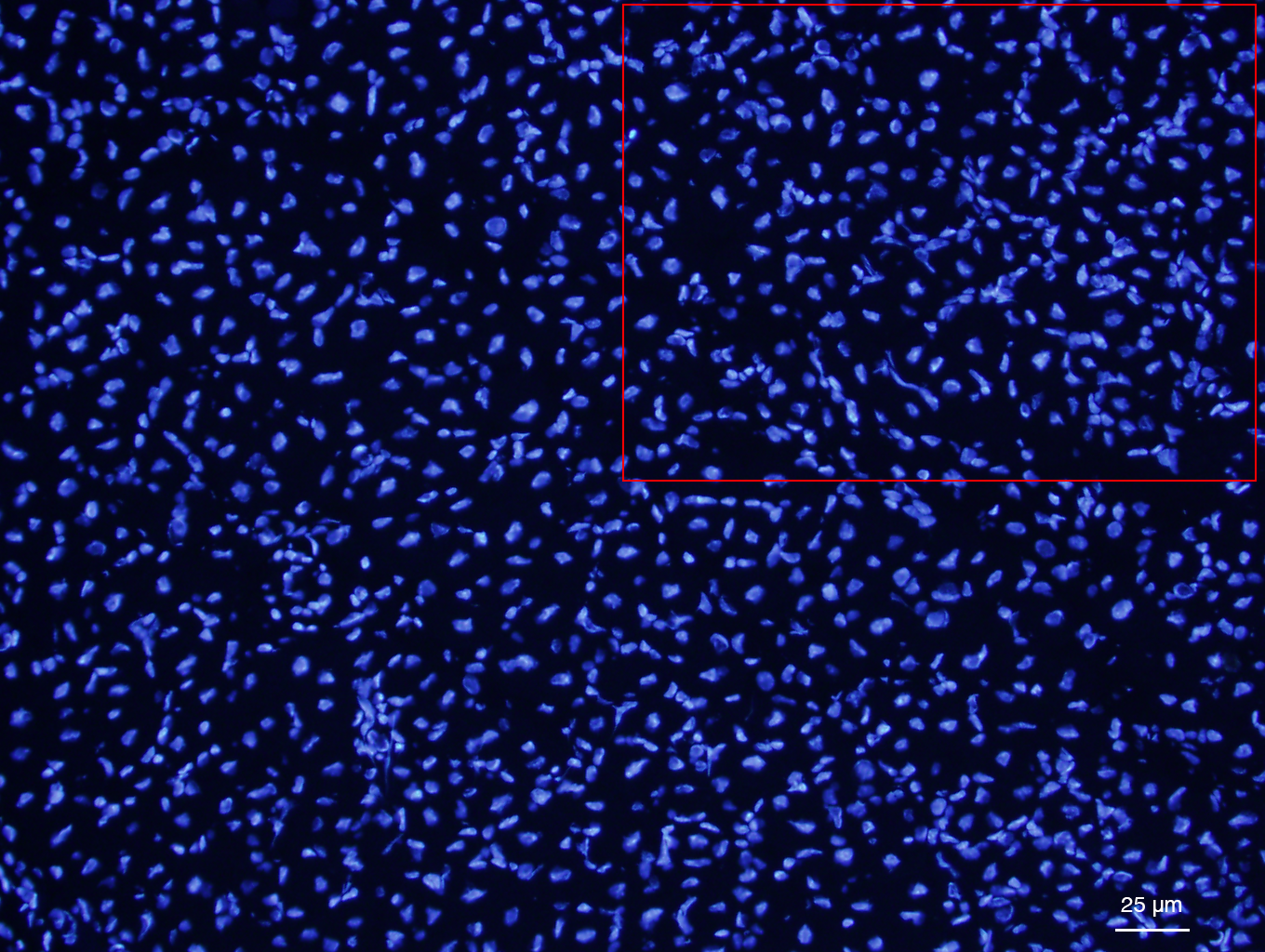

Supplement: Supplementary file 10 — Source data Fig. 6 [file 44318_2025_663_MOESM10_ESM.zip › EMBOJ-2025-121889_SourceDataForFigure6/6D/Matr3 mutant mice (mpA)/DAPI.tif]

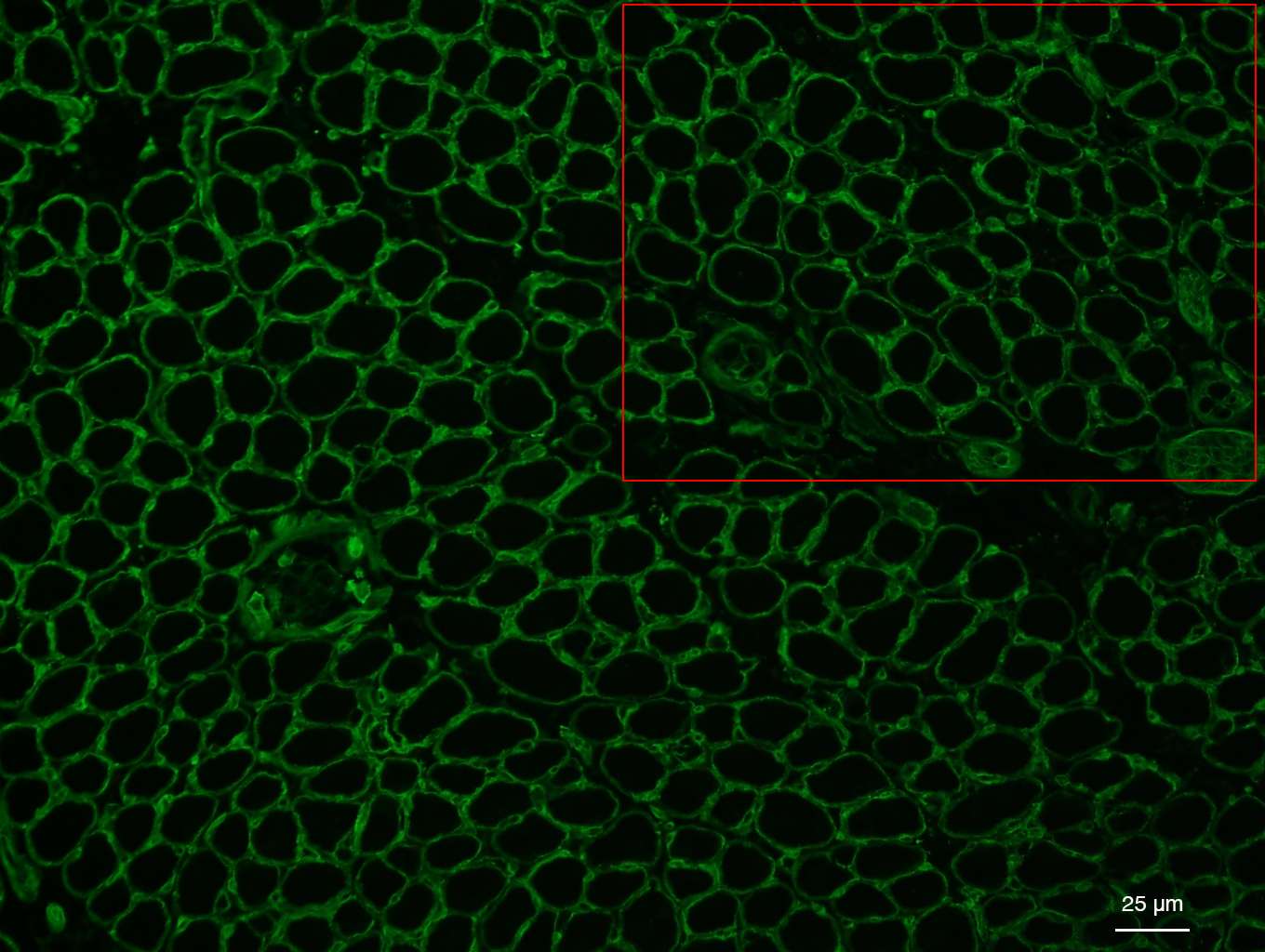

Supplement: Supplementary file 10 — Source data Fig. 6 [file 44318_2025_663_MOESM10_ESM.zip › EMBOJ-2025-121889_SourceDataForFigure6/6D/Matr3 mutant mice (mpA)/Laminin.tif]

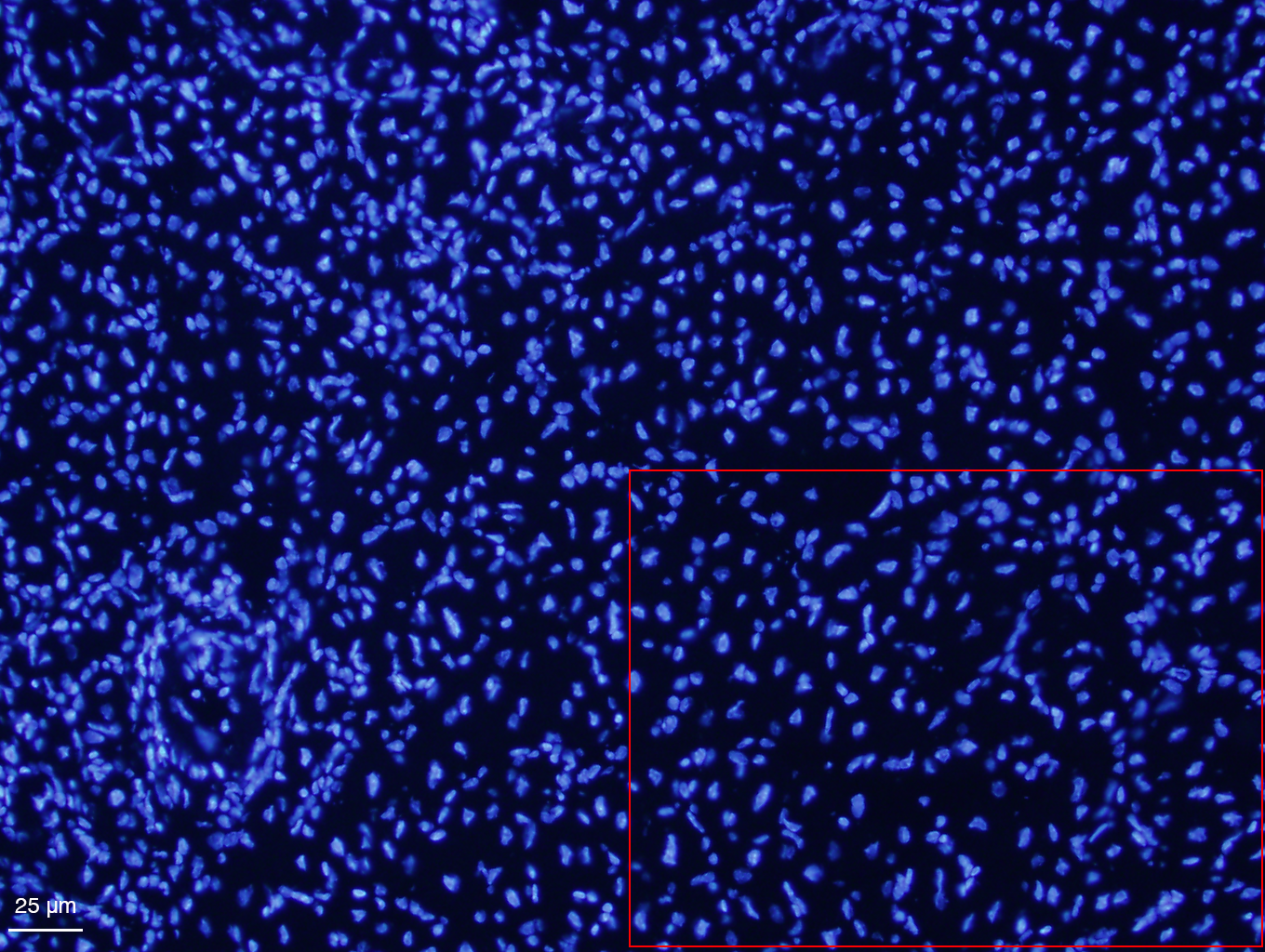

Supplement: Supplementary file 10 — Source data Fig. 6 [file 44318_2025_663_MOESM10_ESM.zip › EMBOJ-2025-121889_SourceDataForFigure6/6D/WT mice (+)/DAPI.tif]

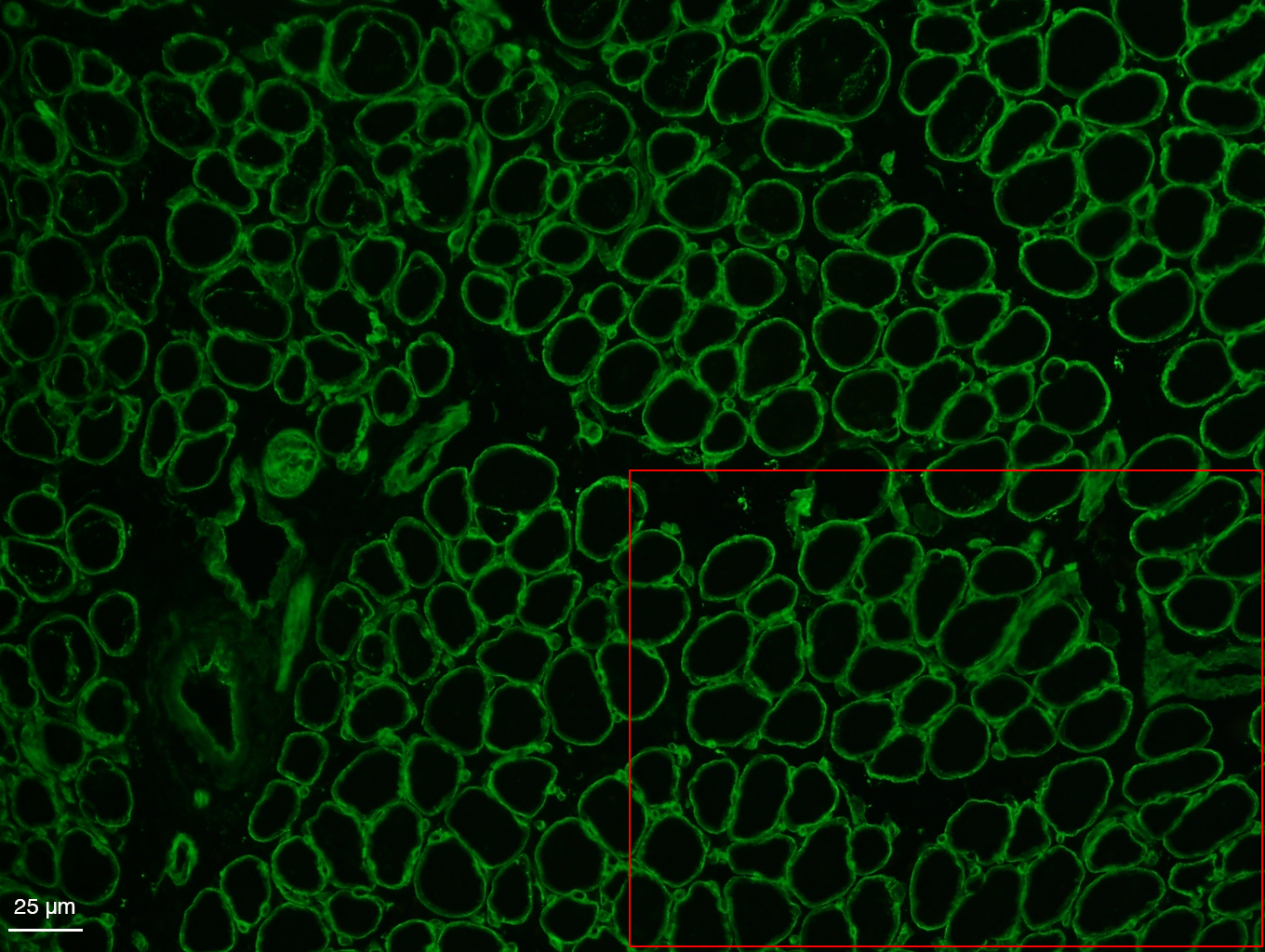

Supplement: Supplementary file 10 — Source data Fig. 6 [file 44318_2025_663_MOESM10_ESM.zip › EMBOJ-2025-121889_SourceDataForFigure6/6D/WT mice (+)/Laminin.tif]

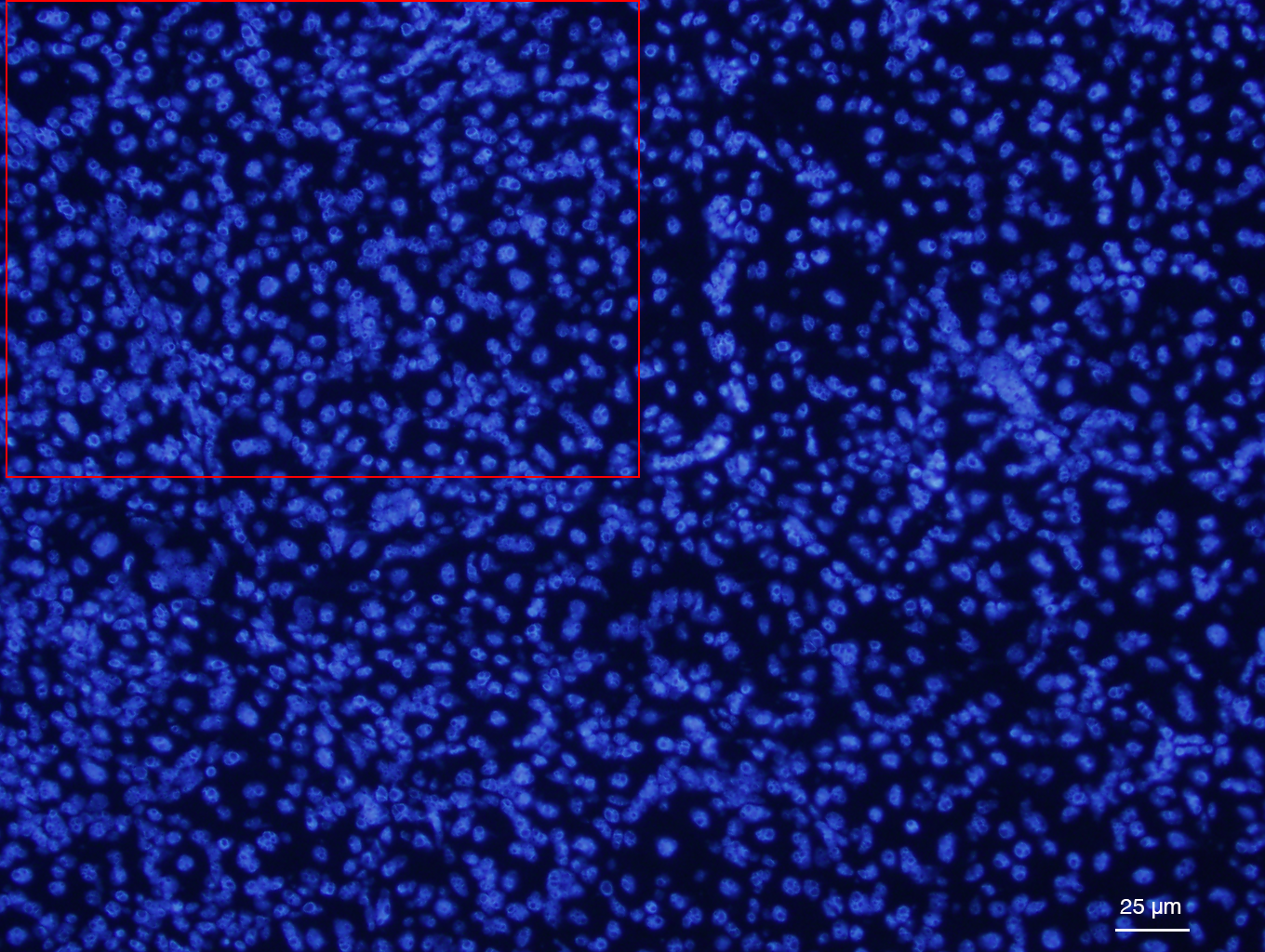

Supplement: Supplementary file 10 — Source data Fig. 6 [file 44318_2025_663_MOESM10_ESM.zip › EMBOJ-2025-121889_SourceDataForFigure6/6F/Matr3 mutant mice (mpA)/DAPI.tif]

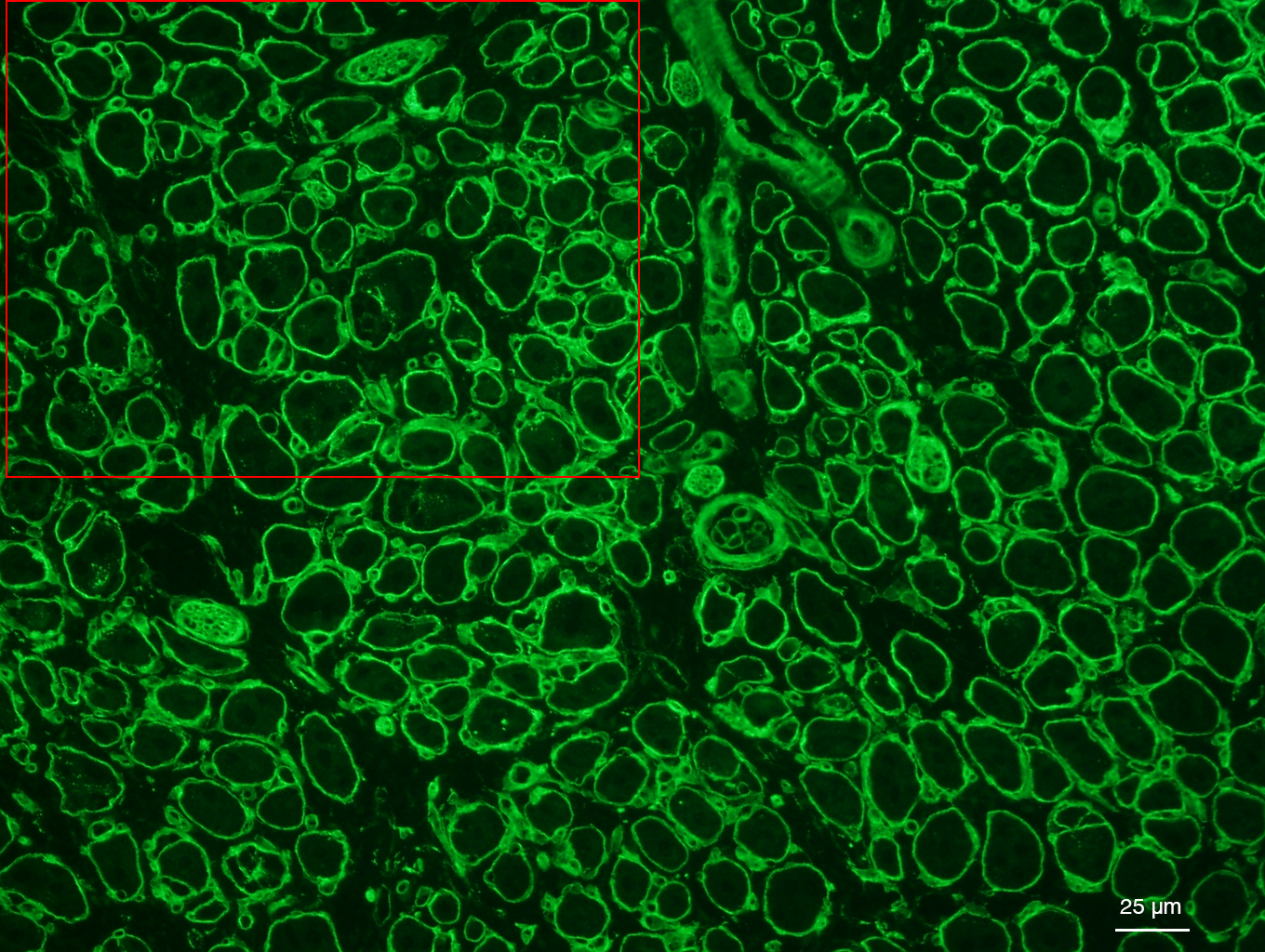

Supplement: Supplementary file 10 — Source data Fig. 6 [file 44318_2025_663_MOESM10_ESM.zip › EMBOJ-2025-121889_SourceDataForFigure6/6F/Matr3 mutant mice (mpA)/Laminin.tif]

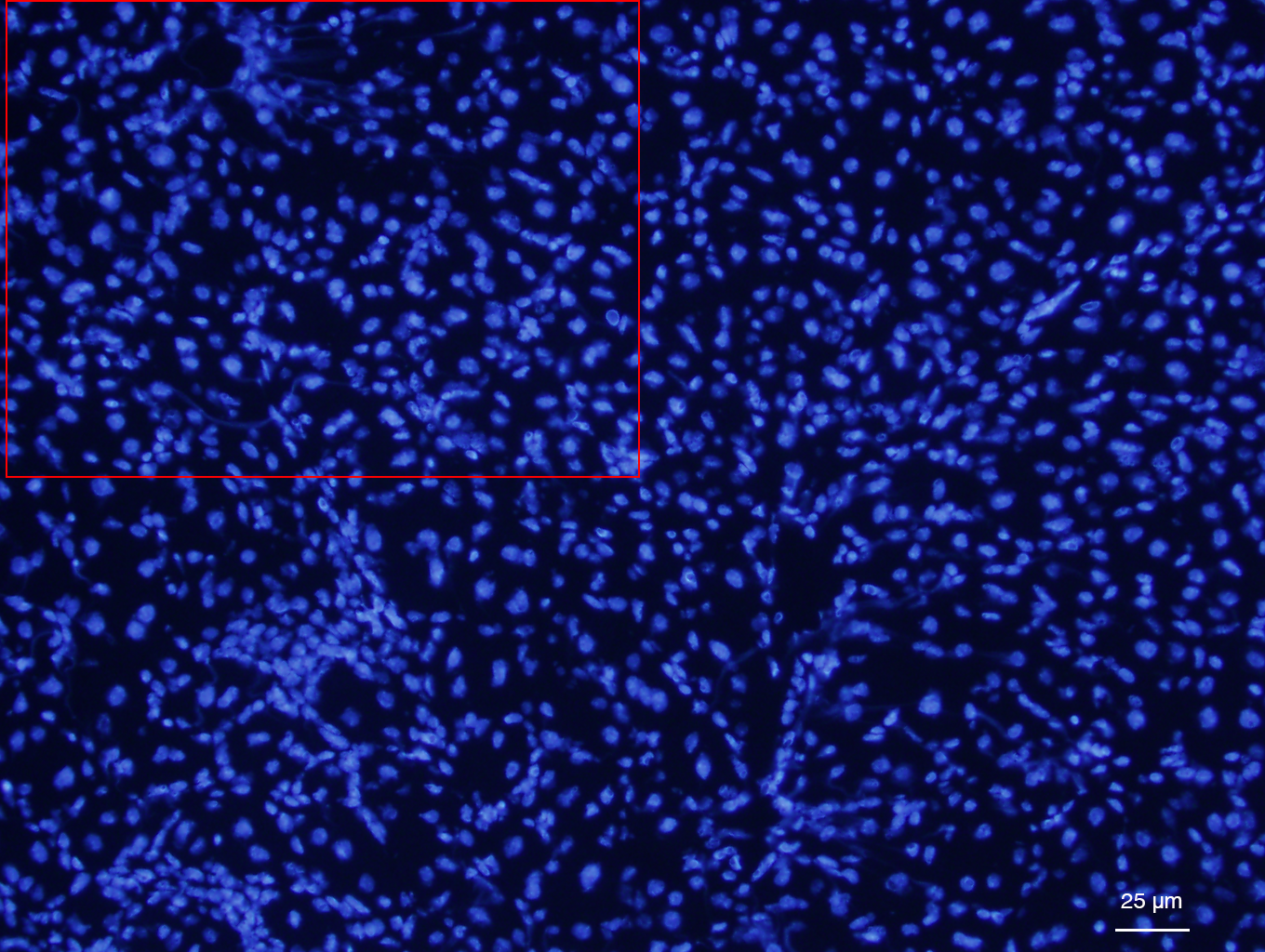

Supplement: Supplementary file 10 — Source data Fig. 6 [file 44318_2025_663_MOESM10_ESM.zip › EMBOJ-2025-121889_SourceDataForFigure6/6F/WT mice (+)/DAPI.tif]

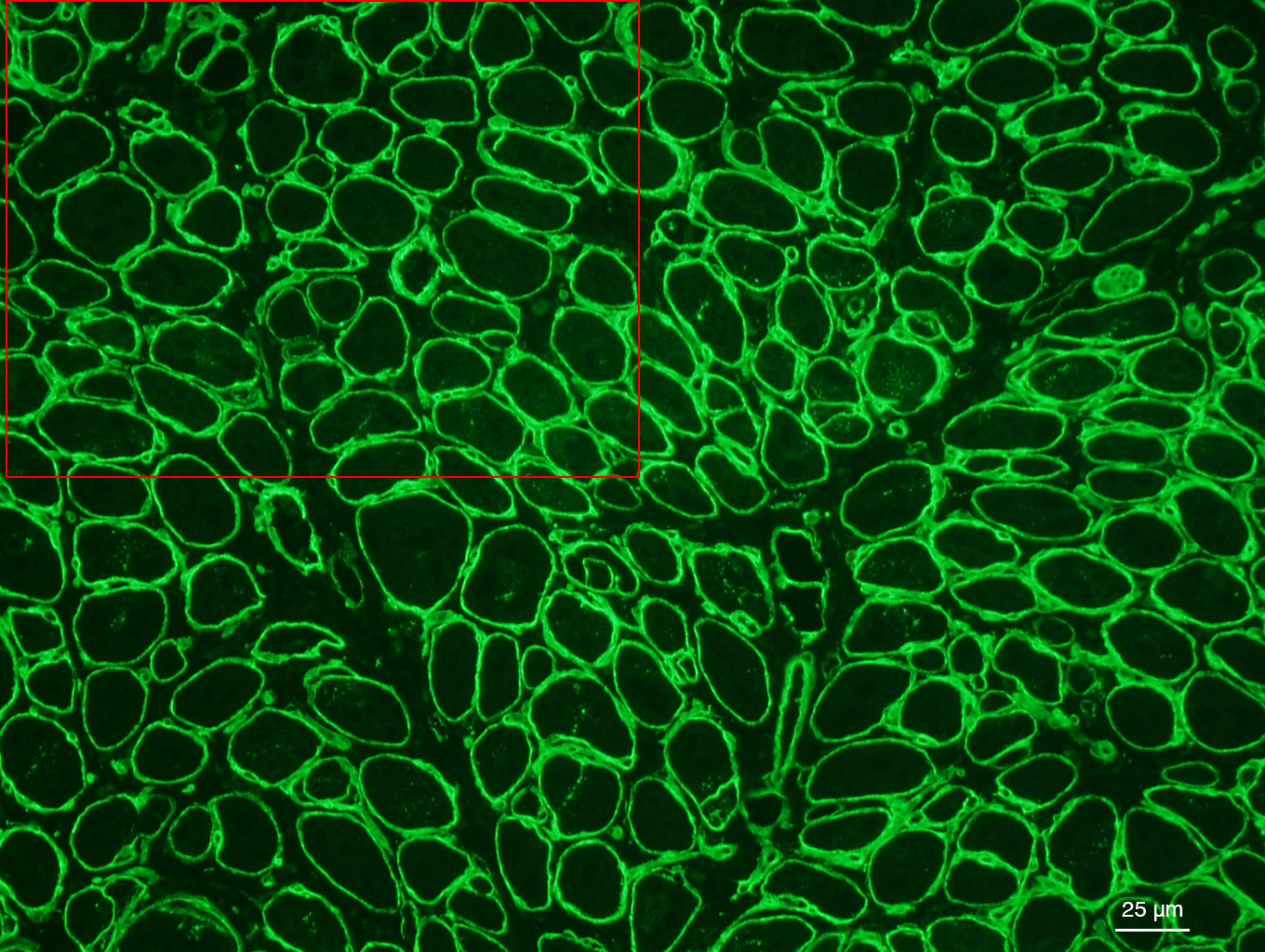

Supplement: Supplementary file 10 — Source data Fig. 6 [file 44318_2025_663_MOESM10_ESM.zip › EMBOJ-2025-121889_SourceDataForFigure6/6F/WT mice (+)/MyHC.tif]

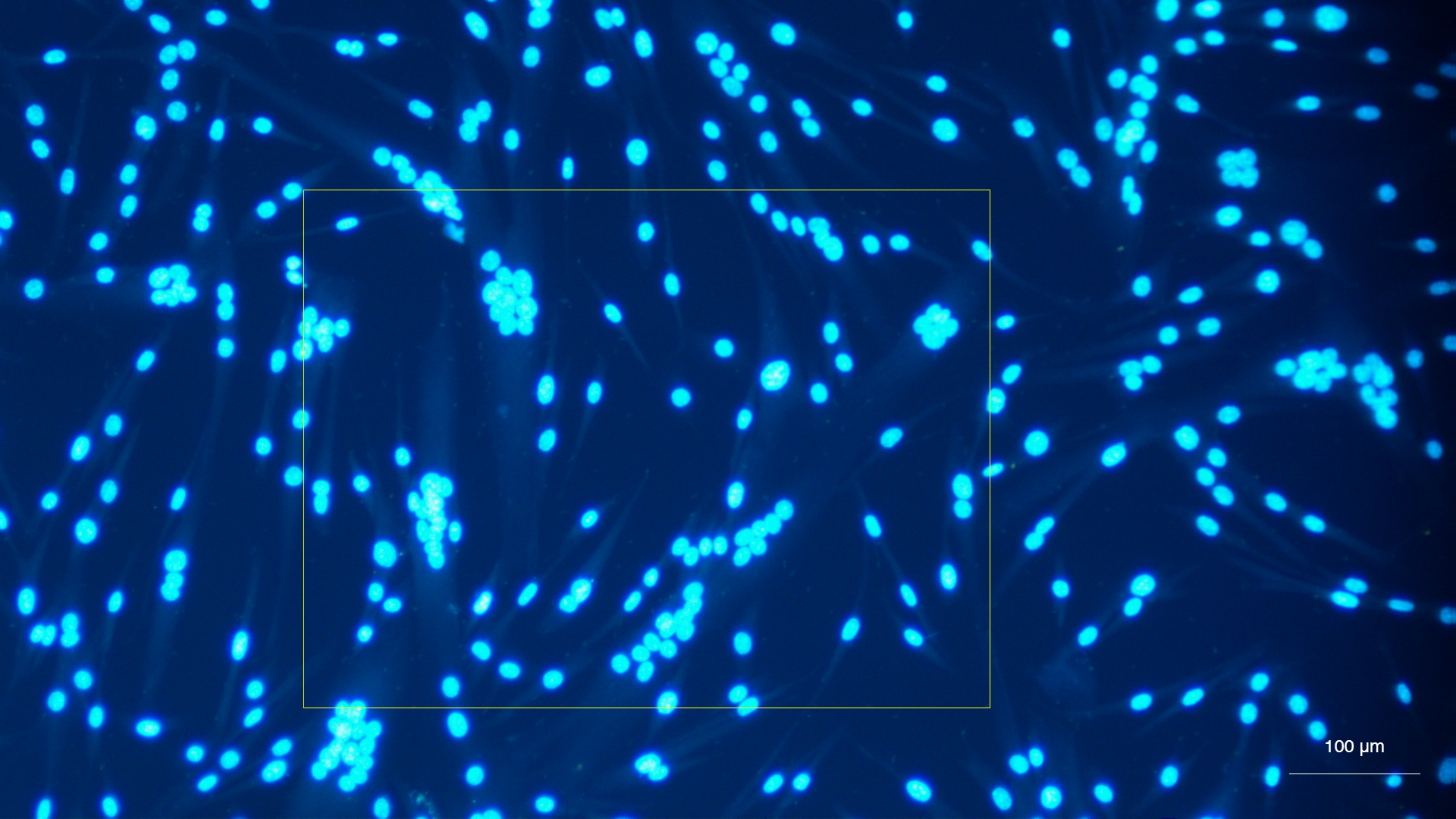

Supplement: Supplementary file 10 — Source data Fig. 6 [file 44318_2025_663_MOESM10_ESM.zip › EMBOJ-2025-121889_SourceDataForFigure6/6H/Matr3 mutant mice (mpA)/DAPI.tif]

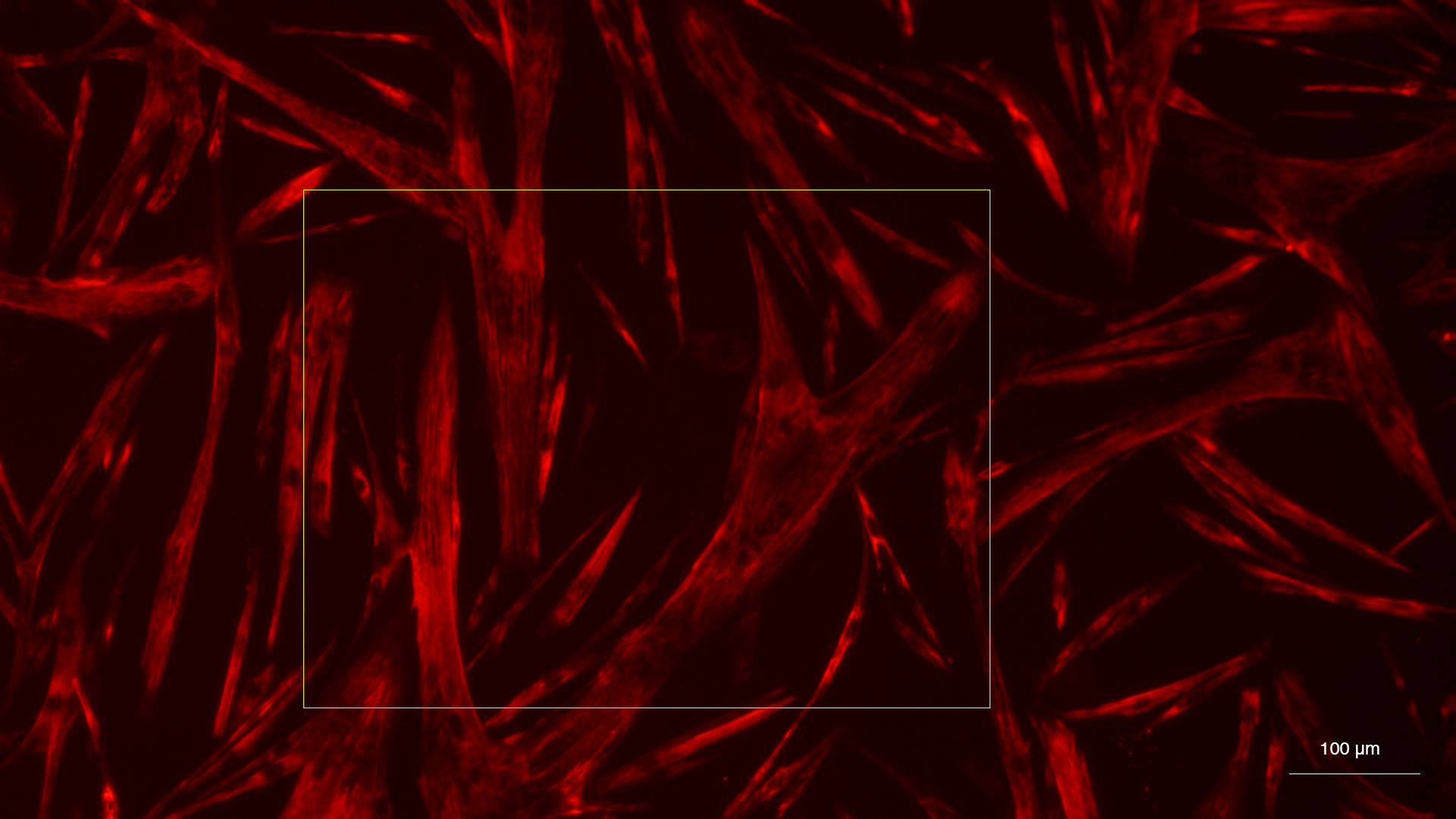

Supplement: Supplementary file 10 — Source data Fig. 6 [file 44318_2025_663_MOESM10_ESM.zip › EMBOJ-2025-121889_SourceDataForFigure6/6H/Matr3 mutant mice (mpA)/MyHC.tif]

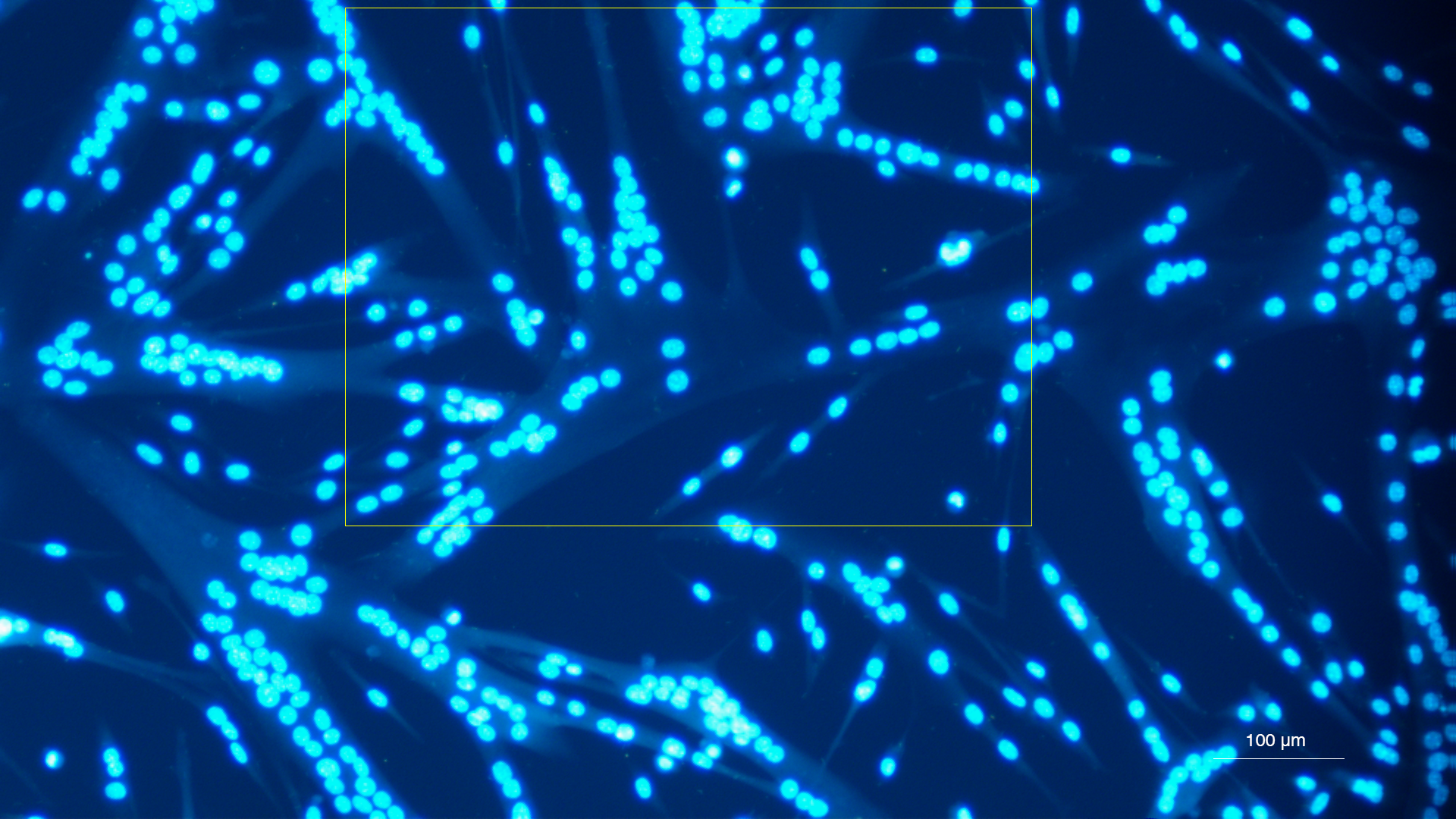

Supplement: Supplementary file 10 — Source data Fig. 6 [file 44318_2025_663_MOESM10_ESM.zip › EMBOJ-2025-121889_SourceDataForFigure6/6H/WT mice (+)/DAPI.tif]

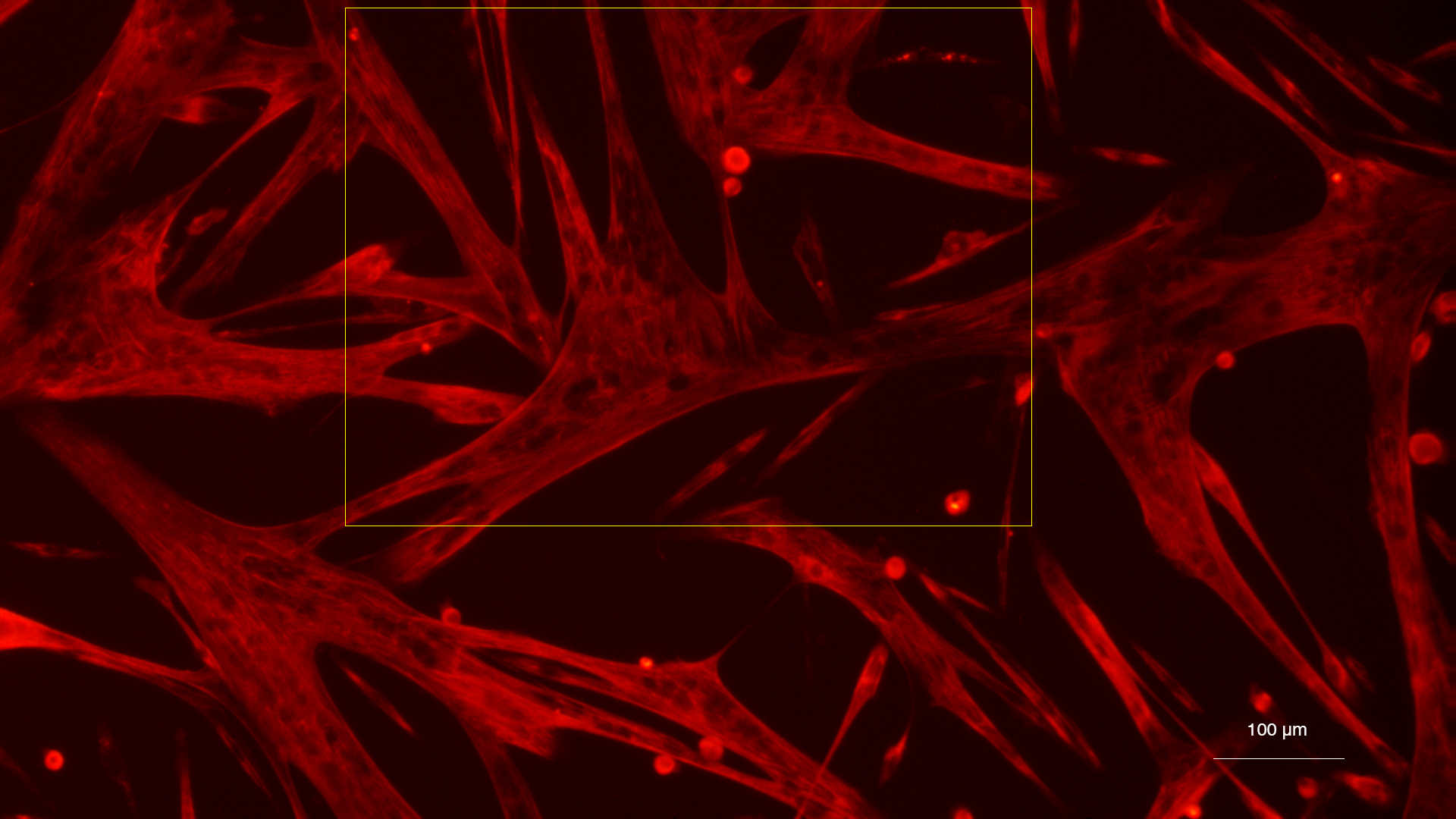

Supplement: Supplementary file 10 — Source data Fig. 6 [file 44318_2025_663_MOESM10_ESM.zip › EMBOJ-2025-121889_SourceDataForFigure6/6H/WT mice (+)/MyHC.tif]

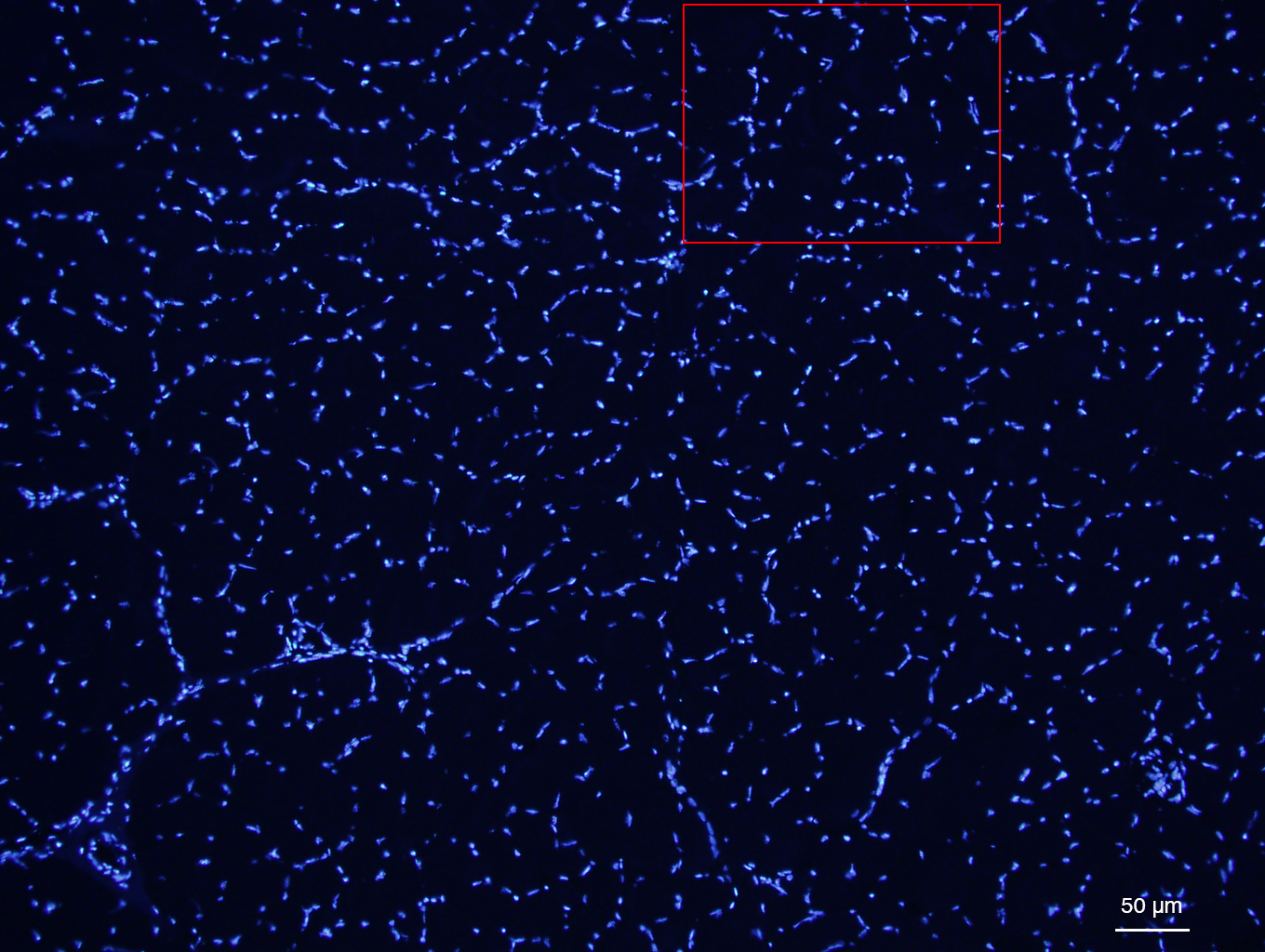

Supplement: Supplementary file 11 — Source data for Expanded View Figures and Appendix Figures [file 44318_2025_663_MOESM11_ESM.zip › Fig EV3/EV3 F/Matr3 mutant mice (mpA)/DAPI.tif]

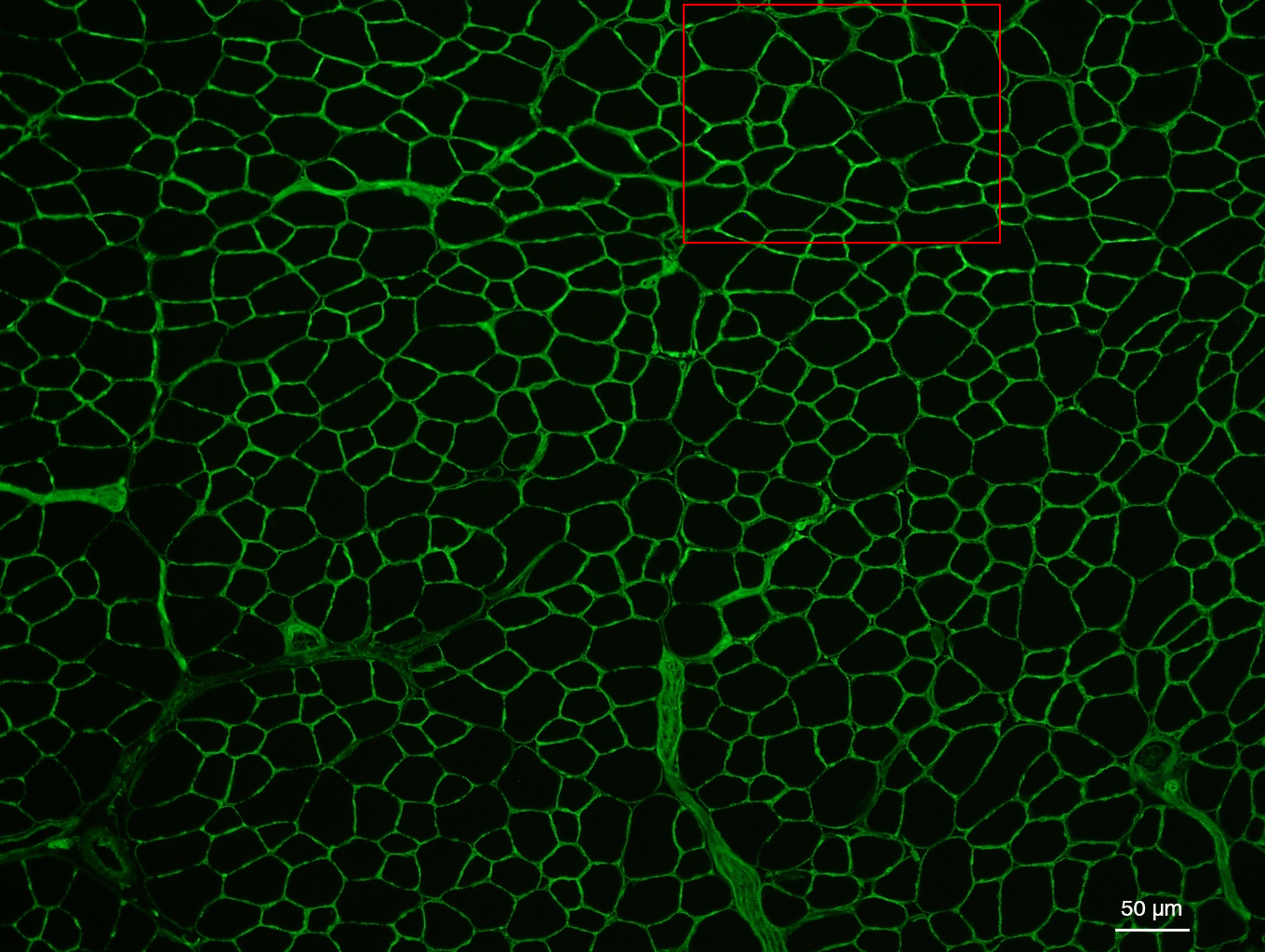

Supplement: Supplementary file 11 — Source data for Expanded View Figures and Appendix Figures [file 44318_2025_663_MOESM11_ESM.zip › Fig EV3/EV3 F/Matr3 mutant mice (mpA)/Laminin.tif]

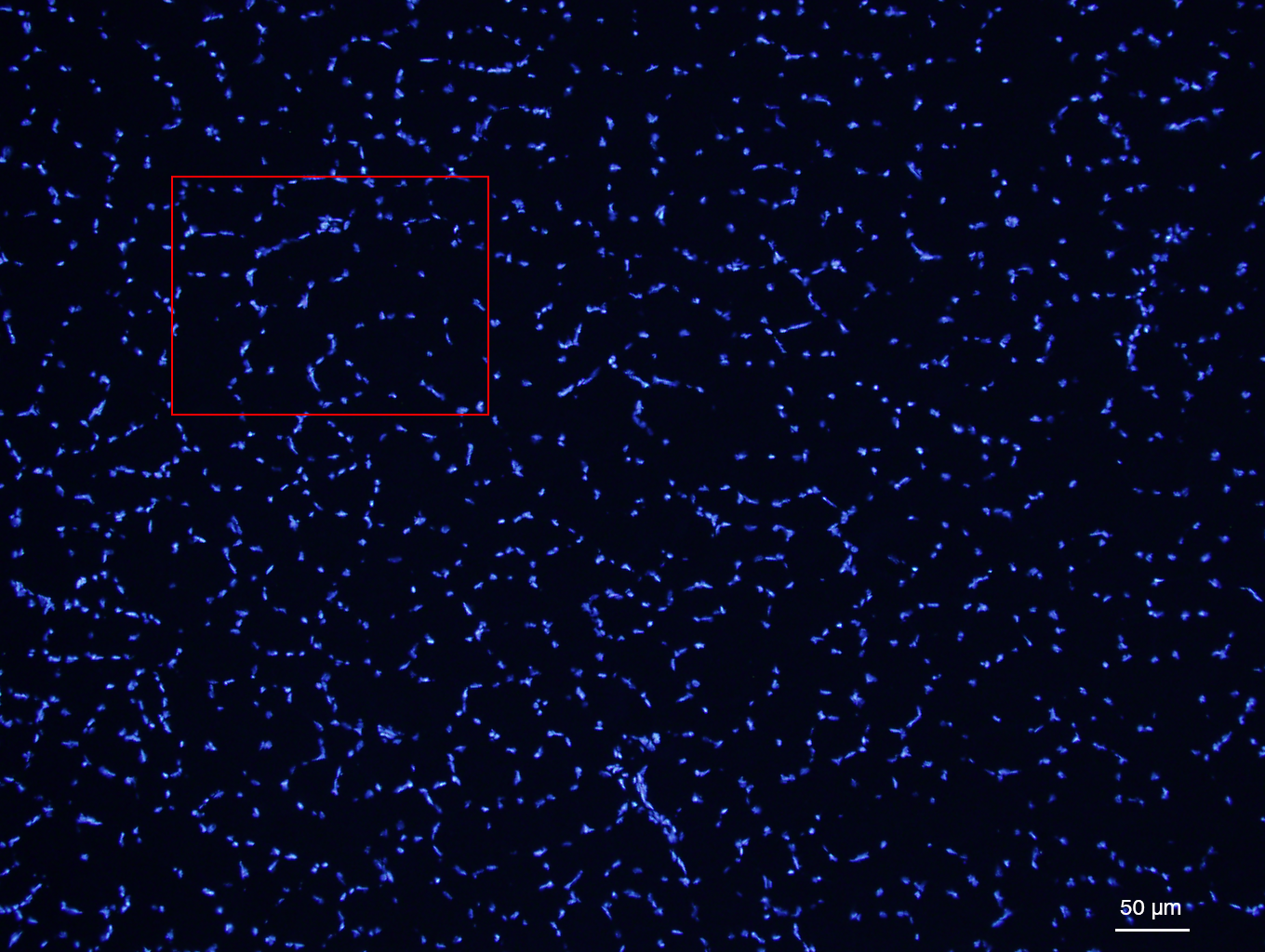

Supplement: Supplementary file 11 — Source data for Expanded View Figures and Appendix Figures [file 44318_2025_663_MOESM11_ESM.zip › Fig EV3/EV3 F/WT mice (+)/DAPI.tif]

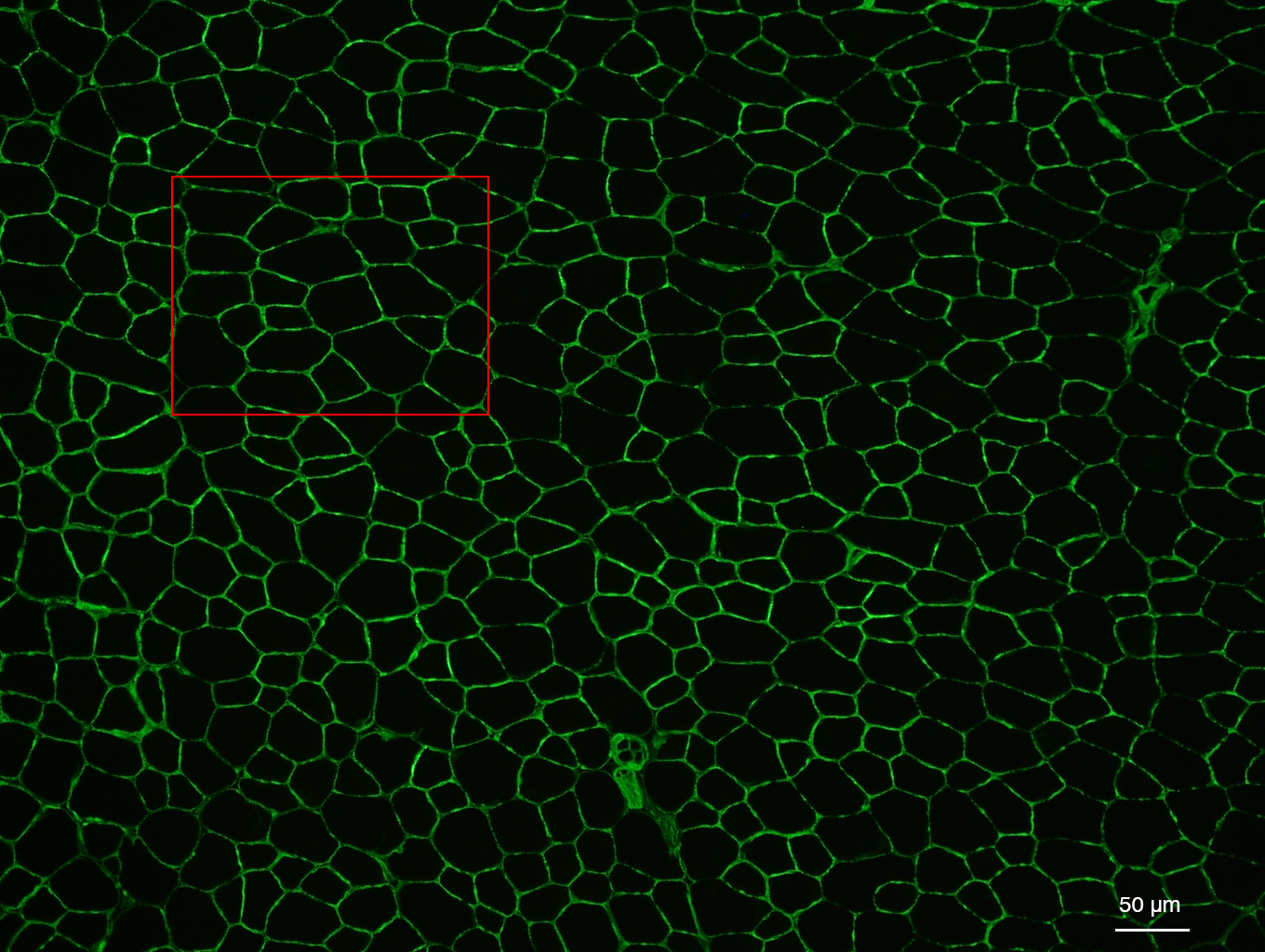

Supplement: Supplementary file 11 — Source data for Expanded View Figures and Appendix Figures [file 44318_2025_663_MOESM11_ESM.zip › Fig EV3/EV3 F/WT mice (+)/Laminin.tif]

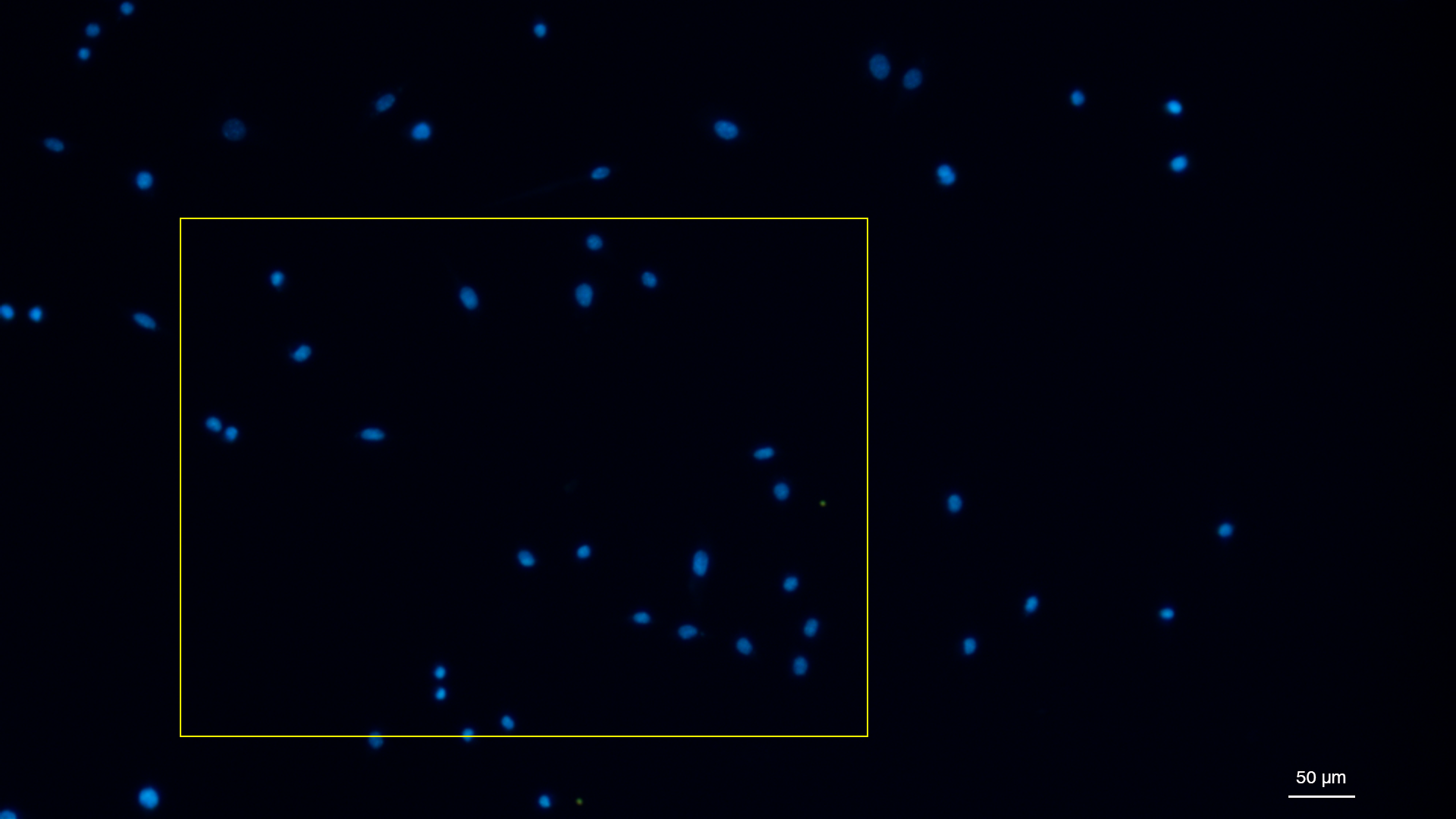

Supplement: Supplementary file 11 — Source data for Expanded View Figures and Appendix Figures [file 44318_2025_663_MOESM11_ESM.zip › Fig EV3/EV3 K/Matr3 mutant mice (mpA)/DAPI.tif]

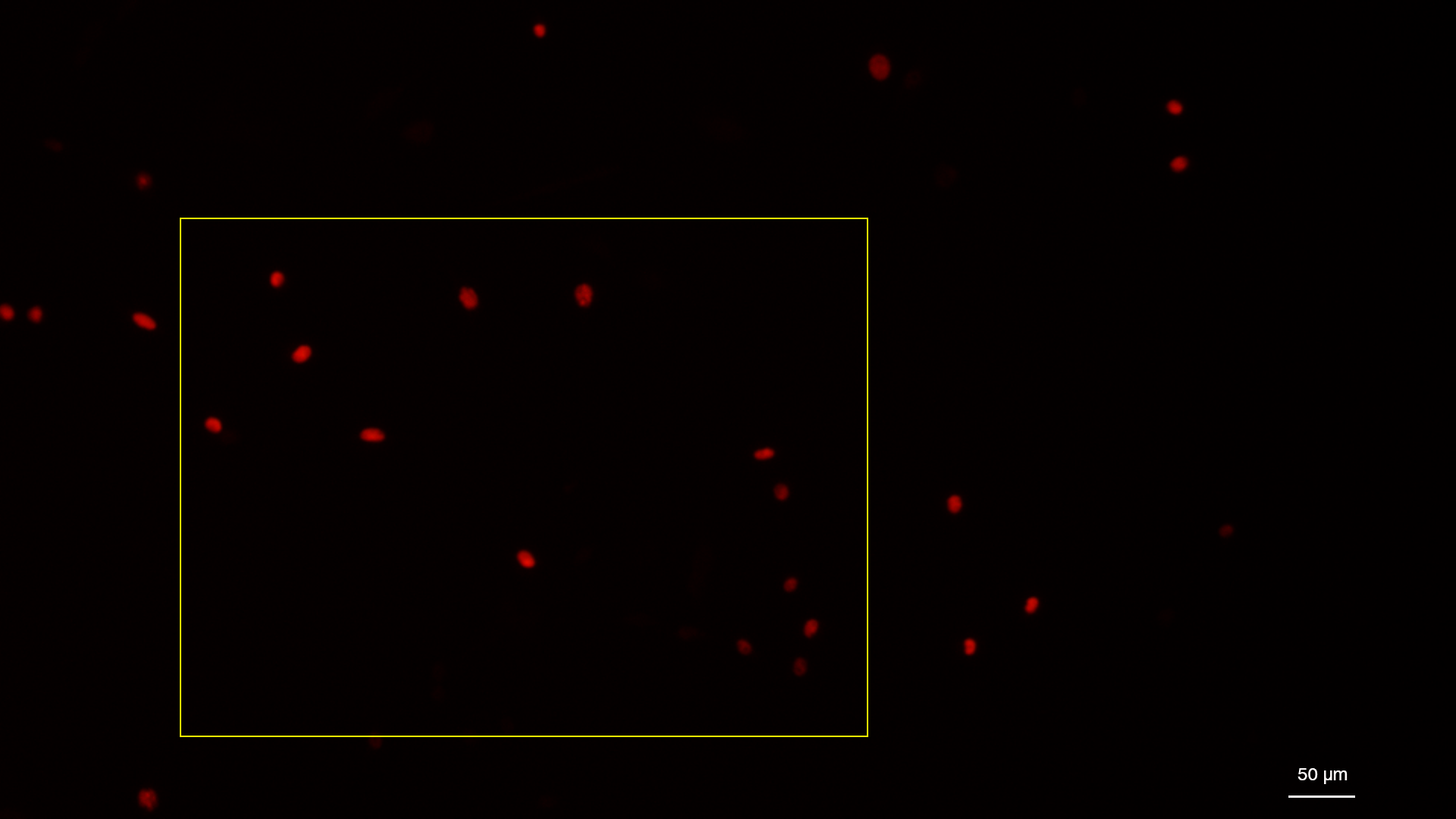

Supplement: Supplementary file 11 — Source data for Expanded View Figures and Appendix Figures [file 44318_2025_663_MOESM11_ESM.zip › Fig EV3/EV3 K/Matr3 mutant mice (mpA)/EdU.tif]

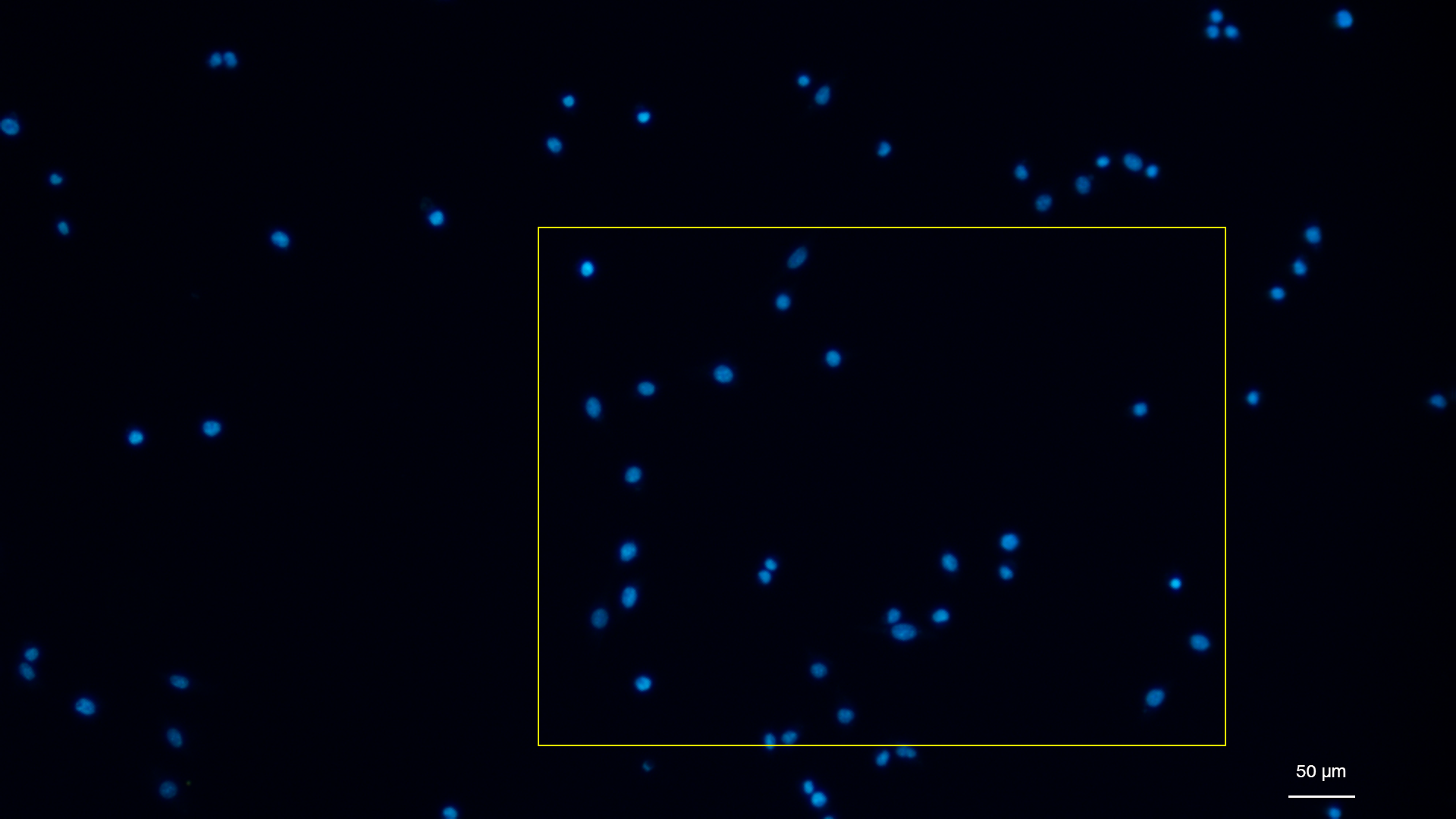

Supplement: Supplementary file 11 — Source data for Expanded View Figures and Appendix Figures [file 44318_2025_663_MOESM11_ESM.zip › Fig EV3/EV3 K/WT mice (+)/DAPI.tif]

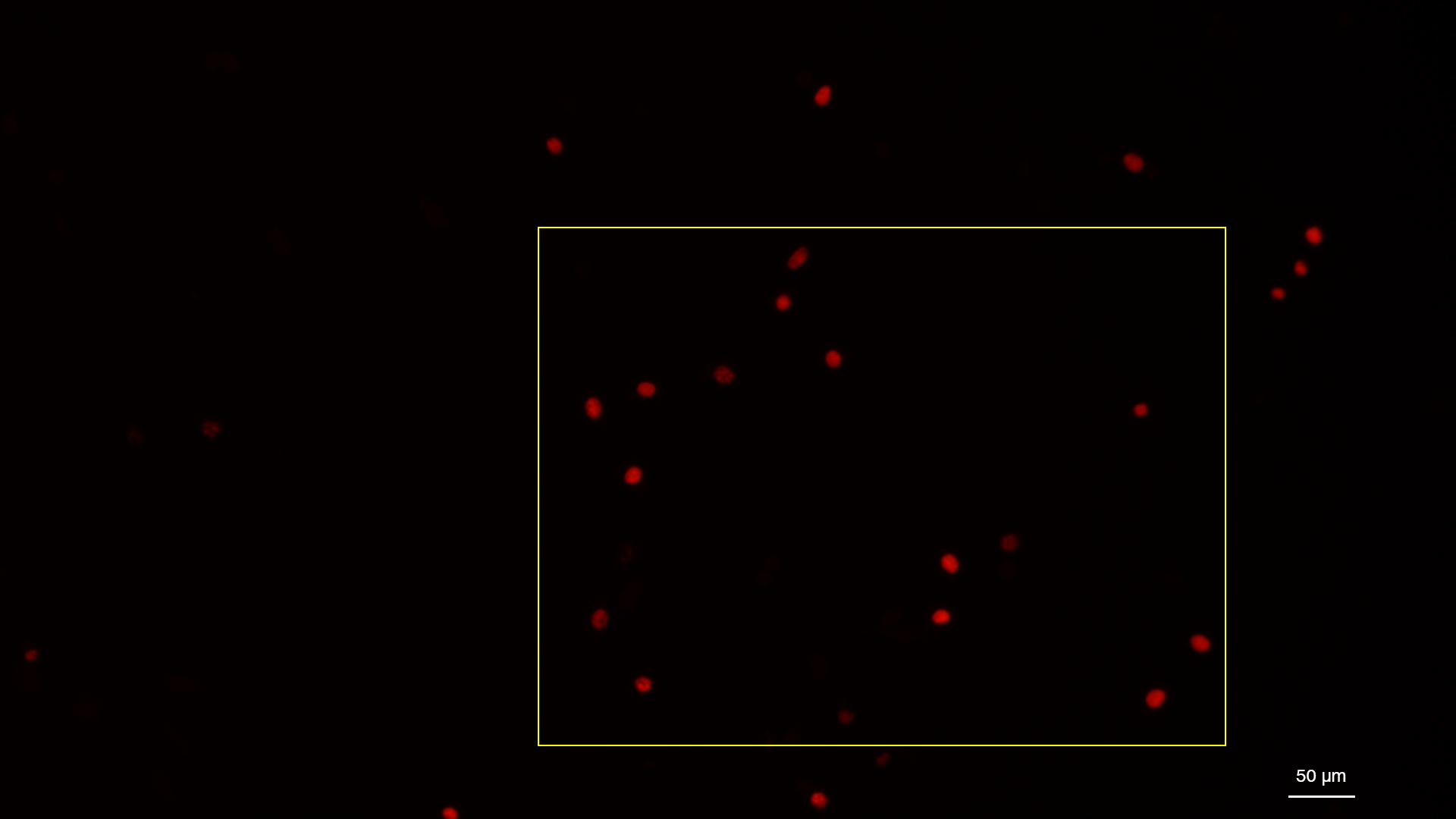

Supplement: Supplementary file 11 — Source data for Expanded View Figures and Appendix Figures [file 44318_2025_663_MOESM11_ESM.zip › Fig EV3/EV3 K/WT mice (+)/EdU.tif]

Source data for **Appendix Fig S2C**

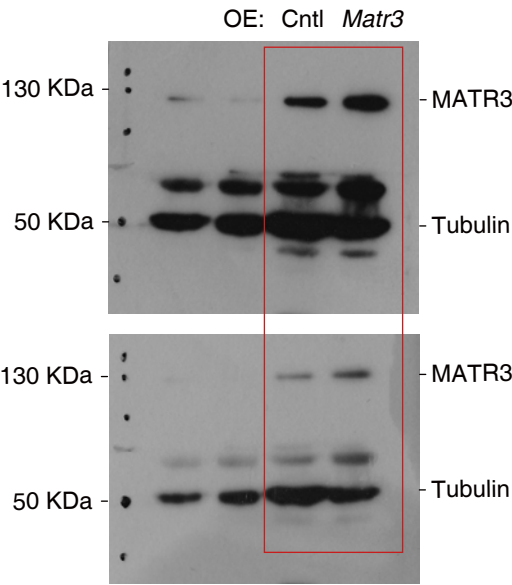

Supplement: Supplementary file 11 — Source data for Expanded View Figures and Appendix Figures [file 44318_2025_663_MOESM11_ESM.zip › Appendix Fig S2/S2C/Western blot for S2C.pdf]
