# Supplementary material for: 3′UTR shortening alleviates miRNA repression of mRNAs critical for muscle stem cell differentiation
Source: EMBO J. 2025 Dec 22;45(3):722–48. doi: 10.1038/s44318-025-00663-2 (PMC12864877; doi:10.1038/s44318-025-00663-2)
Supplement: Supplementary file 12 — Expanded View Figures [file 44318_2025_663_MOESM12_ESM.pdf]

Expanded View Figures

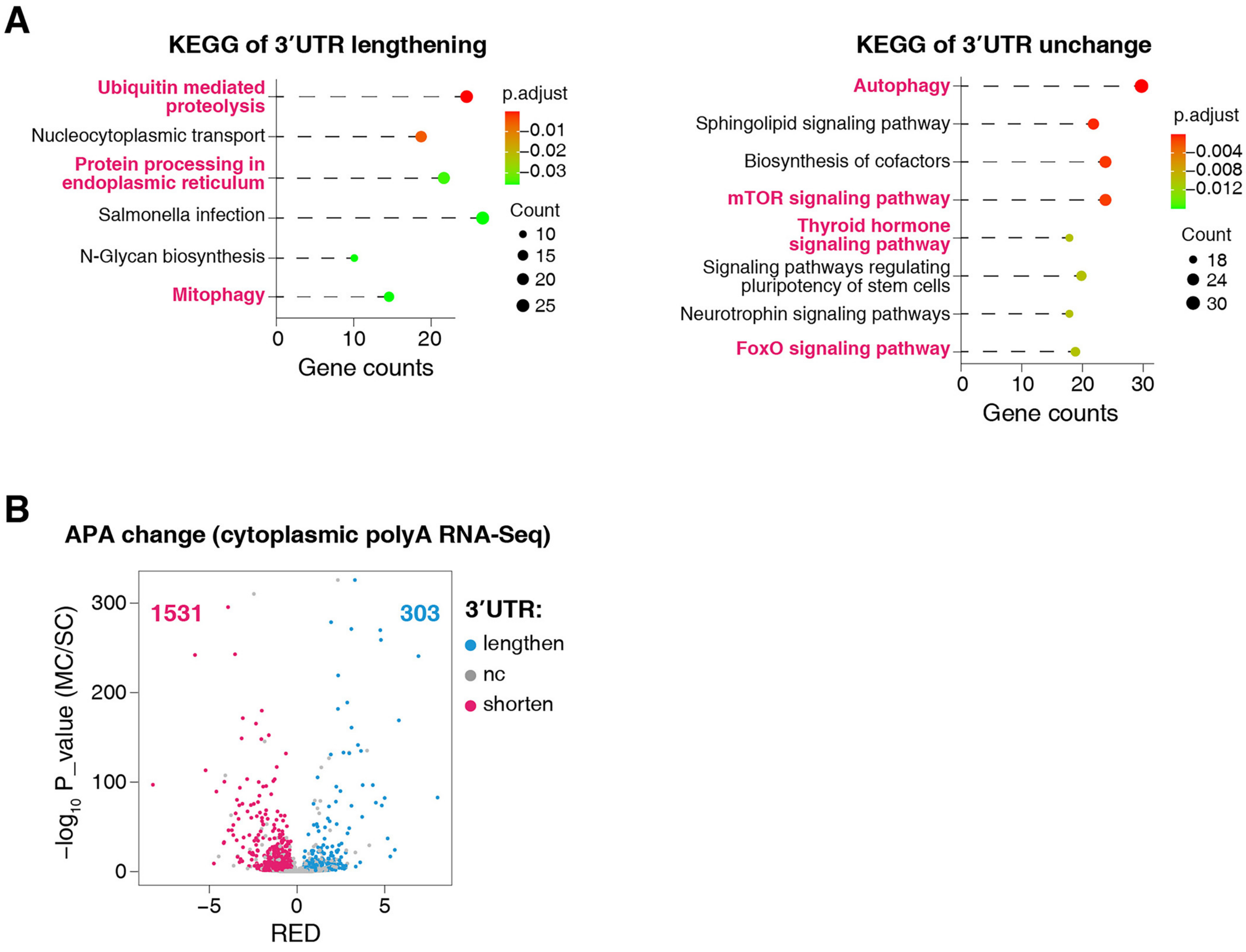

Figure EV1. Preferential 3′UTR shortening during satellite cell (SC) differentiation.

(**A**) KEGG pathway enrichment analysis of genes with lengthened (left) and unchanged (right) RNA 3′UTRs. *P*-values were calculated using a hypergeometric test and adjusted for multiple hypothesis testing via the Benjamini-Hochberg method. (**B**) Volcano plot showing the APA changes in MC versus SC. APA changes were quantified using RED (relative expression difference, Δlog_2[d-pA/p-pA]) (Wang and Tian, 2020). Statistically significant APA events were identified using a false discovery rate (FDR)-adjusted *p*-value < 0.05 (Fisher's exact test). Genes exhibiting significant 3′UTR shortening and lengthening are indicated in magenta and blue. Gene counts are shown.

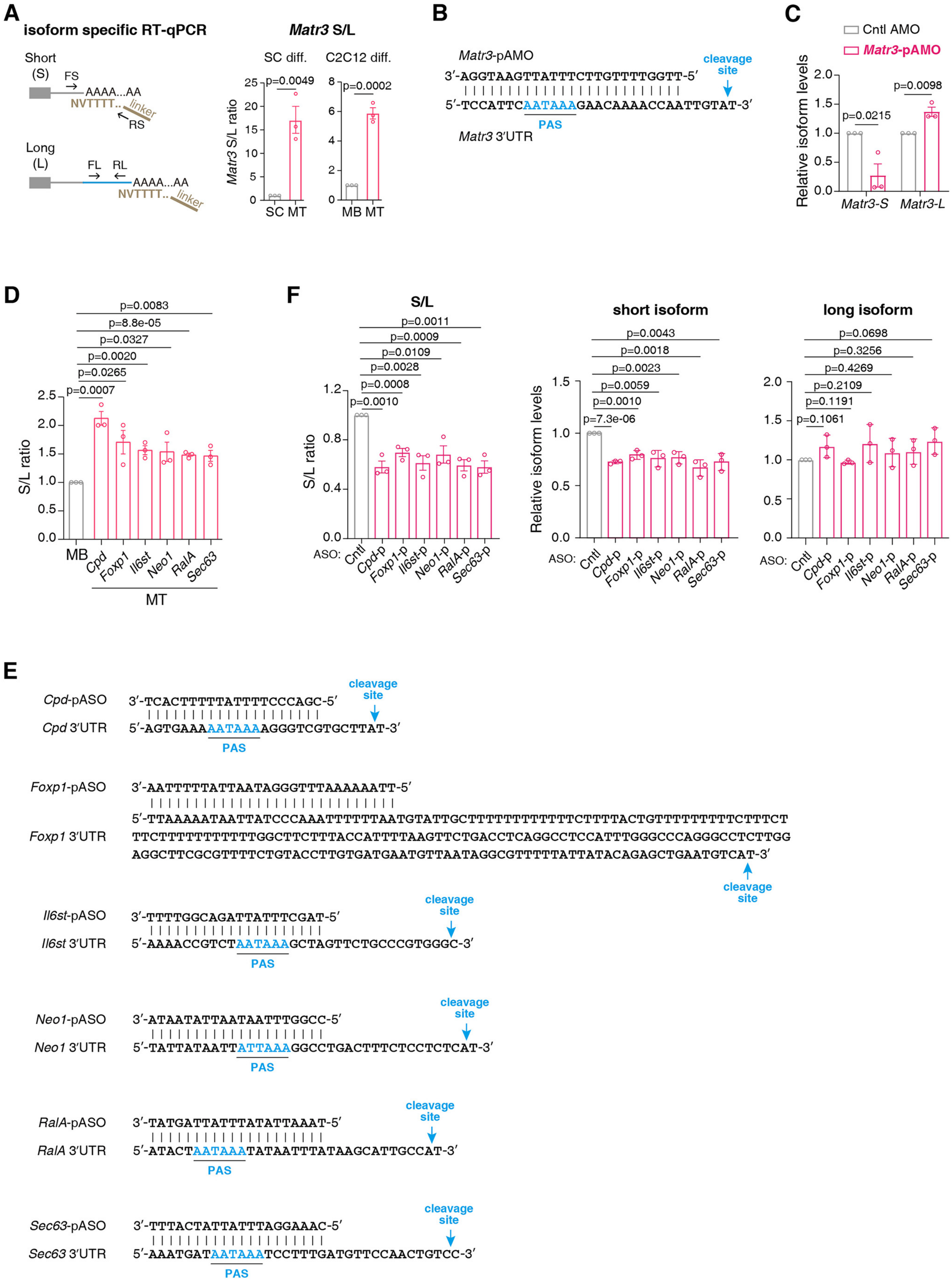
A
isoform specific RT-qPCR
Short (S)
FS
AAAA...AA
NVTTTT..
linker
RS
Long (L)
FL
RL
Matr3 S/L
SC diff.
C2C12 diff.
p=0.0049
p=0.0002
Matr3 S/L ratio
SC MT
MB MT
B
Matr3-pAMO
3′-AGGTAAGTTATTTCTTGTTTTGGTT-5′
5′-TCCATTCAATAAAGAACAAAACCAATTGTAT-3′
PAS
Matr3 3′UTR
cleavage site
C
Cntl AMO
Matr3-pAMO
Relative isoform levels
p=0.0215
p=0.0098
Matr3-S
Matr3-L
D
p=0.0083
p=8.8e-05
p=0.0327
p=0.0020
p=0.0265
p=0.0007
S/L ratio
MB
Cpd
Foxp1
Il6st
Neo1
RalA
Sec63
MT
F
S/L
p=0.0011
p=0.0009
p=0.0109
p=0.0028
p=0.0008
p=0.0010
ASO:
Cntl
Cpd-p
Foxp1-p
Il6st-p
Neo1-p
RalA-p
Sec63-p
short isoform
p=0.0043
p=0.0018
p=0.0023
p=0.0059
p=0.0010
p=7.3e-06
Relative isoform levels
long isoform
p=0.0698
p=0.3256
p=0.4269
p=0.2109
p=0.1191
p=0.1061
E
Cpd-pASO
3′-TCACTTTTTATTTTCCCAGC-5′
Cpd 3′UTR
5′-AGTGAAAAATAAAAGGGTCGTGCTTAT-3′
PAS
cleavage site
Foxp1-pASO
3′-AATTTTTATTAATAGGGTTTAAAAAATT-5′
Foxp1 3′UTR
5′-TTAAAAATAATTATCCCAAATTTTTTAATGTATTGCTTTTTTTTTTTTTCTTTTACTGTTTTTTTTTTCTTTCT
TTCTTTTTTTTTTTTGGCTTCTTTACCATTTTAAGTTCTGACCTCAGGCCTCCATTTGGGCCCAGGGCCTCTTGG
AGGCTTCGCGTTTTCTGTACCTTGTGATGAATGTTAATAGGCGTTTTTATTATACAGAGCTGAATGTCAT-3′
cleavage site
Il6st-pASO
3′-TTTTGGCAGATTATTTCGAT-5′
Il6st 3′UTR
5′-AAAACCGTCTAATAAAGCTAGTTCTGCCCGTGGGC-3′
PAS
cleavage site
Neo1-pASO
3′-ATAATATTAATAATTTGGCC-5′
Neo1 3′UTR
5′-TATTATAATTATTAAAGGCCTGACTTTCTCCTCTCAT-3′
PAS
cleavage site
RalA-pASO
3′-TATGATTATTTATATTAAAT-5′
RalA 3′UTR
5′-ATACTAATAAATATAATTTATAAGCATTGCCAT-3′
PAS
cleavage site
Sec63-pASO
3′-TTTACTATTATTTAGGAAAC-5′
Sec63 3′UTR
5′-AAATGATAATAAATCCTTTGATGTTCCAACTGTCC-3′
PAS
cleavage site

 © The Author(s)

◀ **Figure EV2. Inhibition of 3′UTR shortening impairs myogenic differentiation.**

(**A**) Schematic of the isoform-specific RT-qPCR strategy (left). Reverse transcription primers are indicated in bronze. FS, forward primer of short 3′UTR isoform; RS, reverse primer of short 3′UTR isoform; FL, forward primer of long 3′UTR isoform; RL, reverse primer of long 3′UTR isoform. RT-qPCR to measure the ratio of short to long (S/L) *Matr3* isoforms during SC differentiation and MB differentiation (right) ($n = 3$). Error bars, mean ± SEM. *P*-values, two-sided unpaired student's t test. (**B**) Sequences of the *Matr3*-pAMO and its complementary target within the 3′UTR of *Matr3* are shown. The polyadenylation signal (PAS) and cleavage sites are indicated. (**C**) Isoform-specific RT-qPCR to measure short and long isoform levels of *Matr3* in MTs differentiated from SCs treated with either control AMO (Cntl AMO) or *Matr3*-pAMO ($n = 3$). Bars represent RNA abundance normalized to *Gapdh*. Error bars, mean ± SEM. *P*-values, two-sided unpaired student's t test. (**D**) Isoform-specific RT-qPCR to measure the short to long (S/L) isoform ratio of indicated genes in MBs and differentiated MTs ($n = 3$). Error bars, mean ± SEM. *P*-values, two-sided unpaired student's t test. (**E**) Sequences of the indicated ASOs and their complementary 3′UTR regions. PAS and cleavage sites are indicated. (**F**) Isoform-specific RT-qPCR to measure the S/L ratios, short and long isoform levels of the indicated genes in MTs differentiated from MBs treated with ASOs blocking the proximal pA site of the corresponding gene ($n = 3$). For results of short and long isoform levels, bar represent RNA abundance normalized to *Gapdh*. Error bars, mean ± SEM. *P*-values, two-sided unpaired student's t test. Source data are available online for this figure.

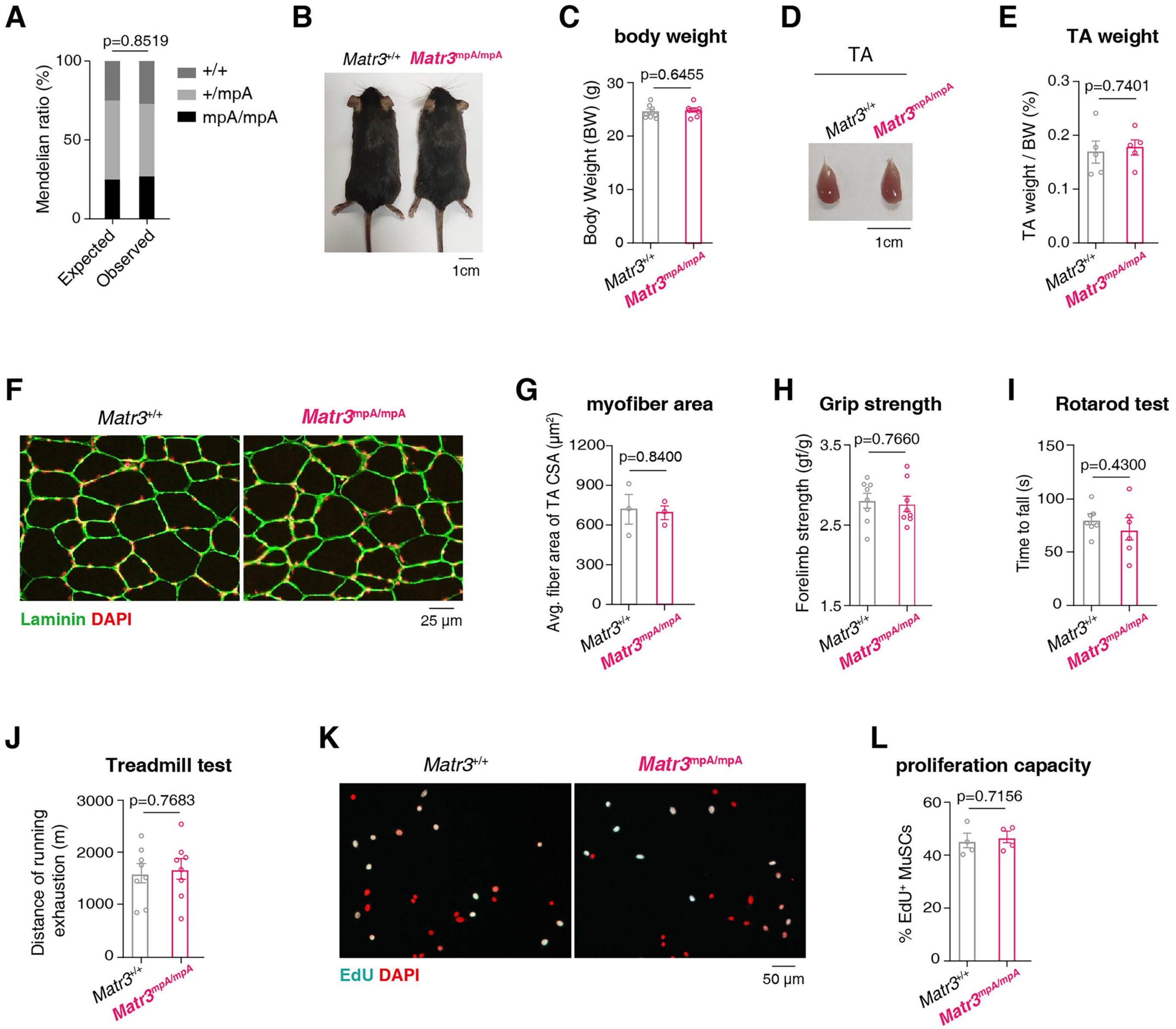

Figure EV3. *Matr3*$^{mpA/mpA}$ mice exhibited normal skeletal muscle development and motor function.

(**A**) *Matr3*$^{mpA/mpA}$ mice were born at the expected Mendelian ratio ($n = 108$). No significant differences between the expected and observed genotypic numbers, as determined by chi-squared test. (**B**) Representative photographs of 8-week-old *Matr3*$^{+/+}$ and *Matr3*$^{mpA/mpA}$ littermates. Scale bar, 1 cm. (**C**) Bar plot quantifying the body weights of 8-week-old *Matr3*$^{+/+}$ and *Matr3*$^{mpA/mpA}$ littermates ($n = 8$). Error bars, mean ± SEM. *P*-values, two-sided unpaired student's t test. (**D**) Representative photographs of TA muscles from *Matr3*$^{+/+}$ and *Matr3*$^{mpA/mpA}$ littermates at 12 weeks of age. Scale bar, 1 cm. (**E**) Bar plot showing the ratio of TA muscle weight to body weight (BW) in *Matr3*$^{+/+}$ and *Matr3*$^{mpA/mpA}$ littermates at 12 weeks of age ($n = 5$). Error bars, mean ± SEM. *P*-values, two-sided unpaired student's t test. (**F**) Representative immunofluorescence staining images of Laminin (green) and DAPI (red) of TA muscle cross-sections from *Matr3*$^{+/+}$ and *Matr3*$^{mpA/mpA}$ littermates at 12 weeks of age. Scale bar, 25 μm. (**G**) Quantification of average cross-section area (CSA) of TA myofibers from *Matr3*$^{+/+}$ and *Matr3*$^{mpA/mpA}$ littermates at 12 weeks of age ($n = 3$). Error bars, mean ± SEM. *P*-values, two-sided unpaired student's t test. (**H**) Forelimb grip strength (normalized to body weight) was measured in *Matr3*$^{+/+}$ and *Matr3*$^{mpA/mpA}$ littermates at 12 weeks of age ($n = 8$). Error bars, mean ± SEM. *P*-values, two-sided unpaired student's t test. (**I**) Rotarod tests were assessed in *Matr3*$^{+/+}$ ($n = 7$) and *Matr3*$^{mpA/mpA}$ ($n = 6$) littermates at 12 weeks of age. The time it takes for the mouse to fall off the rod was recorded. Error bars, mean ± SEM. *P*-values, two-sided unpaired student's t test. (**J**) Distances of running exhaustion were measured in *Matr3*$^{+/+}$ and *Matr3*$^{mpA/mpA}$ littermates at 4-5 months of age ($n = 8$). Error bars, mean ± SEM. *P*-values, two-sided unpaired student's t test. (**K**) Representative images of EdU (cyan) incorporation in SCs isolated from *Matr3*$^{+/+}$ and *Matr3*$^{mpA/mpA}$ littermates. DAPI (red) was used for nuclear counterstaining. Scale bar, 50 μm. (**L**) Quantification of SC proliferation capacity from *Matr3*$^{+/+}$ and *Matr3*$^{mpA/mpA}$ littermates ($n = 4$). The ratio of EdU-positive (Edu^+) SCs to the total number of cells (DAPI) was calculated. Error bars, mean ± SEM. *P*-values, two-sided unpaired student's t test. Source data are available online for this figure.

© The Author(s)

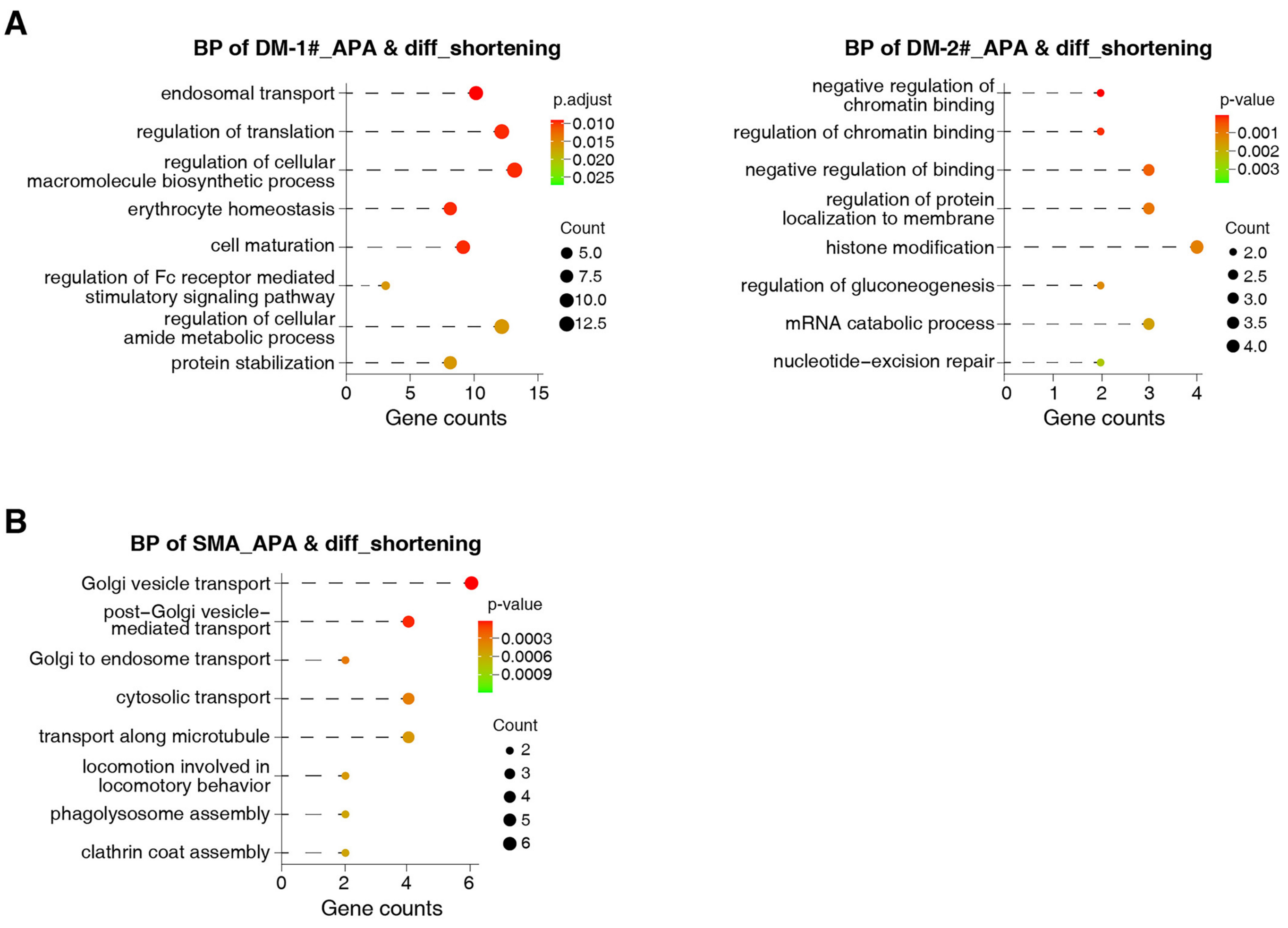

Figure EV4. Dysregulated 3′UTR shortening in muscle/neuromuscular diseases.

(**A**) Dot plot showing Gene Ontology (Biological Process, BP) terms enriched in genes exhibiting 3′UTR shortening upon differentiation that also display APA changes in DM samples. (**B**) Dot plot showing Gene Ontology (Biological Process, BP) terms enriched in genes exhibiting 3′UTR shortening upon differentiation that also display APA changes in SMA samples. For dot plots in (**A**) and (**B**), *p*-values were calculated using a hypergeometric test and adjusted for multiple hypothesis testing via the Benjamini-Hochberg method.

© The Author(s)
